# Essential Role of Chromatin Remodeling Protein Bptf in Early Mouse Embryos and Embryonic Stem Cells

**DOI:** 10.1371/journal.pgen.1000241

**Published:** 2008-10-31

**Authors:** Joseph Landry, Alexei A. Sharov, Yulan Piao, Lioudmila V. Sharova, Hua Xiao, Eileen Southon, Jennifer Matta, Lino Tessarollo, Ying E. Zhang, Minoru S. H. Ko, Michael R. Kuehn, Terry P. Yamaguchi, Carl Wu

**Affiliations:** 1 Laboratory of Biochemistry and Molecular Cell Biology, National Cancer Institute, National Institutes of Heath, Bethesda, Maryland, United States of America; 2 Developmental Genomics and Aging Section, National Institute on Aging, National Institutes of Health, Baltimore, Maryland, United States of America; 3 Mouse Cancer Genetics Program, National Cancer Institute, National Institutes of Health, Frederick, Maryland, United States of America; 4 Laboratory Animal Science Program, SAIC-Frederick, NCI-Frederick, Frederick, Maryland, United States of America; 5 Laboratory of Cellular and Molecular Biology, National Cancer Institute, National Institutes of Heath, Bethesda, Maryland, United States of America; 6 Laboratory of Protein Dynamics and Signaling, National Cancer Institute, National Institutes of Health, Frederick, Maryland, United States of America; 7 Cancer and Developmental Biology Laboratory, National Cancer Institute, National Institutes of Health, Frederick, Maryland, United States of America; Massachusetts General Hospital, Howard Hughes Medical Institute, United States of America

## Abstract

We have characterized the biological functions of the chromatin remodeling protein Bptf (Bromodomain PHD-finger Transcription Factor), the largest subunit of NURF (Nucleosome Remodeling Factor) in a mammal. *Bptf* mutants manifest growth defects at the post-implantation stage and are reabsorbed by E8.5. Histological analyses of lineage markers show that *Bptf^−/−^* embryos implant but fail to establish a functional distal visceral endoderm. Microarray analysis at early stages of differentiation has identified *Bptf*-dependent gene targets including homeobox transcriptions factors and genes essential for the development of ectoderm, mesoderm, and both definitive and visceral endoderm. Differentiation of *Bptf^−/−^* embryonic stem cell lines into embryoid bodies revealed its requirement for development of mesoderm, endoderm, and ectoderm tissue lineages, and uncovered many genes whose activation or repression are *Bptf-*dependent. We also provide functional and physical links between the Bptf-containing NURF complex and the Smad transcription factors. These results suggest that Bptf may co-regulate some gene targets of this pathway, which is essential for establishment of the visceral endoderm. We conclude that Bptf likely regulates genes and signaling pathways essential for the development of key tissues of the early mouse embryo.

## Author Summary

While the chromatin of eukaryotes provides an efficient means to compact large amounts of DNA into a small nucleus, it renders the DNA relatively inaccessible. ATP-dependent chromatin remodeling complexes mobilize nucleosomes and provide a means to gain access to DNA in chromatin. While the biochemical functions of chromatin remodeling complexes is well-characterized, less is known of their biological functions. In this manuscript, we elucidate the biological functions of Bptf, a subunit of the NURF chromatin remodeling complex. Our studies show that *Bptf* is required for the establishment of the anterior–posterior axis of the mouse embryo during the earliest stages of development. To understand its functions in tissue differentiation, we generated and characterized *Bptf*-mutant ES cells. Mutant embryonic stem cells show significant defects in the differentiation of ectoderm, endoderm, and mesoderm. Genome-wide analysis of gene expression defects during differentiation has identified many *Bptf*-dependent pathways including key regulators of ectoderm, endoderm, and mesoderm differentiation. Moreover, we have identified critical functions for *Bptf* during the TGFβ/Smad-induced expression of visceral endoderm and mesoderm markers, an important signaling pathway in the early embryo. These results suggest that chromatin remodeling by *Bptf* regulates key signaling pathways in the early mouse embryo.

## Introduction

The packaging of eukaryotic DNA into chromatin provides a general mechanism for the modulation of gene activity and DNA metabolism through alterations of chromatin architecture. The structure and composition of chromatin can be altered by a number of distinct pathways, including post-translational modification of histones, ATP-dependent remodeling of nucleosomes, and incorporation of histone variants [1]–[3]. ATP-dependent chromatin remodeling is catalyzed by the large and conserved SWI/SNF super family of multi-subunit chromatin remodeling enzymes that are classified into four major subfamilies (SWI/SNF, ISWI, CHD, and INO80), and distinguished by the common presence of a SWI2/SNF2-related catalytic ATPase subunit [4],[5].

The mammalian ISWI chromatin remodeling complexes contain either one of two related ISWI ATPases, Snf2l and Snf2h [6],[7]. The Snf2l ATPase is contained in two assemblies–NURF (Nucleosome Remodeling Factor), which is dedicated to the regulation of transcription, and the recently reported CERF [8],[9]. NURF is the founding member of the ISWI family of chromatin remodeling complexes, and was originally characterized in Drosophila [10]. Purified Drosophila NURF catalyzes ATP-dependent nucleosome sliding and promotes transcription from chromatin templates *in vitro* [9]. As shown by whole genome expression studies of mutants, NURF positively or negatively regulates transcription of several hundred Drosophila genes *in vivo*, including many genes important for fly development [11]. This is likely accomplished through recruitment of NURF301, the largest NURF subunit, by gene-specific transcription factors [11]–[13], and binding of a PHD finger of NURF301 to tri-methylated lysine 4 on histone H3 [14]. Human NURF contains the orthologs of three of four Drosophila NURF components–BPTF (Bromodomain PHD-finger Transcription Factor), the mammalian counterpart of NURF301, SNF2L (the ISWI ATPase) and RbAp46/48, a WD-40 repeat histone-binding protein found in several chromatin-related protein complexes [15]. Biochemical studies of human NURF have shown that it has similar properties to its Drosophila counterpart [15].

The physiological functions of an increasing number of mammalian chromatin remodeling complexes have been revealed by studies of mouse mutants for the catalytic ATPase. Mutations in *Brg1, Brm, Chd4, Chd2, p400* and *Etl1* have been shown to be required for proper embryonic development, hematopoiesis or postnatal survival [16]–[22]. A mutant for the Snf2h, one of the two murine ISWI ATPases, revealed severe proliferation defects in the early embryo, resulting in a peri-implantation lethal phenotype [23]. Given the presence of Snf2h in multiple chromatin remodeling complexes, the assignment of biological phenotypes to different enzyme complexes can be problematic [6],[7].

By analysis of mutations of unique subunits it is possible to identify the biological functions of different Snf2h-containing complexes. This has been accomplished for the Drosophila ISWI complexes. Studies of mutants for Drosophila *NURF301*, which is exclusive to the NURF complex [12], have revealed a late larval-lethal phenotype and mis-expression of homeotic selector genes and genes involved in the response to heat stress, cytokine and steroid hormone signals [11],[13]. These phenotypes do not overlap with those observed for mutants for Drosophila *ACF1* (a component of the ISWI-containing complexes ACF and CHRAC) which are impaired in the establishment and/or maintenance of transcriptional silencing in pericentric heterochromatin and in repression by *Polycomb*-group genes [24].

To elucidate the biological roles of Bptf-containing complexes we have generated embryonic stem cell and mouse mutants for *Bptf* [15]. Our studies show that *Bptf* mutant phenotypes begin to manifest just after implantation stage, and mutant embryos are completely reabsorbed by embryonic day (E) 8.5. Genetic and molecular analysis in embryonic stem cells and the mouse suggest a role for Bptf in the development of visceral endoderm (VE) of the early mammalian embryo. We propose that Bptf is required for the development of the VE, and more importantly the distal visceral endoderm (DVE), in part through regulating cellular proliferation, the expression of homeobox-containing transcription factors and pathways regulated by the Smad transcription factors, a major conduit for cell signaling in development. These findings suggest a model in which the activities of Bptf-containing complexes, likely the NURF remodeling complex, regulate cell proliferation and embryonic development and therefore are essential in the post-implantation embryo.

## Results/Discussion

### Characterization of Gene-Trap and Conditional Alleles of *Bptf*

BPTF, the mammalian ortholog of Drosophila NURF301, is a large, multi-domain protein that is apparently exclusive to the mammalian NURF complex (Figure S1A) [12],[15],[25]. An initial characterization of Bptf showed it to be nuclear in the P19 embryonic carcinoma cell line and can exist in at least two electrophoretic variants, which we termed Bptf-H and Bptf-L (Figure S1B, Figure S1C). As previously reported, Bptf is highly expressed in testis, spleen, brain and to a lesser extent in kidney by Western blotting (Figure S1D). Interestingly Bptf is highly expressed during embryonic development, and expression substantially declines upon birth (Figure S1E). The high levels of Bptf expression in the mouse embryo suggest it may have essential functions during embryonic development.

To elucidate the biological functions of mammalian Bptf during mammalian embryonic development we generated two mutant mouse lines. One line, designated as *Bptf^XG023^*, was derived from an ES cell line carrying an in-frame gene-trap vector insertion between exons 15 and 16 of *Bptf* (Figure S2A, Figure S2B, S2C, S2D, S2E, S2F) (*XG023*; http://www.genetrap.org/) [26]. We identified the precise junction of the insertion site of the gene trap by DNA sequence analysis of the corresponding PCR products, and confirmed by RT-PCR that trapping of *Bptf* mRNA into vector sequences leads to loss of RNA splicing between exons 15 and 16, and reduced expression of *Bptf* sequences 3′ to the insertion site (Figure S3A, Figure S3B). In addition, we confirmed by 5′-RACE that the insertion resulted in an in-frame fusion between *Bptf* and *β-galactosidase-neomycin phosphotransferase* (*β-G*eo) sequences of the gene-trap vector. (Figure S3C). Consistent with previous findings, Northern blotting of adult tissue RNA showed that both wild-type *Bptf* and the *Bptf–β-Geo* fusion alleles are specifically expressed at high levels in the testis and at moderate levels in the lung, spleen, and brain (Figure S3D) [25]. These identical RNA expression patterns initially suggest that β-galactosidase is a faithful reporter of *Bptf* expression in adult tissues. These results indicate that the *Bptf^XG023^* mutation creates a truncated Bptf–β-Geo fusion carrying the N-terminal 1978 residues of the 2903-residue Bptf open reading frame, which eliminates the conserved glutamine rich region, PHD finger, and bromo-domains.

The second line, designated as *Bptf^ΔExon2^*, was generated by targeting loxP sites flanking exon 2 of *Bptf* (Figure S2A, Figure S2G, Figure S2H, Figure S2I, Figure S2J, Figure S2K, Figure S2L, Figure S2M, Figure S2N). The expression of the *Bptf^ΔExon2^* allele was assessed by RT-PCR with the use of primer sets which amplify sequences on the 3′ end of the *Bptf* transcript. We found that the *Bptf^ΔExon2^* allele slightly decreases expression of *Bptf* at the RNA level (Figure S4A). However, amplification and sequencing of the *Bptf^ΔExon2^* mRNA from exons 1 to 8 shows an out-of-frame mutant mRNA, indicating that *Bptf^ΔExon2^* behaves as a loss-of-function allele (Figure S4B, Figure S4C).

### Post-Implantation Phenotypes of *Bptf* Mutant Embryos

We intercrossed heterozygous mice to determine the biological function of *Bptf* during mouse development. With 210 mice genotyped for the *Bptf^XG023^* allele and 75 mice for the *Bptf^ΔExon2^* allele, we did not find any surviving homozygous mice at weaning, indicating that *Bptf* is required for mouse development. An analysis of E6.5 to E18.5 progeny derived from heterozygous intercrosses confirmed that the homozygous mutant phenotype is embryonic-lethal between E7.5 to E8.5, with 100% penetrance (Table S1).

**Figure 1:**
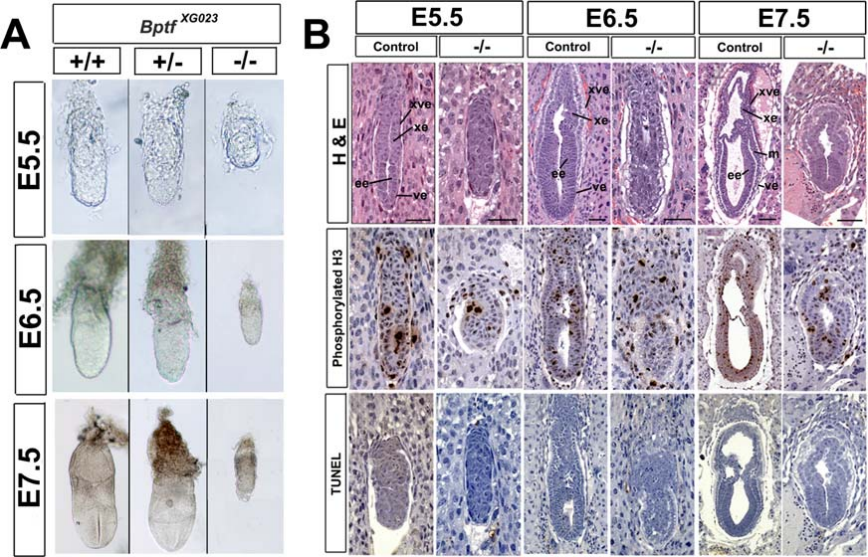
*Bptf* mutants manifest early embryonic growth defects. (A) Wild type (+/+), heterozygous (+/−), and homozygous (−/−)*Bptf ^XG023^* embryos at E5.5, E6.5, and E7.5 were removed from their decedua and genotyped using the PCR based method. Reduced growth rates are evident in the homozygous *Bptf ^XG023^* embryos as early as E5.5. (B) Hematoxylin and eosin (H&E) staining of E5.5, E6.5, and E7.5 mutant embryos show reduced growth of both embryonic and extra-embryonic tissues, the presence of visceral endoderm, and an absence of anterior–posterior asymmetry when compared to controls. *Bptf* mutant embryos were stained for phosphorylated histone H3, a marker of cell mitosis. Mutant embryos at E5.5, E6.5, and E7.5 have significantly reduced staining when compared to controls, suggesting that they have reduced cellular proliferation. *Bptf-*mutant embryos were stained for apoptotic cells using the TUNEL assay. Mutant embryos at E5.5, E6.5, and E7.5 do not have any positive staining by TUNEL when compared to controls, suggesting that they are not apoptotic. Scale Bars: E5.5 and E6.5 embryos = 50 µm, E7.5 embryos = 100 µm. Abbreviations: ve, visceral endoderm; ee, embryonic ectoderm; m, mesoderm; xe, extra-embryonic ectoderm; xve, extra-embryonic visceral endoderm.

To identify defects in the embryonic development of *Bptf^XG023^* and *Bptf^ΔExon2^* homozygotes, we performed whole mount examinations of mutant embryos. Growth defects increasing in severity from E5.5 to E7.5 were observed (Figure 1A) (Figure S5A). Dissections conducted at E8.5 and E9.5 were uninformative, because a majority of the embryos had been completely reabsorbed (data not shown). We crossed *Bptf^XG023^* and *Bptf^ΔExon2^* heterozygous mice to generate the trans-heterozygous *Bptf^XG023^*/*Bptf^ΔExon2^* embryos. The trans-heterozygote recapitulated the growth defects of the *Bptf^XG023^* and *Bptf^ΔExon2^* homozygotes, indicating that the two mutations are functionally equivalent (Figure S5B). To determine whether the developmental defects originated before implantation, we harvested E3.5 blastocysts and performed blastocyst outgrowth assays. We observed normal E3.5 homozygous *Bptf^XG023^* and *Bptf^ΔExon2^* blastocysts and normal outgrowths from the blastocysts after tissue culture for 5 days, suggesting that either *Bptf* is not essential for pre-implantation development or that the maternal Bptf protein or mRNA can mask pre-implantation phenotypes (Figure S6). The masking of pre-implantation phenotypes by maternal Bptf is possible because it is highly expressed in oocytes [27],[28].

To further investigate the basis of the early embryonic lethal phenotype, we performed a histological analysis of *Bptf^XG023^* mutant embryos at E5.5, E6.5 and E7.5. Mid-sagittal sections of E5.5, E6.5 and E7.5 embryos stained with hematoxylin and eosin (H&E) showed a distinct proximal-distal (P-D) axis and the development of visceral endoderm but a significant decrease in size of the embryonic and extra-embryonic tissues. This was particularly evident in the embryonic ectoderm at E6.5 and E7.5 (Figure 1B). By E6.5, mutant embryos showed clear developmental defects. Although there was a clear boundary between extra embryonic and embryonic tissues, the absence of a primitive streak and any discernable mesoderm suggests that the anterior-posterior (A-P) axis did not form (Figure 1B).

A reduction in cell number can be a consequence of decreased cell proliferation, increased cell death (apoptosis), or a combination of both processes. To ascertain the extent of programmed cell death, we performed TUNEL assays on E5.5, E.6.5 and E7.5 homozygotes and found no increased numbers of apoptotic cells when compared to controls (Figure 1B). As a measure of cell proliferation we monitored phosphorylated histone H3 levels in the conceptus by immuno-histochemistry (Figure 1B). E5.5, E6.5 and E7.5 homozygotes showed ∼40–50% decrease in phosphorylated histone H3 levels indicating that a decrease in cellular proliferation may contribute to the mutant phenotype (Figure S7).

### *Bptf* Mutants Fail To Develop a Functional Distal Visceral Endoderm

Subsequent to implantation of the mouse blastocyst there is rapid proliferation of the egg cylinder, which consists of three cell types: the more proximal extra-embryonic ectoderm, the more distal embryonic ectoderm or epiblast, and an outer layer of visceral endoderm [29]. The visceral endoderm originates from the primitive endoderm, a layer of cells organized at E4.5, which is composed of cells from the ICM of the E3.5 blastocyst expressing *Gata6* but not *Nanog* [30]. At ∼E5.5, a specialized cluster of endoderm cells, the DVE, arises at the distal tip of the embryo. DVE cells migrate toward the prospective anterior, to form the anterior visceral endoderm (AVE). DVE/AVE cells secrete molecules such as cerberus (Cer1) and Lefty1, antagonists of the Transforming Growth Factor β (TGFβ)-related protein Nodal [29]. These antagonists restrict the activity of Nodal to the posterior pole of the embryo at E6.0 [31]. The primitive streak forms at E6.5, indicating that gastrulation has begun, and gives rise to the mesoderm and definitive endoderm germ layers [29].

**Figure 2:**
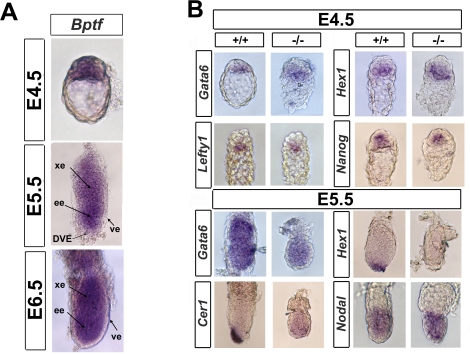
*Bptf* mutants are defective in the expression of distal visceral endoderm markers. (A) Wild type embryos were stained in whole mount for *Bptf* mRNA by *in situ* RNA hybridization at E4.5, E5.5, and E6.5. *Bptf* is expressed in the inner cell mass and primitive endoderm at E4.5 and in the embryonic and extra-embryonic tissues in E5.5 and E6.5 embryos. Abbreviations: ve, visceral endoderm; ee, embryonic ectoderm; xe, extra-embryonic ectoderm; DVE, distal visceral endoderm. (B) Whole mount *in situ* RNA hybridization analysis of wild type and mutant E4.5 and E5.5 embryos for *Nanog, Gata6*, *Lefty1, Cer1*, *Hex1,* and *Nodal* expression. At E4.5, *Bptf* mutant embryos show expression of *Nanog, Gata6*, *Lefty1,* and *Hex1,* suggesting that the primitive endoderm and inner cell mass is present in *Bptf* mutants. Mutant E5.5 embryos are defective in the expression of DVE markers *Cer1* and *Hex1,* suggesting that mutants cannot form the DVE. Interestingly, the general visceral endoderm marker *GATA6* is not expressed in the VE but rather in the embryonic ectoderm at E5.5.

As a first step in the molecular analysis of Bptf in embryonic development we examined the expression of *Bptf* using *in situ* RNA hybridization. We observed expression in the inner cell mass (ICM) and primitive endoderm at E4.5 and all embryonic tissues at E5.5 and E6.5. Interestingly we do not observe *Bptf* expression in the visceral endoderm at E5.5 and E6.5 (Figure 2A). We also monitored the activity of the β-galactosidase moiety of the Bptf–β-Geo fusion protein in heterozygous mice. Consistent with our *in situ* analysis a histochemical analysis of whole mounts showed that *Bptf*–*β-Geo* is expressed in the embryo proper at E5.5, E6.5 and E7.5 (Figure S8A, S8B, S8C, S8D, S8E, S8F, S8G). Further analysis of histological sections revealed that *Bptf*–*β-Geo* expression at E7.5 is primarily confined to the embryonic ectoderm, with reduced levels in mesoderm and no expression in the visceral endoderm (Figure S8A′, Figure S8B′). At subsequent stages, from E7.5 to E13.5, histochemical analysis of whole mounts showed widespread *Bptf*–*β-Geo* expression in the developing embryo (Figure S9A, Figure S9B, S9C, S9D, S9E, S9F). This temporal correlation between the earliest stages of *Bptf* expression and the stages when the mutant phenotype is revealed, suggests that there could be an essential requirement for *Bptf* as early as E4.5.

Our histological analysis suggests that *Bptf* mutants are defective in establishing an A-P axis. Apparent defects in A-P axis can be due to defects in the establishment or migration of the DVE [29]. To monitor the development of the DVE and its transition to the AVE, we analyzed the markers *Otx2, Lefty1, Cer1, Hesx1, Hex1, Gata6, Nanog* and *Nodal* by *in situ* hybridization in E4.5 to E6.5 mutant embryos [32]–[37].

**Figure 3:**
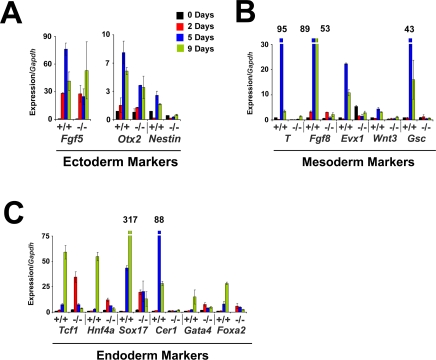
Analysis of *Bptf* knockout mouse embryonic stem cells shows severe defects in gene expression during embryoid differentiation. Relative expression of developmental markers during an embryoid body differentiation time course. The expression of many markers of the ectoderm (A), mesoderm (B), and endoderm (C) tissue lineages were severely defective in *Bptf* mutants during the differentiation time course.

An analysis of pre-implantation embryos suggested that *Bptf* mutants specify a functional primitive endoderm and ICM. The primitive endoderm of *Bptf* mutant embryos was found to express the primitive endoderm markers *Gata6, Lefty1,* and *Hex1* at comparable levels to wild type controls (Figure 3B). The expression of these markers suggests that *Bptf* mutants are not defective in the differentiation of the primitive endoderm, the precursor of the visceral endoderm of E5.5 and later stage embryos. Consistent with a functional ICM, we observed normal expression of the pluripotency marker *Nanog* in *Bptf* mutants compared to controls (Figure 2B). Taken together, these results indicate that *Bptf* is not required for the specification of the primitive endoderm and the ICM in E4.5 embryos.

To assess the specification of the VE and DVE, we monitored the expression of *Cer1, Hex1, Gata6,* and *Nodal* in E5.5 *Bptf* mutant embryos. We observed that the markers *Cer1, Hex1* are significantly reduced in *Bptf* mutants relative to the wild type controls (Figure 2B). The absence of expression of these markers indicates that the DVE does not form in the absence of *Bptf*. Interestingly *Gata6* expression, normally expressed only in the VE at E5.5 and E6.5, is absent in the VE but present in the epiblast in mutants at E5.5 (Figure 2B). This suggests that *Bptf* could also have roles in specifying the VE as well as the DVE. A key regulator of DVE specification is *Nodal*. *Nodal* expression is found as early as E4.5 but is not significantly expressed in the epiblast and VE until implantation is well underway at E5.0 [36],[38]. In E5.5 *Bptf* mutant embryos we observe normal expression levels of *Nodal* in the epiblast and VE as in wild type embryos (Figure S10A). This E5.5 pattern of *Nodal* expression continues into E6.5 in *Bptf* mutant embryos (Figure S10B).

To examine the development of the AVE we monitored the expression of *Cer1, Otx2, Hesx1, Lefty1* and *Hex1* in E6.5 *Bptf* mutant embryos. *Otx2* expression is required for the migration of the DVE to establish the AVE. We observed lower expression of *Otx2* in the epiblast of *Bptf* mutant embryos relative to there wild type controls (Figure S11A). As expected we did not observe expression of AVE markers *Cer1, Hesx1, Lefty1* and *Hex1* in *Bptf* mutant embryos (Figure S11A). Combined with our analysis of E5.5 embryos our results strongly suggest that Bptf is required for the speciation of the DVE and the AVE.

Since *Bptf* mutant embryos are unable to form a functional DVE and AVE, we anticipated that they should be defective in specifying the primitive streak and differentiating mesoderm and definitive endoderm. Several critical transcription factors and signaling molecules such as *T, Lhx1*, *Fgf8*, *Gsc*, *Foxa2, Nodal,* and *Cripto* (*Tdgf1*) serve as effective markers for development of the primitive streak in the gastrulating embryo [34], [39]–[42]. Our analyses revealed that *T, Lhx1*, *Fgf8*, *Gsc*, and *Foxa2* were undetectable at E6.5, and in the case of *T*, *Fgf8* but not *Lhx1*, were delocalized in expression at E7.5 (Figure S11A, Figure S11B). The absence of expression of primitive streak markers at E6.5 confirms our histological analyses, and further supports the observation that gastrulation and mesoderm formation do not occur in *Bptf* mutants. Interestingly, the delocalized *Nodal* and *Cripto* expression patterns observed in the *Bptf* mutants at E6.5 are highly reminiscent of their expression patterns prior to the establishment of the DVE (Figure 2B) (Figure S11A) (Figure S10A, Figure S11B) [34],[39]. Taken together, the data suggests that *Bptf* mutant embryos arrest at a stage prior to DVE formation (<E5.5).

Recent models propose that the extra-embryonic ectoderm supports the growth of the embryo and is a source of signals for A-P axis establishment [29]. To address whether the developmental defects of *Bptf* mutants are caused by defective extra-embryonic ectoderm or by a lack of appropriate growth signals in the epiblast, we monitored the expression of the extra embryonic ectoderm (*Bmp4*, *Erbb2, Fgfr2*), trophectoderm (*Mash2*), angiogenesis (*Vegf*, *Flk1*) and a cell cycle regulator (*JunB*) markers [43]–[47]. We find *Bmp4, Mash2, Erbb2,* and *Fgfr2* to be expressed normally in the mutant embryos at E6.5 and E7.5 (Figure S11A, Figure S11B). This suggests that the growth defects observed in *Bptf* embryos are not due to gross defects in the specification the extra-embryonic tissues. We also observe little to no expression of the angiogenesis markers *Vegf* and *Flk1* in the extra-embryonic tissues at E7.5 (Figure S11B). However, expression of the cell cycle regulator *JunB* is increased in mutant embryonic ectoderm when compared to wild-type controls (Figure S11A). *JunB* is a member of the Ap-1 family of transcription factors which acts as a negative regulator of the cell cycle [48]. This up-regulation of *JunB* is consistent with our observations that *Bptf* mutants have reduced cellular proliferation (Figure 1B) (Figure S7).

In summary, our analysis of lineage markers by *in situ* RNA hybridization has revealed an essential role for *Bptf* in specifying the VE and the DVE of the E5.5 post implantation embryo. These defects likely lead to the observed absence of an AVE and primitive streak in E6.5 embryos. The absence of these developmental organizers arrests the growth of the embryo prior to gastrulation and is likely a major cause of the early embryonic lethal phenotype of *Bptf* mutant embryos.

### *Bptf* Is Required for Embryonic Stem Cell Differentiation

To further explore the role of Bptf in cell differentiation we generated *Bptf* knockout mouse ES cells and examined their development *in vitro* and *in vivo*. By gene targeting and transient Cre expression we were able to generate eight independent homozygous *Bptf^ΔExon2^* knockout ES cell lines with a euploid karyotype (Figure S12A). We observed varying degrees of reduced *Bptf* transcript levels by Northern blotting in mutant cell lines compared to that of wild type controls (Figure S12B). An analysis of *Bptf^ΔExon2^* mRNA from exons 1 to 8 in wild type and knockout cell lines shows the message to be out of frame resulting in no observable protein in the knockout cell lines (Figure S12C, Figure S12D) (data not shown).

To address the possibility the Bptf is essential for cell viability and proliferation we measured the doubling time of knockout *Bptf* ES and MEF cell lines (Figure S13). Both the *Bptf* knockout ES and MEF cell lines were viable but exhibited slightly reduced cellular proliferation (Figure S14). These results show that *Bptf* is not required for cell viability and is only marginally required for cellular proliferation.

We measured the differentiation potential of the *Bptf* knockout ES cells *in vitro* as embryoid bodies and *in vivo* as teratomas. *Bptf* wild type and two independent knockout cell lines were subcutaneously injected into NOD/SCID mice and allowed to form teratomas over 8 weeks. Wild type ES cells formed teratomas in 8/11 injected mice. *Bptf* knockout lines did not form any observable tumors in 10 injections (data not shown). This study demonstrated that *Bptf* is essential for one or more biological processes including cell viability, proliferation or differentiation in the animal.

We next utilized an embryoid body analysis to monitor differentiation in *Bptf* knockout cell lines to ectoderm, mesoderm and endoderm cell lineages. Analysis of the mutant embryoid bodies showed little evidence of apoptosis by TUNEL, and similar densities of PCNA and phosphorylated histone H3 positive cells (Figure S15A). We did observe that *Bptf* knockout embryoid bodies were slightly smaller in size and did not form any observable endoderm (Figure S15B, Figure S15C). These results indicate that *Bptf* is not necessary for cellular survival or proliferation under conditions of embryoid body differentiation but is essential for differentiation of endoderm and possibly other advanced tissue lineages.

To investigate the differentiation defects of *Bptf* knockout embryoid bodies at a molecular level we monitored the transcription of well documented markers of endoderm, mesoderm and ectoderm differentiation (Figure 3A–3C). We observed minimal *Bptf* dependence for the primitive ectoderm markers *FGF5* and *Otx2* (Figure 3A). However, we did observe significant defects in *Nestin* transcription, a marker of neural stem cell progenitors derived from the primitive ectoderm (Figure 3A). These results indicate that primitive ectoderm lineages are less dependent on *Bptf* than the more differentiated lineages. As anticipated we observed severe defects in the expression of mesoderm and endoderm markers during the differentiation time course. We observed little to no activation of mesoderm markers *T*, *FGF8, Evx1, Wnt3*, and *Gsc* which are significantly activated in wild type cultures by day 5. (Figure 3B). Similarly, endoderm markers *Sox17*, *Cer1, Hnf4a,* and *Foxa2* show severe expression defects in *Bptf* mutant embryoid bodies compared to controls (Figure 3C). In contrast to the large differences in expression of mesoderm and endoderm markers we observed less than two fold changes in expression of cell cycle regulators and pluripotency markers with the exception of *Cyclin D1* (Figure S16A, Figure S16B). These defects in transcription and differentiation were rescued for two independent knockout lines by retargeting exon 2 to the *Bptf^ΔExon2^* locus using the targeting vector. Our ability to rescue the expression defects of *T, Gsc, Sox17* and *Cer* suggest that the observed phenotypes are due to *Bptf* mutation (Figure S17A, Figure S17B, Figure S17C) (data not shown). Taken together these results suggest that *Bptf* is essential for the formation of mesoderm, endoderm and more differentiated ectoderm lineages in the embryoid body.

**Figure 4:**
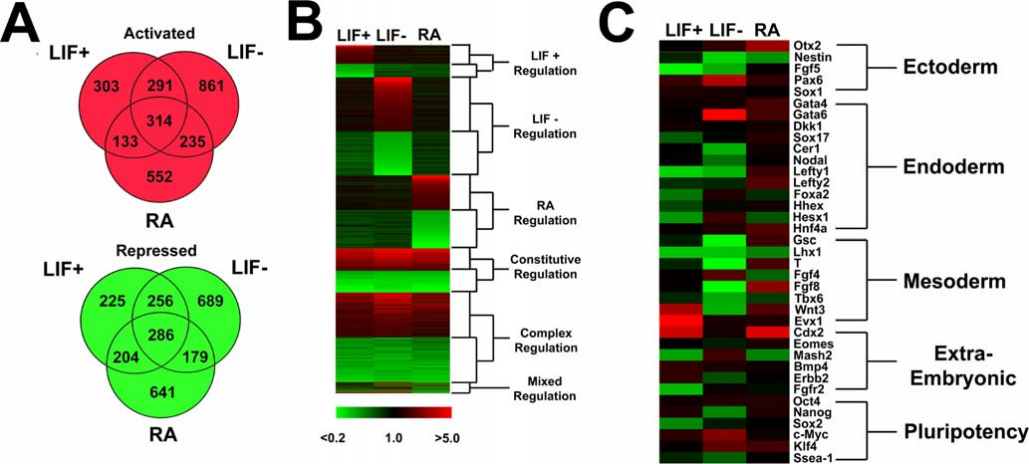
Analysis of *Bptf* knockout mouse embryonic stem cells shows severe defects in gene expression during differentiation with RA and LIF withdrawal. *Bptf* knockout embryonic stem (ES) cells were induced to differentiate using LIF withdrawal (LIF−) or the addition of retinoic acid (RA), and gene expression was monitored by microarray. (A) *Bptf*-dependent genes were defined as genes whose transcription increases or decreases more than 2-fold in the mutant compared to the wild type with a FDR value of <0.05. (B) A manual clustering analysis of *Bptf*-dependent genes by condition of dependence and expression values. Six expression categories were identified and include genes which are exclusively *Bptf*-dependent in LIF−, LIF+, or RA conditions, genes which are dependent in all conditions (constitutive regulation), genes which are dependent in 2 conditions (complex regulation), or genes which vary in direction of misregulation between conditions (mixed regulation). (C) Clustering analysis of *Bptf-*dependent genes important for the development of tissue lineages of the early embryo. *Bptf* is essential for the proper regulation of many markers of ectoderm, endoderm, and mesoderm tissue lineages.

We chose a microarray approach to investigate any differentiation defects of *Bptf* knockout ES cells in undifferentiated and early differentiation states. Differentiation was induced by LIF withdrawal (LIF−) or retinoic acid (RA) for three days before harvesting RNA. A comparison of *Bptf*-dependent genes by Venn diagram and manual clustering identifies six categories; those being affected under only one of the growth conditions (LIF+, LIF− or RA Regulation), those being affected in all three conditions (Constitutive Regulation), a class of genes which were regulated in the same direction in two of three conditions (Complex Regulation) and genes with mixed dependence (Mixed Regulation) (Figure 4A and 4B) (Dataset S1).

From our microarray analysis we observed large changes in gene expression under conditions of maintained pluripotency (LIF+) and particularly under conditions of differentiation (LIF− and RA) (Figure 4A and 4B). As expected many of the essential markers of early embryonic tissue differentiation that are dependent on *Bptf* in embryos are also *Bptf*-dependent in ES cells (Figure 4C). These markers include; pluripotency regulators *Sox2, c-Myc, Nanog,* visceral endoderm markers *Lefty1*, *Cer1*, *Hex1*, *Foxa2* and the primitive streak markers *Gsc*, *Lhx1*, *Wnt3*, *Fgf8*, *T* (Figure 4C). These results reinforce the view that *Bptf* regulates the development of ectoderm, endoderm and mesoderm in both the early embryo and in ES cells.

A gene ontology (GO) analysis of *Bptf*-dependent expression datasets revealed an over representation of genes with “transcription factor activity”, genes involved in the biological processes of “development” and “morphogenesis” and the cellular processes of “cell death” and “cell proliferation” (Figure S18). Notable gene clusters include the consistent activation of genes correlated with “nervous system development” in all datasets, the repression of MHC I and II receptors during LIF−differentiation, the activation of genes correlated with “cytoskeletal components” during RA differentiation (Figure S18). In addition, we observed a striking over-representation of homeobox transcription factors within the “transcription factor activity” annotation. The homeobox-containing genes were almost exclusively up-regulated in each of the expression categories, and in some cases include almost the entire Hox gene cluster, indicating that *Bptf* is required for their repression in ES cells (Figure S19A, Figure S19B).

From our analysis we also observed that *Bptf*-dependent gene targets are more likely to be actively regulated genes, repressed in the presence of LIF or RA differentiation and conversely activated under conditions of LIF differentiation (Figure S20A, S20B, S20C). In support with these observations we observed histone modifications in *Bptf* knockout ES cell lines consistent with a repressed transcriptome under LIF+ growth conditions (Figure S20D). Interestingly some *Bptf*-dependent genes cluster together in the genome (Figure S21).

### A Role for Bptf in Distal Visceral Endoderm Formation

A diagnostic defect of *Bptf* embryos is an inability to form the DVE. In the absence of the DVE, the AVE cannot form preventing the necessary signals for the specification of the primitive streak. To further investigate the functions of NURF in the early embryo we focused on Smad mediated signaling pathways. Smad mediated signaling pathways are essential for the formation of the DVE in the embryo and the induction of mesoderm in differentiating ES cells, two prominent phenotypes of our *in vivo* and *in vitro* studies on *Bptf* [49],[50].

The most prominent ligand activating the Smad transcription factors in the early embryo is Nodal [51]. Nodal, and the closely related ligand activin, bind to type I and II TGFβ receptors resulting in the phosphorylation of the type I receptor. Phosphorylation of the type I receptor activates a kinase domain which phosphorylates Smads2/3. The phosphorylation of Smad2 or Smad3 transcription factors promotes interactions with Smad4 and triggers the translocation of the Smad complex into the nucleus [51]. Once in the nucleus, the Smad complex interacts with DNA sequence specific transcription factors to promote the regulation of Smad target genes [52].

**Figure 5:**
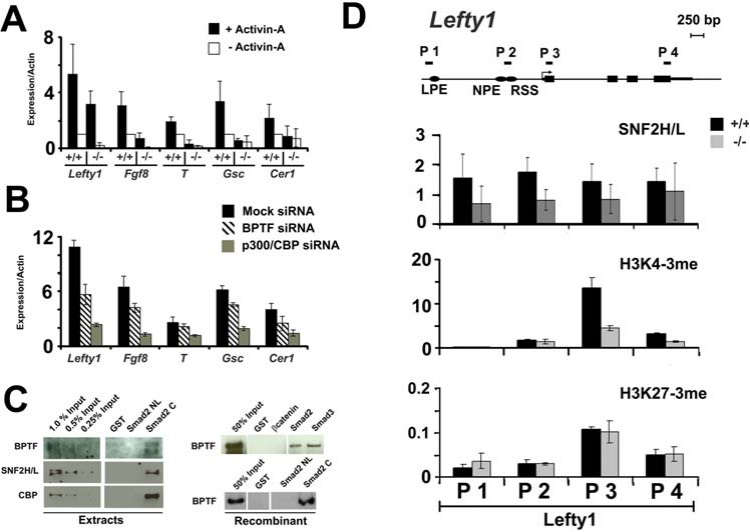
*Bptf* is necessary for Smad mediated gene regulation. (A) RT-PCR analysis of known Smad target genes from activin-A induced wild type (+/+) and *Bptf* knockout (−/−) embryonic stem (ES) cells shows *Cer1, T*, *Gsc* to be dependent, and *Fgf8*, *Lefty1* to be partially dependent on *Bptf* for full activation. (B) Like CBP/p300, Bptf regulates Smad-dependent genes *in vivo*. Bptf or CBP/p300 were knocked down in activin-A induced or uninduced ES cells. The expression of gene targets was determined by RT-PCR and is expressed as a ratio of induced/uninduced for each knockdown condition. *Lefty1, Fgf8, T, Gsc,* and *Cer1* require both Bptf and CBP/p300 for full activin-A–dependent gene activation. (C) NURF interacts with the Smad transcription factors *in vitro*. Recombinant NURF complex was subjected to GST pull-down assays using resin bound GST, GST-Smads, GST-Smad fragments, and GST-β-catenin. GST-Smad2 and 3 but not GST and GST-β-catenin controls can specifically pull down the NURF complex. The GST-Smad2C containing the C-terminal fragment of Smad2 specifically pulls down the NURF complex. The C-terminal domain of Smad2 interacts with native NURF from crude nuclei extracts. High salt nuclei extracts from ES cells was subjected to GST pull-down assays using resin bound GST and GST-Smad fragments. The GST-Smad2C containing the C-terminal fragment of Smad2 specifically pulls down the Bptf and Snf2h/l components of the native NURF complex and the histone acetyl-transferase CBP from nuclei extracts. (D) Chromatin immunoprecipation of Snf2h/l and histone modifications 3me-K4H3 and 3me-K27H3 at *Lefty1* show recruitment of the Snf2h/l subunit to the neural plate specific enhancer (NPE), a region which contains Smad binding elements, in a Bptf and an activin-A–dependent manner. Snf2h/l ChIP is expressed as a ratio of induced (+activin-A) to uninduced (−activin A). Histone modifications are shown during induced (+activin-A) conditions for +/+ and −/− cells and have been normalized to a pan histone H3 pulldown. NPE = neural plate specific enhancer, LPE = lateral plate specific enhancer, RSS = right side specific enhancer.

Accordingly, we monitored the dependence of Smad-responsive genes on the presence of *Bptf* in ES cells. ES cells readily responded to activin-A as monitored by the phosphorylation of Smad2 (Figure S22A, Figure S22B). From these experiments we identified a number of Smad-dependent genes which completely or partially require *Bptf* for full activation. Genes requiring *Bptf* for full activation include *Cer1, Gsc* and *T* (Figure 5A). Genes which partially require *Bptf* include *FGF8*, *Lefty1* and *p21* (Figure 5A) (Figure S23).

To understand the relationship between Bptf and CBP/p300, known co-activators of the Smad transcription factors, we knocked down both Bptf and CBP/p300 using siRNA technology and monitored the activation of the Smad responsive genes *Lefty1, FGF8, Gsc, T,* and *Cer* in ES cells (Figure S22C). As in the *Bptf^ΔExon2^* knockout ES cell lines we observed that each of these genes are dependent on *Bptf* for full activation (Figure 5B). While some genes differ in there requirement for Bptf, they are all dependent on CBP/p300 for activation (Figure 5B). Our results demonstrate that a genetic knockout and siRNA mediated knockdown of Bptf result in defects in the activation of Smad responsive genes to varying degrees. Taken together, these results indicate that Bptf, like CBP/p300, acts as a co-activator of Smad responsive genes in ES cells.

We also used the embryonic carcinoma cell line P19 to complement our findings with the *Bptf* knockout ES cells. We co-transfected P19 cells with DNA plasmids carrying four minimal Smad binding elements (SBE), or three activin response elements (ARE), linked to a core promoter and a luciferase reporter gene. The ARE and SBE elements were previously found to be the minimal Smad-responsive elements [53],[54]. Under conditions of *Bptf* knockdown, we observed a significant reduction in luciferase activity from both reporters (Figure S24A, Figure S24B).

To simulate Smad signaling in a different way we co-transfected combinations of Smads 2,4 with constitutively active TβRI (ALK5), the type I receptor for the TGFβ signaling pathway (Figure S24C) [55]. Using this system we observed efficient reduction in the activation of the SBE regulated luciferase reporter gene with a siRNA to *Bptf* but not a mock siRNA control (Figure S24D). We repeated the experiments using multiple unique siRNAs and measured the transcription of endogenous TGFβ regulated genes. In these experiments we used three individual *Bptf* siRNAs which were effective in knocking down protein expression after 2 days of culture (Figure S24E). We then stimulated the P19 cells with TGF-β1. Like nodal and activin-A, TGF-β1 stimulates the phosphorylation of Smad 2/3 through the dimerization and activation of similar type I and II receptors. We similarly observed a significant reduction of *Cer1* and *T* transcription in Bptf depleted cells upon Smad2/3 activation with TGF-β1 (Figure S24F). We also used the human breast cancer cell line MCF10CA1 in similar assays to determine if BPTF could play a role in Smad signaling in humans [56]. In these experiments we used *BPTF* siRNAs which were effective in knocking down protein expression in MCF10CA1 cells after 2 days of culture (Figure S24G). We then stimulated the MCF10CA1 cells with TGF-β1 for 1 hour. We observed a significant reduction of PAI-1 but not SMAD7 induction suggesting that, as in the mouse, BPTF could regulate a subset of Smad responsive genes in humans (Figure S24H).

We next investigated whether the interaction between the BPTF-containing NURF complex and the Smads is direct or indirect using pull-down assays. Experiments with bacterially expressed GST-Smad2 or GST-Smad 3 showed an interaction to a degree between recombinant NURF complex and each of the Smad transcription factors, but not the GST or GST-βcatenin controls (Figure 5C). Smad transcription factors are composed of functional domains at the N-terminus (MH1 domain) and C-terminus (MH2 domain). The N-terminal+linker and C-terminal regions of Smad2 were used in similar pulldown experiments. We observed that recombinant NURF complex interacts specifically with the MH2 domain of Smad2 (Figure 5C). This interaction was also observed for the Bptf and Snf2l components of native NURF complex from crude ES cell nuclear extracts (Figure 5C). We also confirmed the reported interaction of the C-terminal MH2 domain of the R-Smads with the co-regulator CBP (Figure 5C) [57]. Hence, our results suggest that NURF, like p300 and CBP maybe recruited to the promoters of TGFβ responsive genes through direct interactions with the Smad transcription factors.

Our current model proposes that Bptf-containing complexes like NURF are recruited to the promoter of Smad regulated genes through direct interactions with the Smad transcription factors. To test this model, we initiated chromatin immuno-precipitation (ChIP) experiments to detect the Snf2l component of the NURF complex at *Lefty1*. As anticipated, we observed significant enrichment of Snf2l at the Neural Plate Specific Enhancer (NPE), a region which contains putative FAST and Smad transcription actor binding sites, of the *Lefty1* promoter [58]. This enrichment was dependent on activin-A stimulation and on the presence of Bptf (Figure 5D). Snf2l enrichment correlates with the presence of the activating histone modification H3K4me3 at the 5′ UTR of *Lefty1* (Figure 5D). These results are consistent with Bptf recruitment to the promoter of *Lefty1* through the Smad transcription factors.

### Discussion

In this work we report a post-implantation lethal phenotype for mutations in *Bptf*, the previously characterized largest subunit of the NURF chromatin remodeling complex [15]. In the early embryo *Bptf* is expressed by E4.5 in the ICM and primitive endoderm. At later stages of development *Bptf* is expressed in both embryonic and extra-embryonic ectoderm by E5.5. Following gastrulation, *Bptf* is widely expressed in all germ layers to E13.5. *Bptf* expression is essential for early development because homozygous *Bptf* mutant embryos are reabsorbed by E8.5. No overt defects were observed in the mutant E3.5 or E4.5 embryo or its ability to proliferate in culture, suggesting that *Bptf* embryos undergo a normal initial specification and proliferation of the ICM, primitive endoderm and trophectoderm. However, mutant embryos exhibit diminished proliferation post-implantation as shown by defects in size of both extra-embryonic and embryonic tissues and decreased phosphorylated histone H3 staining. A histological analysis of mutant embryos at E6.5 and E7.5 revealed that they develop a VE but do not form a primitive streak or differentiate mesoderm. To investigate the causes of the defect in gastrulation, we monitored the expression of key markers prior to and during gastrulation in the embryo. As anticipated we failed to observe expression of primitive streak markers *T, Foxa2, Gsc, Fgf8* or the posterior localization of *Nodal* and *Cripto* expression. Defects in the expression of primitive streak markers and the delocalized expression of *Nodal* and *Cripto* are likely due to the absence of the DVE/AVE. Defects in the DVE/AVE were confirmed by observing significantly reduced expression of the markers *Cer1, Hex1, Lefty1* and *Hesx1* in E5.5 and E6.5 embryos. Defects in *Gata6* expression at E5.5 suggest that the defects in DVE specification are accompanied by general defects in VE specification. From these studies we conclude that a critical function for *Bptf* during mammalian development is directly or indirectly to specify the VE and DVE after implantation.

To identify Bptf-dependent gene targets we employed a microarray based approach on *Bptf* knockout ES cells during the early stages of differentiation and an embryoid body model. We discovered a role for *Bptf* in the regulation of gene clusters essential for development, morphogenesis, nervous system development and cell death and proliferation. Interestingly many transcription factors, primarily the homeobox-containing genes, are dependent on *Bptf* for their proper repression during undifferentiated and differentiated states. This dependence is interesting as NURF has been shown to be a activator of Hox gene transcription in more differentiated tissues in Drosophila and the mouse [13],[15]. As expected many markers of ectoderm (*Nestin, Fgf5*), mesoderm (*Gsc, Lhx1, Fgf8, Tbx6, Wnt3*) and endoderm (*Lefty1, Cer1, Nodal, Hesx1*) cell lineages require *Bptf* for their expression. These gene targets corroborate well with those identified from our *in vivo* studies on *Bptf* mutant embryos further supporting the conclusion that *Bptf* is essential for the development of ectoderm, mesoderm and endoderm. This also suggests that our microarray dataset obtained from ES cells is a reasonable approximation of the expression defects occurring in *Bptf* mutant embryos *in vivo*.

### A Model for Bptf Function in Early Mammalian Development

The functions for Bptf in the early embryo are undoubtedly complex. Our unbiased analysis of gene targets by microarray revealed many potentially Bptf-dependent differentiation pathways. Most relevant to this study include the regulation of the cell cycle and the differentiation of mesoderm and endoderm lineages, specifically the VE and DVE. The underlying cause for these defects could largely be due to the loss of chromatin associated complexes like the NURF chromatin remodeling complex. In the case of NURF the defects could be direct, as a remodeling activity at the promoter of genes necessary for cellular proliferation and differentiation, or indirect through the deregulation of master regulators of development like the homeobox-containing transcription factors. Moreover the underlying mechanism of Bptf action as a co-activator of some genes and a co-repressor of others is unclear. Further studies of nucleosome positioning and chromatin structure in mutants should clarify these possibilities.

In addition to system-wide defects with *Bptf* deletion, there could be specific defects in individual signal transduction pathways within the embryo or in the ability of the embryo to receive growth signals from the extra-embryonic tissues or the surrounding decidua. To identify potential Bptf-dependent signaling pathways we focused on its role in specifying the DVE. The pre-gastrulation embryo uses three well-known signaling pathways, the WNT/β-catenin, FGF/MAPK and Nodal/Smad pathways, to establish A-P asymmetry [29]. The three pathways can be distinguished by different A-P phenotypes. In mutants of FGF/MAPK signaling the epiblast has severe proliferation defects, do not specify primitive endoderm, are quickly reabsorbed and the blastocysts do not outgrow when grown in culture [30],[44],[59],[60]. Mutations in the WNT/β-catenin, but not Nodal/Smad pathways, develop the DVE and in some cases the AVE [50], [61]–[63]. The ability of the *Bptf* embryos to form blastocyst outgrowths, specify the primitive endoderm, but not form the DVE is reminiscent of mutants in the Nodal/Smad signaling pathway rather than a defect in FGF/MAPK or WNT/β-catenin signaling (Table S2).

Because Bptf has been associated with the NURF complex, a known regulator of transcription, the data suggests that *Bptf* is required for the expression of gene targets in the developing VE and DVE. In support of this hypothesis, we showed that Bptf is required for the regulation of endogenous promoters and Smad responsive promoter elements in ES, P19 and MCF10CA1 cells in tissue culture. Smad-dependent gene targets include those essential for cell proliferation (*p21*) and those essential for DVE function (*Cer1, Lefty1*) and primitive streak (*T, Fgf8, Gsc*). Moreover, pulldown assays showed that components of the NURF complex have direct interactions with the Smad transcription factors and it is recruited to the promoters of Smad regulated genes under conditions of activation. Taken together, our data suggest that Bptf can directly regulate Smad regulated genes, likely through the functions of the NURF remodeling complex, via recruitment by the Smad transcription factors (It is also possible that other as yet unidentified Bptf-containing complexes distinct from NURF function in this pathway). To address the possibility that the effects on the Nodal/Smad pathway are indirect we monitored the expression of key components of the pathway in embryos. We did not observe significant changes which could explain the observed defects in Smad signaling in the early embryo or during embryoid body differentiation in *Bptf* mutants (Figure S25).

Consistent with these findings, the ISWI ATPase, a component of Drosophila NURF, ACF and CHRAC has been reported to be important for transmitting Dpp/TGFβ signals to stem cells in the *Drosophila* ovary [64]. However, we wish to emphasize that the *Bptf* mutation likely affects many different pathways and biological processes, each of which may contribute to biological phenotypes. The challenge for the near future will be to uncover each of the many functions of Bptf in mammalian chromatin biology.

## Materials and Methods

### Targeting Vector Construction

The RPCI21 mouse PAC library (MRC Genomic Resource Center, England) was screened using a random hexamer labeled probe to *Bptf* exon 2. Blotting was performed in hybridization bags using 0.25 M sodium phosphate pH 7.2, 1 mM EDTA, 7% SDS, at 65°C overnight with rocking. Blots were washed 5 times for 10 min with 0.25× SSC, 0.1% SDS at 65°C. Eight positive clones were identified using X-ray film as; 367-I21, 373-D5, 402-C16, 402-E15, 426-P15, 540-B9, 625-D23, 582-P16. Clone 367-I21 was confirmed by Southern blotting using Eco RI and Sal I digests and *Bptf* exon2 probe. Genomic sequence for the construction of the targeting vector was retrieved into bluescript SKII (Stratagene) and the integration of loxP sites and *Neo* selectable marker was performed using recombineering technology described previously [65]. The sequence of the targeting vector is available upon request.

### Targeting *Bptf* in ES Cells

Linearized vector was electroporated into CJ7 ES cells and 71 individual Neo resistant and HAT resistant clones were isolated according to previously published procedures [66]. Clones were screened for successful *Bptf* targeting using Eco RI, probe 29–31; Bam HI, probe exon2; and Sca I, probe exon2. 16 of 71 isolates were found to be correct for a recombination frequency of 22%.

To create heterozygous, conditional homozygous, homozygous knockout and rescue *Bptf* ES cell lines we transiently expressed Cre followed by retargeting. The piCre expression vector was electroporated into clone 7010 and conversion from *Bptf/Bptf^FloxedNeo^* to *Bptf/Bptf*Δ*^exon2^* was first screened for by PCR then confirmed by Southern blotting. Three clones were identified using this strategy, and clone 7004 was used for subsequent targeting. The wild type allele in the *Bptf/Bptf*Δ*^exon2^* from clone 7004 was then retargeted using the *Bptf* exon 2 targeting vector. Individual conditional homozygous clones were first screened for by PCR then confirmed by Southern blotting. Two *Bptf^FloxedNeo^/Bptf*Δ*^exon2^* clones were obtained and named H12 and B19. The piCre expression vector was electroporated into H12 and B19 and conversion from *Bptf^FloxedNeo^*/*Bptf*Δ*^exon2^* to *Bptf*Δ*^exon2^/Bptf*Δ*^exon2^* was first screened for by PCR then confirmed by Southern blotting. A total of 9 and 5 homozygous knockout clones were obtained from the H12 and B19 parental lines respectively. The karyotype of the knockout lines were confirmed using Giemsa staining and lines with an anuploid karyotype were discarded. ES cells were maintained on mitomycin C treated primary mouse embryonic fibroblasts (MEFs) and ES Cell Growth Media (15% FCS (ES Cell grade, Invitrogen), DMEM, essential amino acids,10 µM mercapto-ethanol, 2 mM glutamine, 100 U/ml LIF (Chemicon International), penicillin and streptomycin) throughout. Lines were passaged off MEFs onto gelatinized plates for 3 passages prior to any molecular analysis.

Rescue lines were made by retargeting one allele of *Bptf*Δ*^exon2^* in clones *Bptf* mutant clones P2-G2 and P2-B9. Individual Neo resistant and HAT resistant clones were selected on mitomycin-C treated MEF feeder layers in ES Cell Growth Media (15% FCS (ES Cell grade, Invitrogen), DMEM, essential amino acids,10 µM mercapto-ethanol, 2 mM glutamine, 100 U/ml LIF (Chemicon International), penicillin and streptomycin) throughout. Successful retargeting events were confirmed by Southern analysis as described below.

### Production of Chimera Mice and Animal Husbandry

Clone 7010 was used to make chimera mice as described previously [66]. Removal of frt-Neo-frt and deletion of LoxP-exon2-LoxP was accomplished by crossing to mice expressing Tg-CMV-Flp and Tg-CMV-Cre to *Bptf^FloxedNeo^* to create the *Bptf^Floxed^* and *Bptf*Δ*^exon2^* lines respectively. Individual recombinants were identified by Southern blotting and maintained by backcrossing to C57B6/CRL.

Mice used in this study were maintained in a Specific Pathogen Free environment at the National Institutes of Health at Bethesda (MD, USA). Mice were maintained on a 12 hr light/dark cycle given NIH-13 blend lab chow and hypo chlorinated water *ad libtium* throughout the duration of the study. All experiments and animal maintenance procedures were approved by the Animal Care and Use Committee of the National Cancer Institute under protocol LMCB001 and its modifications.

NCI-Frederick is accredited by AAALAC International and follows the Public Health Service Policy for the Care and Use of Laboratory Animals. Animal care was provided in accordance with the procedures outlined in the Guide for Care and Use of Laboratory Animals” (National Research Council; 1996; National Academy Press; Washington, D.C.).

*Bptf^XG023^*, *Bptf^FloxedNeo^*, *Bptf^Floxed^*, and *Bptf*Δ*^exon2^* embryos on a B6/129 mixed background have been cryopreserved at the Cryopreservation and Assisted Reproduction Lab, National Cancer Institute, Frederick (MD, USA) as BPTFXG023, BPTFFloxedNEO, BPTFFloxed and BPTFdel-exon2 respectively. These mouse lines are available to the research community by request.

### Southern Blotting Genotyping Procedures

Mice were routinely genotyped by Southern blotting restriction digested tail DNA. Tail DNA was prepared using standard procedures [67]. ∼10 µg of DNA was digested with the appropriate restriction enzyme and resolved by 0.5% agarose gel electrophoresis for standard electrophoresis or 1.0% agarose using FIGE. DNA was transferred to Hybond XL (Amersham) using alkaline capillary transfer crosslinked with UV (Stratalinker) and probed with random hexamer labeled probes at 65°C using Perfect Hyb Hybridization Buffer (Sigma). Probes used for blotting were PCR amplified from pCH110 (Genbank # UO2445) using primers JL510 and JL511 for Probe B, and from mouse genomic DNA using primers JL299 and JL300 for Probe A; JL60 and JL61 for probe exon2; JL235 and JL236 for probe 29–31; JL293 and JL294 for probe 25–26; JL295 and JL296 for probe 20.2–20.7 using Taq polymerase (Invitrogen) (see Table S3 for primer sequences). Blots were washed twice with 2.0× SSC, 0.1% SDS and twice with 0.5× SSC, 0.1% SDS for 10 min @ 65°C for each wash. A Phosphoimager was used to detect the hybridization signal.

### PCR Genotyping Procedures

Embryos were genotyped using a PCR based strategy. Primers used for genotyping the *Bptf^XG023^* line were as follows, Primer A (JL511), Primer B (JL627), and Primer C (JL631) (see Table S3 for primer sequences). PCR reactions were performed in 25 µl volumes with 20 mM Tris-HCl (pH 8.4), 50 mM KCl, 1.5 mM MgCl_2_, 0.2 mM each dNTP, 0.2 µM each primer, 1 Unit Taq Polymerase (Invitrogen). Reactions were heated for 3 min at 94°C, and then cycled for 35 cycles at 30 sec at 94°C, 30 sec at 65°C, and 45 sec at 72°C, followed by one cycle for 2 min at 72°C. PCR products were resolved on 1.5% agarose gels. The genotype of embryos was confirmed by the presence of a 250 bp *Bptf* and/or 500 bp *Bptf^XG023^* bands.

Two different PCR strategies were used for genotyping the *Bptf^Δexon2^* line. The primers used in the first strategy are as follows, Primer A (JL648), Primer B (JL649), and Primer C (JL652) (see Table S3 for primer sequences). PCR reactions were performed in 25 µl volumes with 20 mM Tris-HCL (pH 8.4), 50 mM KCl, 1.5 mM MgCl_2_, 0.2 mM each dNTP, 0.2 µM each primer, 1 Unit Taq Polymerase (Invitrogen). Reactions were heated for 3 min at 94°C, then cycled for 35 cycles at 30 sec at 94°C, 30 sec at 60°C, and 45 sec at 72°C, followed by one cycle for 2 min at 72°C. PCR products were resolved on 1.5% agarose gels. The genotype of embryos was confirmed by the presence of a 250 bp *Bptf*, 325 bp *Bptf^Floxed^* and *Bptf^FloxedNeo^* or 550 bp *Bptf*Δ*^exon2^* band.

The primers used in the second method are as follows, Primer C (JL651), Primer E (JL655), and Primer F (JL662) (see Table S3 for primer sequences). PCR reactions were performed in 25 µl volumes with 20 mM Tris-HCL (pH 8.4), 50 mM KCl, 1.5 mM MgCl_2_, 0.2 mM each dNTP, 0.2 µM each primer, 1 Unit Taq Polymerase (Invitrogen). Reactions were heated for 3 min at 94°C, then cycled for 35 cycles at 30 sec at 94°C, 30 sec at 65°C, and 45 sec at 72°C, followed by one cycle for 2 min at 72°C. PCR products were resolved on 1.5% agarose gels. The genotype of embryos was confirmed by the presence of a 375 bp *Bptf*, 475 bp *Bptf^Floxed^* or 650 bp *Bptf*Δ*^exon2^* bands.

To confirm *Bptf^XG023^* and *Bptf*Δ*^exon2^* expression and an out of frame *Bptf* mRNA in the *Bptf^Δexon2^* line we designed primers with homology to the 3′ end and exons 1 through 8 of the *Bptf* mRNA. Total RNA from wild type and *Bptf^Δexon2^* homozygous embryos was converted to cDNA using superscript II according to manufacturer's procedures. *Bptf* expression was estimated by first normalizing cDNA to equal concentration with *GAPDH* amplification. Following *GAPDH* normalization 3′*Bptf* was amplified. Both *GAPDH* and *Bptf* were amplified in the linear range as follows. Reactions were heated for 10 min at 94°C, then cycled for 22 cycles for *GAPDH* and 30 cycles for *Bptf* at 20 sec at 94°C, 20 sec at 60°C, and 30 sec at 72°C. PCR products were resolved by native PAGE. The exon 1–8 junction was amplified using primers P3 and JL15 in 25 µl volumes with 20 mM Tris-HCL (pH 8.4), 50 mM KCl, 1.5 mM MgCl_2_, 0.2 mM each dNTP, 0.2 µM each primer, 1 Unit Taq Polymerase (Invitrogen) (see Table S3 for primer sequences). Reactions were heated for 3 min at 94°C, then cycled for 35 cycles at 30 sec at 94°C, 30 sec at 65°C, and 2 min at 72°C, followed by one cycle for 5 min at 72°C. PCR products were resolved on 1.5% agarose gels. PCR fragments were cloned using TOPO TA cloning system (Invitrogen) and sequenced with Big Dye V3.1 using M13 forward and reverse primers.

The exact integration site for the *Bptf^XG023^* insertion allele was confirmed by sequencing a PCR product generated by amplifying genomic DNA from a heterozygous mouse tail clipping. The DNA was amplified using primers JL511 and JL515 (see Table S3 for primer sequences). Reactions were heated for 3 min at 94°C, then cycled for 35 cycles at 30 sec at 94°C, 30 sec at 65°C, and 60 sec at 72°C, followed by one cycle for 2 min at 72°C. A ∼1.8 Kb PCR product was resolved on 1.5% agarose and purified. The product was sequenced using Big Dye v3.1 using primer JL511 to obtain *Bptf^XG023^* junction sequence.

*Bptf^XG023^* disruption was confirmed by 5′ RACE according to established procedures from *Bptf/Bptf^XG023^* total testis RNA according to procedures established by the Bay Genomics Gene Trap Consortium (http://baygenomics.ucsf.edu/). Two separate 5′ RACE reactions were sequenced giving identical results.

### Embryo Procedures

Timed pregnancies used to collect embryos were preformed using standard procedures [67]. Embryonic day 0.5 was recorded the morning after an observable plug was found. Portions of later stage E18.5 to E10.5 embryos were genotyped by Southern blotting and early E9.5 to E3.5 embryos and outgrowths were genotyped by PCR.

Embryos used for β-galactosidase staining, *in situ* hybridizations and phenotypic analysis were dissected from their deciduas in PBS at room temperature prior to staining/hybridization. Embryos were stained for β-galactosidase activity or used for *in situ* hybridizations using established protocols [67] (T. Yamaguchi, personal communication). Probes used for *in situ* RNA hybridizations were labeled with DIG according to standard procedures [67]. Plasmids or PCR products used for probe synthesis were as follows *T*, *Lhx1, GATA6, Lefty1*, *FGF4*, *FGF8*, *HesX1*, *Hhex*, *Cripto*, *BMP4*, *Otx2*, *Nodal*, *Cer*, *Foxa2, Erb2, Fgfr2, Mash1, JunB, VEGF* and *Flk1*. *In situ* signals were detected using BM Purple AP Substrate according to manufactures instructions (Roche). Embryos selected for sectioning were post fixed in 4% PFA overnight at 4°C, dehydrated in alcohol and embedded in paraffin and sectioned after β-galactosidase staining. Sectioned embryos were obtained using standard techniques [67]. Embryos genotyped following β-galactosidase staining or *in situ* hybridization were prepared post staining by digesting the embryo proper overnight at 55°C in 10 to 20 µl PBS, 0.1% tween 20, 2 mg/ml PCR grade protenase K (Roche). Proteinase K was heat inactivated at 100°C for 15 min and 2 µl was subsequently used for PCR genotyping.

Deciduas used for sectioning were dissected from the uterus in PBS, washed once with PBS and immersed in 4% PFA buffered with PBS overnight at 4°C. Deciduas were then dehydrated in an ethanol series and imbedded in paraffin. Paraffin blocks were trimmed and sectioned and sections were stained with hematoxylin and eosin, TUNEL assay and phosphorylated H3 according to standard procedures [67].

Embryos used for RT PCR analysis were dissected in DMEM, 25 mM HEPES pH 7.4, 15% FCS, 2 mM glutamine, penicillin and streptomycin. Embryonic and extra embryonic tissue was removed from the ectoplacental cone and frozen in dry ice. The ectoplacental cone was grown *ex vivo* in DMEM, 15% FCS, glutamine, penicillin and streptomycin using standard culture conditions. After 4 days growth extra embryonic cells were removed from the outgrowth and subjected to PCR genotyping as described above and qPCR as described below.

Outgrowth assays were preformed on E3.5 blastocysts. E3.5 blastocysts were isolated in 15% FCS, M2, 10 µM mercapto-ethanol, penicillin and streptomycin using standard procedures. Blastocysts were then transferred to gelatinized plates containing 15% FCS, M16, 10 µM β-mercaptoethanol, 2 mM glutamine with penicillin and streptomycin. Outgrowths were cultured under standard conditions for 5 days and observed for defects. At the end of the experiment the outgrowths were removed and genotyped by PCR using conditions above.

### Tissue Extraction

Mouse tissues were extracted from 3 month old male mice sacrificed by CO2 asphixation and immediately frozen in liquid nitrogen. Tissues were then extracted using TriReagent (Sigma) according to manufacturers established procedures. RNA precipitate was extensively washed in 80% EtOH, re-suspended in DEPC treated water and stored at −80°C. Protein precipitate was extensively washed with 0.3 M guanadininum hydrochloride, 95% ethanol and re-suspended in 8M urea, 1% SDS and stored at −80°C. Protein concentration was determined using the DC Protein Assay (BioRad) according to manufacturer's procedures.

mRNA from embryos successfully genotyped as WT or *Bptf*−/− from ectoplacental growth outgrowths was isolated using Quick Prep Micro mRNA Purification Kit (Amersham).

### Northern Blotting

RNA samples were denatured, resolved by formaldehyde agarose electrophoresis and blotted to Gene Screen (Dupont) by high salt capillary transfer according to standard procedures. Blots were probed with random hexamer labeled DNA probes using Prefect Hyb hybridization solution (Sigma) at 65°C according to the manufactures procedures. Probes used were the Probe B and Exon2 probe described above. Blots were washed with four times with 1× SSC for 10 minutes at 65°C. Phosphoimager was used to detect the hybridization signal.

### Western Blotting

Protein samples were diluted in SDS sample buffer and resolved by PAGE. 50 µg total protein was transferred to PVDF (Biorad) using 10 mM CAPS, pH 10.5, 15% methanol, 3.5 mM DTT transfer solution for 17 hours at 20 mA and 20 V limits. Membranes were blotted with affinity purified anti-Bptf at 1∶10000 dilution [14], M2 anti Flag monoclonal antibody at 1∶5000 (Sigma), anti Smad2 and Smad2-phos (Cell Signaling Technologies) 1∶1000, anti SNF2H/L (Abcam) 1∶5000, anti-CBP and p300 1∶100 (Abcam) overnight at 4°C then anti rabbit or mouse HRP (Amersham) at 1∶20000 dilution for 2 hrs at RT in PBS with 5% NFDM and 0.1% tween 20 through out.

Histone Western blotting was performed essentially as above. Protein samples were extracted from ES cells grown on gelatinized plates in ES cell growth media containing 100 U/ml LIF using TriReagent according to manufacturer's procedures (Sigma). 50 µg of total protein was resolved by 15% SDS PAGE and transferred to PVDF (Biorad) using 12.5 mM Tris, 96 mM glycine pH 8.3, 20% methanol transfer solution for 1 hour at 200 mA and 25 V limits. Blots were probed with rabbit polyclonal antibodies to H4 ac-K5, H4 ac-K8, H4 ac-K12 (Serotech), H4 3me-K20, H4 acK16, H3 3me-K9, H3 3me-K27 (Upstate), g-H2AX, H3 3me-K4, H3 ac-K14, H3 ac-K18 (abcam) at 1∶2000 dilutions overnight at 4°C then anti rabbit HRP (Amersham) at 1∶4000 dilution for 1 hrs at RT in PBS with 5% NFDM and 0.1% tween 20 through out. Detection was preformed using Pico signal ECL according to manufacturer's procedures (Pierce).

### Tissue Culture Procedures and Luciferase Assays

ES cells were maintained on mitomycin C treated MEFs in ESC Growth Media (15% FCS (ES Cell grade, Invitrogen), DMEM, essential amino acids, 10 µM mercapto-ethanol, 2 mM glutamine, 100 U/ml LIF (Chemicon International), penicillin and streptomycin). Culture techniques used were described previously [66]. Lines were passaged 3 times onto gelatinized plates to remove MEFs prior to experiments.

Growth rates of ES cell lines were obtained on gelatinized plates in the absence of feeder layers. Cells were plated @ 1.0×10^5^ in 6 well plates. Cell number was determined every day for 4 consecutive days with changes in ESC Growth media occurring every day.

Activin-A stimulation of ES cells was performed as follows. ES cell lines were started on gelatinized plates in ESC Growth Media. Cultures were then grown for 2 days in ESC Growth Media without LIF. On the third day, cells were cultured in low serum ESC Growth Media without LIF (same as ESC Growth Media except 0.1% FCS is used instead of 15% FCS) in the presence or absence of 30 ng/ml activin-A (R & D Systems) overnight. Cells were processed using TriReagent according to manufactures standard protocol.

p300, CBP siRNA knockdowns were preformed as follows. ES cells were grown in ES Cell Growth Medium with 100 U/ml LIF on gelatinized plates. Cells were collected using trypsin and transfected using an Amaxa Nucleofector device using solution ES Cell and program A-30 according to manufacturer's procedures. 2.0×10^6^ ES cells were transfected with 400 nmoles siRNA duplex (Darmacon) or a GFP-Max nucleofection control. Post nucleofection 1.0×10^6^ cells were plated to each well of a 6 well gelatinized tissue culture plate. Cells were stimulated with or without Activin-A in low serum ES Cell growth media as described above. Cells at specified time points were lysed using TriReagent reagent (Sigma) and RNA and protein were purified according to manufacturer's procedures.

MCF10CA1h cells were grown in a 1∶1 mixture of DMEM and Ham's F12 medium (Gibco) supplemented with 5% horse serum. Cells were collected using trypsin and transfected using an Amaxa Nucleofector device using solution V and program T-27 according to manufacturer's procedures. 2.0×10^6^ cells were transfected with 400 nmoles siRNA duplex (Darmacon) or a GFP-Max nucleofection control. Post nucleofection 1.0×10^6^ cells were plated to each well of a 6 well tissue culture plate. Cells were grown for 2 days in growth media and then serum deprived in 1% FBS, DMEM, nonessential amino acids, 10 µM β-mercaptoethanol overnight. Following serum shock cells were incubated with or without 5 ng/ml TGF-β1 (R&D Systems) for 1 hour. Cells were lysed using TriReagent reagent (Sigma) and RNA and protein were purified according to manufacturer's procedures.

P19 cells were obtained from the ATCC (ATCC Number CRL-1825). Cells were grown in 10% FBS, DMEM, 2 mM glutamine, penicillin and streptomycin. Cells were transfected using an Amaxa Nucleofector device using solution V and program C-20 according to manufacturer's procedures. 2.0×10^6^ P19 cells were transfected with 400 nmoles siRNA duplex (Darmacon) or a GFP-Max nucleofection control. Post nucleofection 3.0×10^5^ cells were plated to 12 well plates. Cells were allowed to grow for two days then were serum starved for 18 hours in 0.1% FBS, DMEM, 2 mM glutamine, penicillin and streptomycin. Cells were induced with 5 ng/ml TGF-β1 (R&D Systems) in low serum growth medium overnight. Cells were lysed using TriReagent reagent (Sigma) and RNA and protein were purified according to manufacturer's procedures.

Luciferase assays were performed in 12 well format by transfecting 0.6 µg pGL3ti-(SBE)4 or pAR3-Luc, 0.012 µg pCH110 with or without 0.6 µg TβRI or 0.6 µg Smad 2-HA and 0.006 µg Smad4-HA, 40.0 pmoles siRNA duplex per well using Lipofectamine 2000 (Invitrogen). In experiments using TGF-β1 (R&D Systems) or Activin-A (R&D Systems) cells were allowed to grow for 48 hours in 10% FBS, DMEM, 2 mM glutamine prior to adding TGF-β1 at 5 ng/ml or Activin-A at 30 ng/ml. Cells were induced for 24 hours then lysed with 250 µl siGlow Lysis Buffer (Promega) according to manufacturer's procedures. Transfections utilizing the constitutively active receptors were incubated for 48 hours prior to lysis as above. 50 µl or 100 µl of lysate was assayed with 100 µl Bright-Glow Luciferase Assay Reagent (Promega). Transfection efficiency was normalized to β-galactosidase levels by assaying 10 µl lysate to 100 µl Beta-Glo Assay Reagent (Promega). Relative Luciferase units were obtained by dividing the luciferase activity levels by the β-galactosidase levels. Experiments were repeated at least twice and yielded essentially the same results.

We used P19 cells to test specificity of TGFβ induction of Bptf protein levels. P19 cells were grown in 10% FBS, DMEM, 2 mM glutamine, penicillin and streptomycin. Ligands were added at the following concentrations; TGFβ1 (R&D Systems) at 5 ng/ml, FGF4 (R&D Systems) and heparin at 25 ng/ml and 1 ug/ml respectively, BMP4 (R&D Systems) at 10 ng/ml and Wnt3a or L cell control conditioned medium at 1∶1 with 10% FBS, DMEM, 2 mM glutamine, penicillin and streptomycin. Cells were incubated overnight and harvested for protein with TriReagent reagent (Sigma) according to manufacturers procedures. Bptf Western blotting was performed as described above.

Embryoid bodies were cultured as follows. ES cells were treated with trypsin briefly to retain cells in medium sized clumps. Cells were then centrifuged at low speed and resuspended in ESC Growth Medium without LIF and cultured in bacteriological grade petri dishes. ESC Growth Medium without LIF was changed every day for 9 days with minimal disturbance to the embryoid bodies. Samples were taken at specified time points using TriReagent according to manufactures standard protocol or fixed in 10% NBF for histology.

Knockout of *Bptf* in MEFs was accomplished by infecting litter mate wild type and conditional *Bptf* (-/Floxed genotype) MEF lines with an adenovirus expressing CMV-Cre. Infection was performed in 6 well plates at sub-confluence (2×10^5^ cells) with 1 ml growth media containing 45 µl Adenovirus @ 1×10^10^ PFU/ml. Cells were grown for two days in the presence of virus. Following infection cells were divided into 5 wells of a 6 well plate. Cell numbers were recorded every 24 hours with changes in media occurring every 48 hours. A sample of cells was removed at day 4 for Western analysis of Bptf protein levels. Experiment was repeated with two wild type and conditional knockout MEF lines from littermate embryos.

### Chromatin Immuno Precipitation (ChIP)

ES cells were maintained on mitomycin C treated MEFs in ESC Growth Media (15% FCS (ES Cell grade, Invitrogen), DMEM, essential amino acids, 10 µM mercapto-ethanol, 2 mM glutamine, 100 U/ml LIF (Chemicon International), penicillin and streptomycin). Culture techniques used were described previously [66]. Lines were passaged 3 times onto gelatinized plates to remove MEFs prior to experiments.

Activin-A stimulation of ES cells was performed as follows. ES cell lines were started on gelatinized plates in ESC Growth Media. Cultures were then grown for 2 days in ESC Growth Media without LIF. On the third day, cells were cultured in low serum ESC Growth Media without LIF (same as ESC Growth Media except 0.1% FCS is used instead of 15% FCS) in the presence or absence of 30 ng/ml activin-A (R & D Systems) overnight. Following activin-A induction cells were washed with PBS and fixed in 2 mM EGS (Pierce) in 25% DMSO/75% PBS for 30 min followed by 1% PFA in PBS for 30 min. For histone ChIP, cells were fixed in 1% PFA in PBS for 15 min. Following fixation cells were washed 3X in PBS and removed from the tissue culture dish with a cell scraper. Cell pellets were then frozen at −80°C. The following day the pellets were thawed and processed using the ChIP procedure published by Upstate biologicals. Antibodies used for pulldown were ChIP grade SNF2H/L (Abcam), H3 3me-K4, H3 3me-K27 (Upstate) and pan H3 (Abcam).

Quantitation of pulldown was performed by real time PCR using 2× DyNAmo syprogreen qPCR kit (New England Biolabs) according to manufacturers procedures. Briefly, reactions were composed of 5 µl 1.2 µM forward and reverse primers, 5 µl diluted cDNA template and 10 µl 2× qPCR mix (see Table S3 for primer sequences). Reactions were heated for 10 min at 94°C, then cycled for 40 cycles at 20 sec at 94°C, 20 sec at 60°C, 30 sec at 72°C. After each cycle the sample was heated to 78°C for 10 sec prior to reading sample fluorescence. Pulldowns were quantified as a percentage of input using a dilution series as a standard curve. Histone modification pulldowns are expressed as enrichment relative to histone H3 occupancy under +activin-A conditions. SNF2H/L pulldowns were first normalized to signal at *Gapdh* and are expressed as the enrichment during +activin-A relative to −activin-A conditions.

### QPCR

RT reactions were performed on RNA extracted from ES cells, embryoid bodies or P19 cells using TriReagent according to manufacturer's procedures. 5 µg of total RNA was reverse transcribed with Superscript II using oligo dT priming according to manufactures procedures (Invitrogen). cDNA reactions were diluted 5–10 fold and used in a template for PCR.

Real time PCR was performed using 2× DyNAmo syprogreen qPCR kit (New England Biolabs) according to manufacturers procedures. Briefly, reactions were composed of 5 µl 1.2 µM forward and reverse primers, 5 µl diluted cDNA template and 10 µl 2× qPCR mix (see Table S3 for primer sequences). Reactions were heated for 10 min at 94°C, then cycled for 35 cycles at 20 sec at 94°C, 20 sec at 60°C or 52°C, 30 sec at 72°C. After each cycle the sample was heated to 78°C for 10 sec prior to reading sample fluorescence. Reactions were done in triplicate. ΔΔCt method was used to quantify the relative levels of expression to the *Gapdh* or *β-actin* house keeping genes. Expression levels were then normalized to un-induced cells control cells.

QPCR was performed on E7.5 embryo cDNA as follows. Briefly, reactions were composed of 5 µl 1.2 µM forward and reverse primer pair, 5 µl diluted cDNA template and 10 µl 2× qPCR mix (see Table S3 for primer sequences). Reactions were heated for 10 min at 94°C, then cycled at 20 sec at 94°C, 20 sec at 60°C, and 30 sec at 72°C. The number of cycles to maintain linearity was determined using the real time analysis software. PCR reactions within the linear range were resolved using native PAGE.

### Microarray

For microarray experiments we used one wildtype and two independent Bptf mutant cell lines in 3 culture conditions (LIF+, LIF−, and RA+). Experiments were carried out with 3 biological replications. At the day 3, Triazole (1 ml/well; Invitrogen, USA) was added to the well and total RNAs were extracted using Phase lock gel columns (Eppendorf/Brinkman) according to the manufacturer's protocol. Total RNAs were precipitated with isopropanol, washed with 70% ethanol, and dissolved in DEPC-treated H_2_O. 2.5 g of total RNA samples were labeled with Cy3-CTP using a Low RNA Input Fluorescent Linear Amplification Kit (Agilent, USA). A reference target (Cy5-CTP-labeled) was prepared from the Universal Mouse Reference RNA (Stratagene, USA). Labeled targets were purified using an RNeasy Mini Kit (Qiagen, USA) according to the Agilent's protocol, quantified by a NanoDrop scanning spectrophotometer (NanoDrop Technologies, USA), and hybridized to the NIA Mouse 44K Microarray v2.2 (whole genome 60-mer oligo; manufactured by Agilent Technologies, #014117) [68]. Transcript copy number estimation using a mouse whole-genome oligonucleotide microarray according to the Agilent protocol (G4140-90030; Agilent 60-mer oligo microarray processing protocol - SSC Wash, v1.0). All hybridizations were carried out in the two color protocol by combining one Cy3-CTP-labeled experimental target and Cy5-CTP-labeled reference target. Microarrays were scanned on an Agilent DNA Microarray Scanner, using standard settings, including automatic PMT adjustment.

Differential gene expressions in various cell lines in the standard culture condition were analyzed using the NIA Array Analysis software (http://lgsun.grc.nia.nih.gov/ANOVA/) which implements ANOVA statistics with two additional methods to reduce the number of false positives: (1) small error variances were replaced with the average error variance estimated from 500 genes with similar signal intensity, and (2) false discovery rates (FDR<0.05) were used to select genes with differential expression, instead of p-values [69]. The FDR method accounts for the effect of multiple hypotheses testing.

Gene Ontology analysis and clustering was accomplished using DAVID (http://david.abcc.ncifcrf.gov/) and the NIA Mouse Gene Index (http://lgsun.grc.nia.nih.gov/geneindex/mm8) using default settings [70].

### GST Pulldowns

*In vitro* GST pulldown assays were performed in 50 µl volumes in binding buffer (25 mM Hepes pH 7.4, 100 mM NaCl, 0.5 mM MgCl_2_, 0.01% NP40), ∼10 µg GST protein fusion bound to a glutathione Sepharose support, 100 ng human NURF complex. Proteins were allowed to bind for 1 hour at 4°C with occasional mixing. The beads were washed with 500 µl binding buffer three times at 4°C and re-suspended in a final volume of 30 µl.

NURF and CBP pulldowns from ES Cell extracts were preformed essentially as described above from 500 mM KCl nuclei extractions made as previously described [71]. The extract was diluted 1∶3 with 25 mM Hepes pH 7.4, 0.5 mM MgCl_2_, 0.01% NP40 with protease inhibitors (Roche) prior to binding to resin bound GST-Smads. The beads were washed with 500 µl binding buffer three times at 4°C and re-suspended in a final volume of 30 µl. Proteins were eluted from the beads by adding 5 µl 6× SDS sample buffer and incubating at 37°C for 30 min. Proteins were resolved on 4% or 8% polyacrylamide gels and transferred to PVDF (Biorad) and prepared for Western blotting as described above.

## Supporting Information

Figure S1 Bptf is the homolog of NURF 301.
(A) Mouse Bptf is highly homologous to Drosophila NURF301. Major functional domains are conserved between mammals and flies, including nucleosome and transcription factor binding sites, strongly suggesting that Bptf is the homolog of the largest subunit of the Drosophila NURF complex, NURF301. (B) Bptf is predominantly nuclear in cultured P19 cells. siRNA targeting of *Bptf* results in decreased Bptf signal by Western analysis and a reduction of nuclear fluorescence by anti-Bptf immuno fluorescence in P19 cells. (C) Western analysis of adult brain, testis, spleen, and embryonic stem cells reveals two migrating species, Bptf-H and Bptf-L. (D) Western analysis of total protein extracts from adult tissues using the affinity purified anti-Bptf antisera identifies a single band of high molecular weight predominately found in testis, spleen, and brain. In addition to full length Bptf, we identify a single ∼100 kDa protein which reacts with the antibody exclusively in the adult brain. This protein is identical in size to the previously reported *Bptf* splice variant FAC1 (Bowser R, Giambrone A, Daves P (1995) Dev Neuroscience 17: 20–37.). (E) A similar western analysis of protein extracts from total embryo proteins identifies high Bptf expression throughout development. The ∼100 kDa cross-reacting band was only found in the adult brain loading standard and not during development.
(1.28 MB TIF)

Figure S2 Generation of conditional and gene trap alleles of *Bptf*.
(A) Intron-exon map of mouse Bptf showing alternatively spliced exons and the position of the *Bptf^XG023^* trapping vector and *Bptf^ΔExon2^* floxed exon. (B) Diagram of *Bptf* and *Bptf^XG023^* alleles showing tandem trapping vector insertion site between exons 15 and 16. Restriction enzyme sites and positions of Southern blotting probes used for the characterization of *Bptf^XG023^* are shown as external probe A and internal probe B. Positions of primers used in multiplex PCR genotyping are designated A, B, and C with A and B complementing genomic sequence spanning the trapping vector insertion site and C the trapping vector. (C) A Southern blot using probe A showing diagnostic restriction enzyme sites confirming the insertion of the trapping vector into *Bptf*. (D) A Southern blot using Bgl II and probe B showing two bands characteristic of multiple integration events by the trapping vector. Upper band contains sequences external and lower band internal to the trapping vector. (E) A Southern blot of genomic DNA digested with Nsi I, resolved by FIGE and blotted with probe B. A single band was identified in heterozygous mice with an approximate size of 30 Kb, suggesting 3 to 4 trapping vector integration events. (F) Results from multiplex PCR analysis of wild type (+/+), heterozygous (+/-), and homozygous (−/−) *Bptf^XG023^* E7.5 embryos using primers A, B, and C. The wild type and mutant alleles are identified by a 325 bp and 500 bp product, respectively. (G) Diagram of *Bptf*, *Bptf^Floxed-Neo^*, *Bptf^Floxed^*, and *Bptf^ΔExon2^* alleles showing the configuration of the integrated targeting vector and its Cre and Flp reaction products. Restriction enzyme sites and positions of Southern blotting probes used for the characterization of *Bptf^ΔExon2^* are shown as external probe 29–31 and internal probes 20.2–20.7, exon2 and 25–26. Positions of primers used in multiplex PCR genotyping are designated A, B, C, E, and F. (H) A Southern blot of heterozygous *Bptf^Floxed-Neo^*, *Bptf^Floxed^*, and *Bptf^ΔExon2^* alleles using Sca I and probe 25–26 showing diagnostic restriction enzyme sites confirming the insertion of the targeting vector into *Bptf* and the products of Cre and Flp recombination. (I) A Southern blot of the same mice as in (H) using Eco RI and probe 25–26. (J) A Southern blot of the same mice as in (H) using EcoRI and probe 29–31. (K) A Southern blot of same mice as in (H) using Eco RI and probe 20.2–20.7. (L) A Southern blot of same mice as in (H) using Kpn I and probe 20.2–20.7. (M) Results from multiplex PCR analysis of wild type *Bptf* and heterozygous *Bptf^Floxed-Neo^*, *Bptf^Floxed^*, and *Bptf^ΔExon2^* alleles using primers E, F, and C. The multiplex PCR strategy yields band sizes of *Bptf* = 375 bp, *Bptf^Floxed^*  =  475 bp, and *Bptf^ΔExon2^* = 650 bp. (N) Results from multiplex PCR analysis of wild type *Bptf* and heterozygous *Bptf^Floxed-Neo^*, *Bptf^Floxed^*, and *Bptf^ΔExon2^* alleles using primers A, B, and C. The multiplex PCR strategy yields band sizes of *Bptf* = 250 bp, *Bptf^Floxed-Neo^*, *Bptf^Floxed^*  =  325 bp, and *Bptf^ΔExon2^* = 500 bp.
(1.89 MB TIF)

Figure S3 Molecular characterization of *Bptf^XG023^* allele.
(A) PCR amplification and sequencing of the upstream junction between the trapping vector and the mouse genome confirm the exact position of the trapping vector to be between exon 15 and 16 of *Bptf*. (B) RT-PCR results from E7.5 embryos show reduced expression of 3′-RNA sequences and severely reduced exon 15–16 splicing, confirming efficient trapping of *Bptf* by the trapping vector insertion. (C) DNA sequence of a 5′-RACE amplicon of the *Bptf^XG023^* allele from testis shows an in-frame fusion between exon 15 of *Bptf* and *β-Geo* from the trapping vector. (D) Northern analysis of RNA from wild type and heterozygous *Bptf^XG023^* disruption mice show predominant transcripts in testis, lungs, spleen, and brain using a probe specific for *Bptf* mRNA. The *Bptf-β-Geo* fusion RNA has the same expression patterns as the wild type allele, suggesting that the trapping vector does not interfere with *Bptf* regulation at the level of transcription.
(1.34 MB TIF)

Figure S4 Molecular characterization of *Bptf^ΔExon2^* allele.
(A) RT-PCR results from E7.5 embryos show slightly reduced expression of 3′-RNA sequences of *Bptf^ΔExon2^* at the RNA level. (B) RT-PCR results from E7.5 embryos show deletion of exon 2 RNA sequences from *Bptf^ΔExon2^* transcripts. Primers used were complementary to exons 1 and 8. (C) RT-PCR products from *Bptf^ΔExon2^* transcripts in (B) were sequenced to identify out of frame splicing events, strongly suggesting that the exon 2 deletion results in efficient knockout of the Bptf protein.
(0.64 MB TIF)

Figure S5 *Bptf^ΔExon2^* and *Bptf^XG023^* alleles are genetically equivalent.
(A) Wild type (+/+), heterozygous (+/−), and homozygous (−/−) *Bptf^ΔExon2^* embryos at E5.5, E6.5, and E7.5 were removed from their decedua and genotyped using a PCR-based method. Identical to the *Bptf^XG023^* allele, reduced growth rates are evident in the homozygous *Bptf^ΔExon2^* embryos as early as E5.5. Mutant embryos from both lines maintained the gross morphology of E5.5 embryos at E6.5 and E7.5 with an overall increase in size. (B) Heterozygous *Bptf^XG023^* and *Bptf^ΔExon2^* mice were intercrossed, and wild type, *Bptf^ΔExon2^* heterozygous, *Bptf^XG023^* heterozygous, and *Bptf^XG023^* / *Bptf^ΔExon2^* trans-heterozygous embryos were dissected from their decidua at E6.5, and genotypes were confirmed by PCR. Trans-heterozygous embryos phenocopy homozygous *Bptf^XG023^* or *Bptf^ΔExon2^* embryos, suggesting that the two alleles have an equivalent phenotype.
(3.04 MB TIF)

Figure S6 *Bptf* mutants do not have significant growth defects prior to implantation.
Wild type (+/+), heterozygous (+/−), and homozygous (−/−) *Bptf^XG023^* or *Bptf^ΔExon2^* E3.5 blastocysts and 5-day blastocyst outgrowths did not reveal any significant morphological or growth differences in trophoblast or the inner cell mass.
(1.36 MB TIF)

Figure S7 *Bptf* mutants have reduced cellular proliferation.
Percent phosphorylated histone H3 positive cells from total cells of the control and mutant embryos were quantified to show reduced cellular proliferation of *Bptf* mutant embryos at E5.5, E6.5, and E7.5.
(0.15 MB TIF)

Figure S8 *Bptf^XG023^* is widely expressed in the embryo proper.
(A–D) Wild type and heterozygous embryos were stained in whole mount for β-galactosidase activity at E5.5 and E6.5. *Bptf^XG023^* expression is present in E5.5 and E6.5 embryos in embryonic but not extra-embryonic tissues. (E–G) Wild type and heterozygous E7.5 embryos were stained in whole mount for β-galactosidase activity. *Bptf^XG023^* expression is present at E7.5 embryos in embryonic but not extra-embryonic tissues. (a′) Cross section of the E7.5 heterozygous embryo shows expression exclusive to the embryonic ectoderm and little to no expression in the visceral endoderm. (b′) Cross-section of the E7.5 heterozygous embryo shows expression primarily in the embryonic ectoderm, reduced expression in the mesoderm, with little to no expression in the visceral endoderm. Abbreviations: ve, visceral endoderm; ee, embryonic ectoderm; m, mesoderm.
(0.34 MB TIF)

Figure S9 *Bptf^XG023^* expression during embryonic development.
(A–E) Wild type (+/+) and heterozygous (+/−) *Bptf^XG023^* embryos were dissected from their decidua and stained in whole mount for β-galactosidase activity. Staining is seen predominantly in the embryonic tissues at E7.5 (A) and E8.5 (B). At later stages of development, expression is ubiquitous in the embryo (C–E). (F) Sagittal section of E10.5 embryo stained for β-galactosidase activity reveals the extent of *Bptf^XG023^* expression. Expression was observed in many tissues originating from mesoderm (notochord, myocoele, somites), endoderm (lung bud), and ectoderm (forebrain, midbrain, hindbrain, and neural tube). Scale Bar = 400 µm.
(4.01 MB TIF)

Figure S10 An analysis of *Nodal* expression at E5.5 shows *Bptf* mutants express *Nodal* at levels comparable to wild type.
(A) *Bptf* mutants express *Nodal* in the visceral endoderm at E5.5 at comparable levels to the wild type control. (B) *Bptf* mutants express delocalized *Nodal* at E6.5.
(1.94 MB TIF)

Figure S11 *Bptf* mutants cannot establish an anterior–posterior axis.
(A,B) Whole mount in situ RNA hybridization analysis of wild type and mutant E6.5 and E7.5 embryos for *Cer1*, *Nodal*, *Lefty1*, *Hex1*, *Otx2*, *Hesx1*, *Lhx1*, *T*, *Gsc*, *Fgf8*, *Foxa2*, *Cripto*, *Junb*, *Mash2*, *Bmp4*, *Erbb2,* and *Fgfr2* expression. An analysis on wild type and *Bptf* mutant E6.5 embryos for anterior visceral endoderm (AVE) markers *Cer1*, *Lefty1*, and *Hex1* show that mutants do not develop an AVE. *Bptf* mutants show comparable but delocalized *Nodal* and *Cripto* expression at E6.5, further suggesting a defect in the development of the AVE. Embryos showed reduced expression of the embryonic ectoderm marker *Otx2* and *Hesx1* in mutants compared to wild type controls, suggesting defects in the embryonic ectoderm. Primitive streak markers *Lhx1*, *T, Gsc, Fgf8,* and *Foxa2* also show a significant reduction in expression in mutant at E6.5 compared to wild type. The extra-embryonic markers *Bmp4*, *Erbb2*, *Fgfr2,* and *Mash2* are not dramatically affected in mutant when compared to wild type embryos at E6.5. The cell cycle regulatory gene *Junb* was found to be over expressed in mutant relative to wild type controls at E6.5. Analysis of E7.5 embryos confirms that *Bptf* mutants do not establish an anterior–posterior axis. *Lhx1* is not significantly expressed at E7.5 in mutant compared to wild type, suggesting that primitive streak does not form. Expression of mesoderm markers *T* and *Fgf8* is diffuse in mutant embryos compared to wild type at E7.5, suggesting that the embryo proper becomes unorganized mesoderm-like tissue. The extra-embryonic markers *Bmp4* and *Mash2* maintain extra-embryonic expression in E7.5 mutant embryos. Angiogenesis markers *Vegf* and *Flk1* show little expression in the extra embryonic portion of the embryo as compared to wild type controls. However, expression is not associated with an allantois, which is severely underdeveloped in *Bptf* mutant embryos. *Bptf* expression is largely confined to the embryonic tissues at E7.5.
(6.17 MB TIF)

Figure S12 Construction of *Bptf* mutant embryonic stem (ES) cell lines.
(A) Retargeting and subsequent transient Cre expression generated conditional heterozygous (not shown), heterozygous (+/−), conditional homozygous (Floxed-Neo/-), and homozygous (−/−) mutant *Bptf* mouse ES cell clones. Genotypes were confirmed by Southern analysis using an Eco R1 digest and hybridization with Probe 20.2–20.7. (B) Northern analysis of *Bptf*-mutant ES cell clones using a probe to the 3′-end of the gene shows reduced *Bptf* transcript levels. (C) RT-PCR analysis of wild type (+/+) and homozygous mutant (−/−) RNA confirms the complete deletion of exon 2 from *Bptf* transcripts in homozygous mutant clones. Sequence from *Bptf* mutant PCR products shows the *Bptf* transcript contains exon 1–3 and 1–4 out of frame splice events as seen in Figure S4C (data not shown). (D) Western analysis of Bptf wild type, heterozygous, conditional heterozygous, and mutant ES cells. Heterozygosity at *Bptf* results in reduced Bptf protein levels, raising the possibility of haploinsufficiency phenotypes. Homozygous *Bptf* ES cells mutants do not show Bptf protein, demonstrating that *Bptf^ΔExon2^* is likely to be a null allele. FAC1 expression was not found in ES cells.
(0.71 MB TIF)

Figure S13 Conditional deletion of *Bptf* in mouse embryonic fibroblasts (MEF) with adenovirus expressing Cre recombinase.
Conditional Bptf mutant (−/Floxed) and control (+/+) MEF lines were infected with an adenovirus expressing the Cre recombinase. Infected (+Cre) and mock infected (−Cre) cells were passaged once to deplete the cellular stores of the Bptf protein. Following growth for 4 days, cells were harvested for total protein. Bptf protein was detected by Western using ponceau S staining as a loading control.
(0.57 MB TIF)

Figure S14 *Bptf* mutant embryonic stem (ES) and mouse embryonic fibroblast (MEF) cell lines show slight growth defects.
(A) The doubling time of wild type and mutant ES cells was investigated. We observed that *Bptf* mutants but not conditional heterozygous or heterozygous ES cells showed reduced growth rates relative to wild type (+/+) cell lines. A cell cycle analysis of slower growing *Bptf* knockout ES cell lines shows that they have a slight G1 to S phase delay. (B) The doubling time of wild type and mutant MEF lines was investigated. We observed that *Bptf*-depleted MEF lines showed slightly reduced growth rates relative to control lines.
(0.24 MB TIF)

Figure S15 *Bptf* mutant mouse embryonic stem (ES) cells do not differentiate as embryoid bodies, are not apoptotic, and proliferate normally.
(A) Day 9 embryoid bodies were sectioned and stained for gross analysis with hematoxylin and eosin (H&E), the proliferation markers PCNA and phosphorylated histone H3, and apoptosis by TUNEL. *Bptf*-mutant (−/−) embryoid bodies contained few differentiated and TUNEL positive apoptotic cells compared to wild type (+/+) controls. TUNEL negative *Bptf* mutant (−/−) and wild type (+/+) cells have similar staining for proliferation markers PCNA and phosphorylated histone H3. (B) Microscopy of wild type and *Bptf* mutant ES cells during a 9-day embryoid body differentiation time course. *Bptf* mutant ES cells did not develop the size and cystic structures of the wild type controls. (C) Wild type but not mutant day 9 embryoid bodies develop a prominent layer of endoderm on the exterior surface. In place of endoderm *Bptf* knockout, embryoid bodies develop a thin epithelial cell layer.
(3.46 MB TIF)

Figure S16 *Bptf*-mutant embryoid bodies show little to modest deregulation of cell cycle and pluripotency markers.
Gene expression was monitored from total RNA during a 9-day embryoid body differentiation time course. RT-PCR analysis for cell cycle markers (A), pluripotency markers (B).
(0.93 MB TIF)

Figure S17 Transcription and differentiation defects in *Bptf^ΔExon2^* knockout embryonic stem (ES) cells are rescued by reintroduction of *Bptf^Floxed-Neo^*.
(A) *Bptf^ΔExon2^*/*Bptf^ΔExon2^* ES cell lines were retargeted with the *Bptf^Floxed-Neo^* targeting vector to generate *Bptf^ΔExon2^*/*Bptf^Floxed-Neo^* rescue lines. Recombination of the targeting vector at *Bptf^ΔExon2^* was confirmed by Southern blotting Sca I digested DNA and probing with Probe 25–26. (B) A Western analysis of total protein from parental *Bptf^ΔExon2^*/*Bptf^Floxed-Neo^*, *Bptf^ΔExon2^*/*Bptf^ΔExon2^*, and *Bptf^ΔExon2^*/*Bptf^Floxed-Neo^* rescue lines show reintroduction of the Bptf protein. (C) Expression analysis of mesoderm markers *T, Gsc,* and endoderm markers *Sox17, Cer* in a *Bptf^ΔExon2^*/*Bptf^Floxed-Neo^* rescue ES cell line during a 9-day embryoid body differentiation time course. Reintroduction of *Bptf^Floxed-Neo^* rescued the induction of mesoderm and endoderm markers during embryoid differentiation.
(0.90 MB TIF)

Figure S18 Gene ontology clustering analysis of *Bptf*-dependent genes by condition.
The entire array dataset from each condition was analyzed for statistically significant ontology clusters using DAVID analysis software. Genes that were repressed in expression cluster to highly similar ontology groups and include those involved in “development”, “transcription factor activity”, and “morphogenesis”. Activated gene clusters are highly dependent on condition and include “antigen processing”, “peptidase inhibitors”, and “cytoskeletal components”.
(0.88 MB TIF)

Figure S19 *Bptf* negatively regulates many homeobox transcription factors.
(A) Genes containing a homeobox but not the helix loop helix domains are significantly enriched in all categories of up-regulated gene sets. (B) Expression levels of homeobox transcription factor gene clusters displayed as a heat map. Almost the entire a, b, and c clusters are deregulated, suggesting that *Bptf* plays an important function in regulating clusters of homeobox domain–containing transcription factors.
(0.35 MB TIF)

Figure S20 *Bptf* preferentially potentates dynamically regulated genes with bivalent promoters.
(A) Genes whose transcription activates, represses, or is unchanged upon LIF- conditions or the addition of retinoic acid (RA) were analyzed for *Bptf* dependence. Knockout of *Bptf* results in predominantly reduced activation (repression of activated genes with *Bptf* knockout) or reduced repression (activation of repressed genes with *Bptf* knockout) of dynamically regulated genes. (B) *Bptf* preferentially regulates promoters with bivalent histone modifications. The methylation state expressed as a percentage of all promoters from undifferentiated mouse ES cells. Data was retrieved from Bernstein et. al. (2006) Cell 125:315–326. *Bptf*-dependent genes from the microarray dataset were grouped based on the promoter's methylation state. From this analysis, *Bptf*-dependent genes are predominantly enriched for bivalent histone H3 3me-K27, histone H3 3me-K4 over single modified or unmodified promoters. (C) The magnitude of deregulation was compared between activated and repressed genes for each condition of *Bptf* dependence. A breakdown of gene classes by condition shows that the transcriptome is repressed under LIF+ and RA growth conditions and activated under LIF-. (D) The modifications on bulk histones in *Bptf* mutant embryonic stem (ES) cells are consistent with a transcriptionally repressed chromatin state. Bulk histones were prepared from wild type (+/+) and two *Bptf* mutant (−/−) ES cell lines grown in LIF+ growth media. Total protein extracts were then probed for histone modifications by Western blotting. *Bptf* knockout ES cells have an increase of the repressive H3 3me-K9 and H3 3me-K27 and a decrease of the activating marks H3 ac-K18 and H4 ac-K5. Interestingly, knockout ES cells have an increase in levels of γ-H2AX, a mark correlated with DNA damage, and H3 3me-K36, a mark of transcription elongation.
(0.97 MB TIF)

Figure S21 *Bptf*-dependent genes are frequently located in clusters.
The chromosome position for each *Bptf*-dependent gene was identified, and statistically significant gene clusters are shown with a letter designation. Clusters include genes with similar regulation and imprinted clusters.
(1.80 MB TIF)

Figure S22 Western analysis of Bptf, CBP/p300 knockdown and Smad2 activation during TGF-β1 and activin-A stimulation.
(A) Wild type and *Bptf* mutant embryonic stem (ES) cells were induced with activin-A for 24 hours. Western analysis shows up-regulation of Bptf protein levels and the phosphorylation of Smad2 in wild type and *Bptf* mutant ES cells with the addition of activin-A. (B) TGF-β1 selectively increases protein levels of Bptf in P19 cells. P19 cells were treated with TGF-β1, BMP4, Wnt3a-conditioned media, and FGF4+heparin for 24 hours. Following induction, cells were harvested and analyzed for Bptf levels by Western blotting. TGF-β1 increased and Wnt3a decreased Bptf protein levels. (C) Wild type ES cells were subjected to siRNA mediated knockdown of Bptf and CBP/p300, followed by induction with activin-A. Western analysis shows siRNA mediated knockdown of Bptf and CBP/p300 and phosphorylation of Smad2 with the addition of activin-A.
(1.28 MB TIF)

Figure S23 *Bptf* is necessary for Smad mediated gene transcription of cell cycle regulators.
RT-PCR analysis of Smad target genes from activin-A–induced embryonic stem cells identifies *Bptf*-dependent and -independent gene targets. The repression of p21 during stimulation with activin-A is dependent on *Bptf*.
(0.21 MB TIF)

Figure S24 Bptf is necessary for activation of Smad-responsive promoter elements.
(A) Knockdown of Bptf in P19 cells renders Smad responsive Smad binding elements (SBE) unresponsive to Smad activation via TGF-β. Luciferase activity was measured from stimulated or unstimulated P19 cells transfected with *Bptf* or mock siRNAs in addition to pGL3ti-(SBE)4 and LacZ transfection control plasmid. (B) Knockdown of Bptf in P19 cells renders Smad responsive ARE elements unresponsive to Smad activation via activin-A. Luciferase activity was measured from stimulated or unstimulated P19 cells transfected with *Bptf* or mock siRNAs in addition to pA3-Luc, CMV-Fast2-myc, and LacZ transfection control plasmid. (C) Co-transfection of HA-Smads 2,4 and constitutively active TβRI results in increased activation of luciferase reporter regulated by the SBE. (D) Knockdown of Bptf in P19 cells renders SBE Smad responsive elements unresponsive to constitutively activated Smad transcription factors. Luciferase activity was measured from P19 cells transfected with *Bptf* or mock siRNAs and combinations of Smad transcription factors, constitutively active type I receptor TβRI*, pGL3ti-(SBE)4, and a LacZ transfection control plasmid. (E) Western analysis showing siRNA mediated knockdown of Bptf in P19 cells during TGF-β1 induction. (F) Some TGF-β1-responsive gene targets in P19 cells are Bptf-dependent. Bptf knockdown during TGF-β1 activation significantly reduces the Smad mediated gene activation of *Cer* and *T*. (G) Western analysis showing siRNA mediated knockdown of BPTF in MCF10CA1 cells during TGF-β1 induction. (H) BPTF knockdown during TGF-β1 activation significantly reduces the Smad mediated gene activation of PAI-1 but not SMAD7.
(1.16 MB TIF)

Figure S25 *Bptf*-mutant embryos likely do not down regulate the Smad signaling pathway indirectly.
(A) RT-PCR analysis of E7.5 embryos shows normal expression of the BMP4 ligand, Smad 1,2 and 4 transcription factors, and receptors ALK3, Alk-IIB. Expression of the TGFβ-responsive gene *JunB* is significantly increased in mutant embryos. (B) Results from quantitative RT-PCR measurements of additional markers of early embryonic development in E7.5 embryos. Expression has been normalized to *GAPDH* levels and is expressed as the ratio mutant/WT. (C) An RT-PCR analysis of wild type and *Bptf*-mutant embryoid body differentiation time course for *Smad* transcription factors, *Bptf,* and the Smad responsive gene *Serpine1*.
(0.64 MB TIF)

Table S1 Disruption of *Bptf* results in early embryonic lethality.
(A) Wild type (+/+) and heterozygous (+/−) *Bptf^XG023^* males and females were mated in reciprocal crosses and all progeny were genotyped by Southern blotting or PCR. Homozygous (−/−) *Bptf^XG023^* embryos were not viable after E7.5 to E8.5, showing that mutations in *Bptf* are early embryonic lethal. Curiously, more reabsorptions were observed during the dissections than could be accounted for from the death of the homozygous *Bptf^XG023^* embryos. To investigate if the origin of the increased lethality was caused by a parental *Bptf^XG023^* heterozygous contribution, we crossed heterozygous to wild type males and females in combinations and examined all embryos from E10.5 to E18.5. From these experiments, we observed an increased number of reabsorptions when the mother was heterozygous compared to the wild type controls (Table S1A, b–d). The embryonic lethality was not biased toward heterozygous embryos, as they were found in the expected 1:1 ratio to wild type. These data suggested that *Bptf^XG023^* conferred a maternal effect on embryo survival. (B) As in (A), heterozygous (+/−) *Bptf^ΔExon2^* males and females were intercrossed and all progeny were genotyped by Southern blotting or PCR. As with *Bptf^XG023^*, homozygous (−/−) *Bptf^ΔExon2^* embryos were not viable after E7.5 to E8.5.
(0.33 MB TIF)

Table S2 *Bptf* mutants show highly similar phenotypes to Smad and Nodal mutants.
Phenotypes of *Bptf* mutants during blastocyst outgrowth and the establishment of the DVE, AVE, and primitive streak were compared to phenotypes from mutations of Smad2/4, Nodal, Wnt3/β-catenin, remodelers BRG1/SNF2H, and essential genes. *Bptf* mutations closely resemble mutations in Smad and Nodal pathways.
(0.30 MB TIF)

Table S3 Primer and siRNA sequences used in study.
Sequences of all siRNA duplexes and primers used in this study are shown in table format.
(1.47 MB TIF)

Dataset S1 Microarray and analysis datasets.
(21.56 MB XLS)

## References

[1] Jin J, Cai Y, Li B, Conaway RC, Workman JL (2005). In and out: histone variant exchange in chromatin.. Trends Biochem Sci.

[2] Lusser A, Kadonaga JT (2003). Chromatin remodeling by ATP-dependent molecular machines.. Bioessays.

[3] Margueron R, Trojer P, Reinberg D (2005). The key to development: interpreting the histone code?. Curr Opin Genet Dev.

[4] Eberharter A, Becker PB (2004). ATP-dependent nucleosome remodelling: factors and functions.. J Cell Sci.

[5] Bao Y, Shen X (2007). SnapShot: Chromatin Remodeling Complexes.. Cell.

[6] Corona DF, Tamkun JW (2004). Multiple roles for ISWI in transcription, chromosome organization and DNA replication.. Biochim Biophys Acta.

[7] Dirscherl SS, Krebs JE (2004). Functional diversity of ISWI complexes.. Biochem Cell Biol.

[8] Banting GS, Barak O, Ames TM, Burnham AC, Kardel MD (2005). CECR2, a protein involved in neurulation, forms a novel chromatin remodeling complex with SNF2L.. Hum Mol Genet.

[9] Mizuguchi G, Tsukiyama T, Wisniewski J, Wu C (1997). Role of nucleosome remodeling factor NURF in transcriptional activation of chromatin.. Mol Cell.

[10] Tsukiyama T, Wu C (1995). Purification and properties of an ATP-dependent nucleosome remodeling factor.. Cell.

[11] Badenhorst P, Xiao H, Cherbas L, Kwon SY, Voas M (2005). The Drosophila nucleosome remodeling factor NURF is required for Ecdysteroid signaling and metamorphosis.. Genes Dev.

[12] Xiao H, Sandaltzopoulos R, Wang HM, Hamiche A, Ranallo R (2001). Dual functions of largest NURF subunit NURF301 in nucleosome sliding and transcription factor interactions.. Mol Cell.

[13] Badenhorst P, Voas M, Rebay I, Wu C (2002). Biological functions of the ISWI chromatin remodeling complex NURF.. Genes Dev.

[14] Wysocka J, Swigut T, Xiao H, Milne TA, Kwon SY (2006). A PHD finger of NURF couples histone H3 lysine 4 trimethylation with chromatin remodelling.. Nature.

[15] Barak O, Lazzaro MA, Lane WS, Speicher DW, Picketts DJ (2003). Isolation of human NURF: a regulator of Engrailed gene expression.. Embo J.

[16] Williams CJ, Naito T, Arco PG, Seavitt JR, Cashman SM (2004). The chromatin remodeler Mi-2beta is required for CD4 expression and T cell development.. Immunity.

[17] Marfella CG, Ohkawa Y, Coles AH, Garlick DS, Jones SN (2006). Mutation of the SNF2 family member Chd2 affects mouse development and survival.. J Cell Physiol.

[18] Schoor M, Schuster-Gossler K, Roopenian D, Gossler A (1999). Skeletal dysplasias, growth retardation, reduced postnatal survival, and impaired fertility in mice lacking the SNF2/SWI2 family member ETL1.. Mech Dev.

[19] Ueda T, Watanabe-Fukunaga R, Ogawa H, Fukuyama H, Higashi Y (2007). Critical role of the p400/mDomino chromatin-remodeling ATPase in embryonic hematopoiesis.. Genes Cells.

[20] Bultman S, Gebuhr T, Yee D, La Mantia C, Nicholson J (2000). A Brg1 null mutation in the mouse reveals functional differences among mammalian SWI/SNF complexes.. Mol Cell.

[21] Reyes JC, Barra J, Muchardt C, Camus A, Babinet C (1998). Altered control of cellular proliferation in the absence of mammalian brahma (SNF2alpha).. Embo J.

[22] Roberts CW, Orkin SH (2004). The SWI/SNF complex—chromatin and cancer.. Nat Rev Cancer.

[23] Stopka T, Skoultchi AI (2003). The ISWI ATPase Snf2h is required for early mouse development.. Proc Natl Acad Sci U S A.

[24] Fyodorov DV, Blower MD, Karpen GH, Kadonaga JT (2004). Acf1 confers unique activities to ACF/CHRAC and promotes the formation rather than disruption of chromatin in vivo.. Genes Dev.

[25] Jones MH, Hamana N, Shimane M (2000). Identification and characterization of BPTF, a novel bromodomain transcription factor.. Genomics.

[26] Stryke D, Kawamoto M, Huang CC, Johns SJ, King LA (2003). BayGenomics: a resource of insertional mutations in mouse embryonic stem cells.. Nucleic Acids Res.

[27] Wang QT, Piotrowska K, Ciemerych MA, Milenkovic L, Scott MP (2004). A genome-wide study of gene activity reveals developmental signaling pathways in the preimplantation mouse embryo.. Dev Cell.

[28] Hamatani T, Carter MG, Sharov AA, Ko MS (2004). Dynamics of global gene expression changes during mouse preimplantation development.. Dev Cell.

[29] Tam PP, Loebel DA (2007). Gene function in mouse embryogenesis: get set for gastrulation.. Nat Rev Genet.

[30] Chazaud C, Yamanaka Y, Pawson T, Rossant J (2006). Early lineage segregation between epiblast and primitive endoderm in mouse blastocysts through the Grb2-MAPK pathway.. Dev Cell.

[31] Perea-Gomez A, Vella FD, Shawlot W, Oulad-Abdelghani M, Chazaud C (2002). Nodal antagonists in the anterior visceral endoderm prevent the formation of multiple primitive streaks.. Dev Cell.

[32] Chambers I, Colby D, Robertson M, Nichols J, Lee S (2003). Functional expression cloning of Nanog, a pluripotency sustaining factor in embryonic stem cells.. Cell.

[33] Thomas PQ, Brown A, Beddington RS (1998). Hex: a homeobox gene revealing peri-implantation asymmetry in the mouse embryo and an early transient marker of endothelial cell precursors.. Development.

[34] Varlet I, Collignon J, Robertson EJ (1997). Nodal expression in the primitive endoderm is required for specification of the anterior axis during mouse gastrulation.. Development.

[35] Morrisey EE, Tang Z, Sigrist K, Lu MM, Jiang F (1998). GATA6 regulates HNF4 and is required for differentiation of visceral endoderm in the mouse embryo.. Genes Dev.

[36] Takaoka K, Yamamoto M, Shiratori H, Meno C, Rossant J (2006). The mouse embryo autonomously acquires anterior-posterior polarity at implantation.. Dev Cell.

[37] Perea-Gomez A, Lawson KA, Rhinn M, Zakin L, Brulet P (2001). Otx2 is required for visceral endoderm movement and for the restriction of posterior signals in the epiblast of the mouse embryo.. Development.

[38] Mesnard D, Guzman-Ayala M, Constam DB (2006). Nodal specifies embryonic visceral endoderm and sustains pluripotent cells in the epiblast before overt axial patterning.. Development.

[39] Ding J, Yang L, Yan YT, Chen A, Desai N (1998). Cripto is required for correct orientation of the anterior-posterior axis in the mouse embryo.. Nature.

[40] Blum M, Gaunt SJ, Cho KW, Steinbeisser H, Blumberg B (1992). Gastrulation in the mouse: the role of the homeobox gene goosecoid.. Cell.

[41] Perea-Gomez A, Shawlot W, Sasaki H, Behringer RR, Ang S (1999). HNF3beta and Lim1 interact in the visceral endoderm to regulate primitive streak formation and anterior-posterior polarity in the mouse embryo.. Development.

[42] Sun X, Meyers EN, Lewandoski M, Martin GR (1999). Targeted disruption of Fgf8 causes failure of cell migration in the gastrulating mouse embryo.. Genes Dev.

[43] Winnier G, Blessing M, Labosky PA, Hogan BL (1995). Bone morphogenetic protein-4 is required for mesoderm formation and patterning in the mouse.. Genes Dev.

[44] Arman E, Haffner-Krausz R, Chen Y, Heath JK, Lonai P (1998). Targeted disruption of fibroblast growth factor (FGF) receptor 2 suggests a role for FGF signaling in pregastrulation mammalian development.. Proc Natl Acad Sci U S A.

[45] Rossant J, Guillemot F, Tanaka M, Latham K, Gertenstein M (1998). Mash2 is expressed in oogenesis and preimplantation development but is not required for blastocyst formation.. Mech Dev.

[46] Shalaby F, Rossant J, Yamaguchi TP, Gertsenstein M, Wu XF (1995). Failure of blood-island formation and vasculogenesis in Flk-1-deficient mice.. Nature.

[47] Miquerol L, Gertsenstein M, Harpal K, Rossant J, Nagy A (1999). Multiple developmental roles of VEGF suggested by a LacZ-tagged allele.. Dev Biol.

[48] Shaulian E, Karin M (2002). AP-1 as a regulator of cell life and death.. Nat Cell Biol.

[49] Gadue P, Huber TL, Paddison PJ, Keller GM (2006). Wnt and TGF-beta signaling are required for the induction of an in vitro model of primitive streak formation using embryonic stem cells.. Proc Natl Acad Sci U S A.

[50] Brennan J, Lu CC, Norris DP, Rodriguez TA, Beddington RS (2001). Nodal signalling in the epiblast patterns the early mouse embryo.. Nature.

[51] Derynck R, Zhang YE (2003). Smad-dependent and Smad-independent pathways in TGF-beta family signalling.. Nature.

[52] Massague J, Seoane J, Wotton D (2005). Smad transcription factors.. Genes Dev.

[53] Jonk LJ, Itoh S, Heldin CH, ten Dijke P, Kruijer W (1998). Identification and functional characterization of a Smad binding element (SBE) in the JunB promoter that acts as a transforming growth factor-beta, activin, and bone morphogenetic protein-inducible enhancer.. J Biol Chem.

[54] Kumar A, Novoselov V, Celeste AJ, Wolfman NM, ten Dijke P (2001). Nodal signaling uses activin and transforming growth factor-beta receptor-regulated Smads.. J Biol Chem.

[55] Yu L, Hebert MC, Zhang YE (2002). TGF-beta receptor-activated p38 MAP kinase mediates Smad-independent TGF-beta responses.. Embo J.

[56] Santner SJ, Dawson PJ, Tait L, Soule HD, Eliason J (2001). Malignant MCF10CA1 cell lines derived from premalignant human breast epithelial MCF10AT cells.. Breast Cancer Res Treat.

[57] van Grunsven LA, Verstappen G, Huylebroeck D, Verschueren K (2005). Smads and chromatin modulation.. Cytokine Growth Factor Rev.

[58] Yashiro K, Saijoh Y, Sakuma R, Tada M, Tomita N (2000). Distinct transcriptional regulation and phylogenetic divergence of human LEFTY genes.. Genes Cells.

[59] Cheng AM, Saxton TM, Sakai R, Kulkarni S, Mbamalu G (1998). Mammalian Grb2 regulates multiple steps in embryonic development and malignant transformation.. Cell.

[60] Feldman B, Poueymirou W, Papaioannou VE, DeChiara TM, Goldfarb M (1995). Requirement of FGF-4 for postimplantation mouse development.. Science.

[61] Huelsken J, Vogel R, Brinkmann V, Erdmann B, Birchmeier C (2000). Requirement for beta-catenin in anterior-posterior axis formation in mice.. J Cell Biol.

[62] Liu P, Wakamiya M, Shea MJ, Albrecht U, Behringer RR (1999). Requirement for Wnt3 in vertebrate axis formation.. Nat Genet.

[63] Waldrip WR, Bikoff EK, Hoodless PA, Wrana JL, Robertson EJ (1998). Smad2 signaling in extraembryonic tissues determines anterior-posterior polarity of the early mouse embryo.. Cell.

[64] Xi R, Xie T (2005). Stem cell self-renewal controlled by chromatin remodeling factors.. Science.

[65] Lee EC, Yu D, Martinez de Velasco J, Tessarollo L, Swing DA (2001). A highly efficient Escherichia coli-based chromosome engineering system adapted for recombinogenic targeting and subcloning of BAC DNA.. Genomics.

[66] Tessarollo L (2001). Manipulating mouse embryonic stem cells.. Methods Mol Biol.

[67] Nagy A (2003). Manipulating the mouse embryo: A laboratory manual.

[68] Carter MG, Sharov AA, VanBuren V, Dudekula DB, Carmack CE (2005). Transcript copy number estimation using a mouse whole-genome oligonucleotide microarray.. Genome Biol.

[69] Sharov AA, Dudekula DB, Ko MS (2005). A web-based tool for principal component and significance analysis of microarray data.. Bioinformatics.

[70] Sharov AA, Dudekula DB, Ko MS (2005). Genome-wide assembly and analysis of alternative transcripts in mouse.. Genome Res.

[71] Ausubel FM (1987). Current protocols in molecular biology.

