# Supplementary figures and images for: Essential Role of Chromatin Remodeling Protein Bptf in Early Mouse Embryos and Embryonic Stem Cells
Source: PLoS Genet. 2008 Oct 31;4(10):e1000241. doi: 10.1371/journal.pgen.1000241 (PMC2570622; doi:10.1371/journal.pgen.1000241)

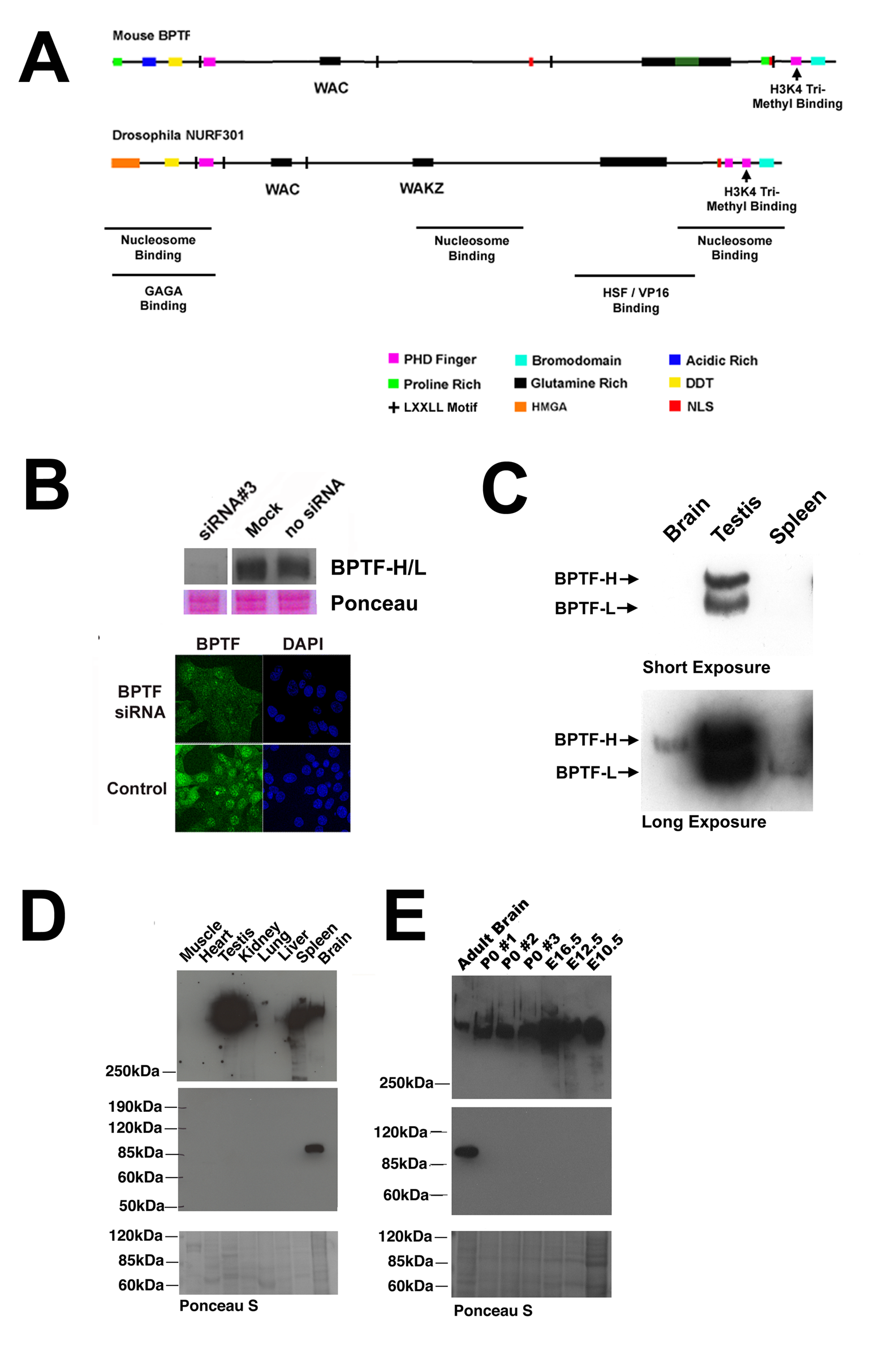

Supplement: Figure S1 — Bptf is the homolog of NURF 301. (A) Mouse Bptf is highly homologous to Drosophila NURF301. Major functional domains are conserved between mammals and flies, including nucleosome and transcription factor binding sites, strongly suggesting that Bptf is the homolog of the largest subunit of the Drosophila NURF complex, NURF301. (B) Bptf is predominantly nuclear in cultured P19 cells. siRNA targeting of Bptf results in decreased Bptf signal by Western analysis and a reduction of nuclear fluorescence by anti-Bptf immuno fluorescence in P19 cells. (C) Western analysis of adult brain, testis, spleen, and embryonic stem cells reveals two migrating species, Bptf-H and Bptf-L. (D) Western analysis of total protein extracts from adult tissues using the affinity purified anti-Bptf antisera identifies a single band of high molecular weight predominately found in testis, spleen, and brain. In addition to full length Bptf, we identify a single ∼100 kDa protein which reacts with the antibody exclusively in the adult brain. This protein is identical in size to the previously reported Bptf splice variant FAC1 (Bowser R, Giambrone A, Daves P (1995) Dev Neuroscience 17: 20–37.). (E) A similar western analysis of protein extracts from total embryo proteins identifies high Bptf expression throughout development. The ∼100 kDa cross-reacting band was only found in the adult brain loading standard and not during development. (1.28 MB TIF) [file pgen.1000241.s001.tif]

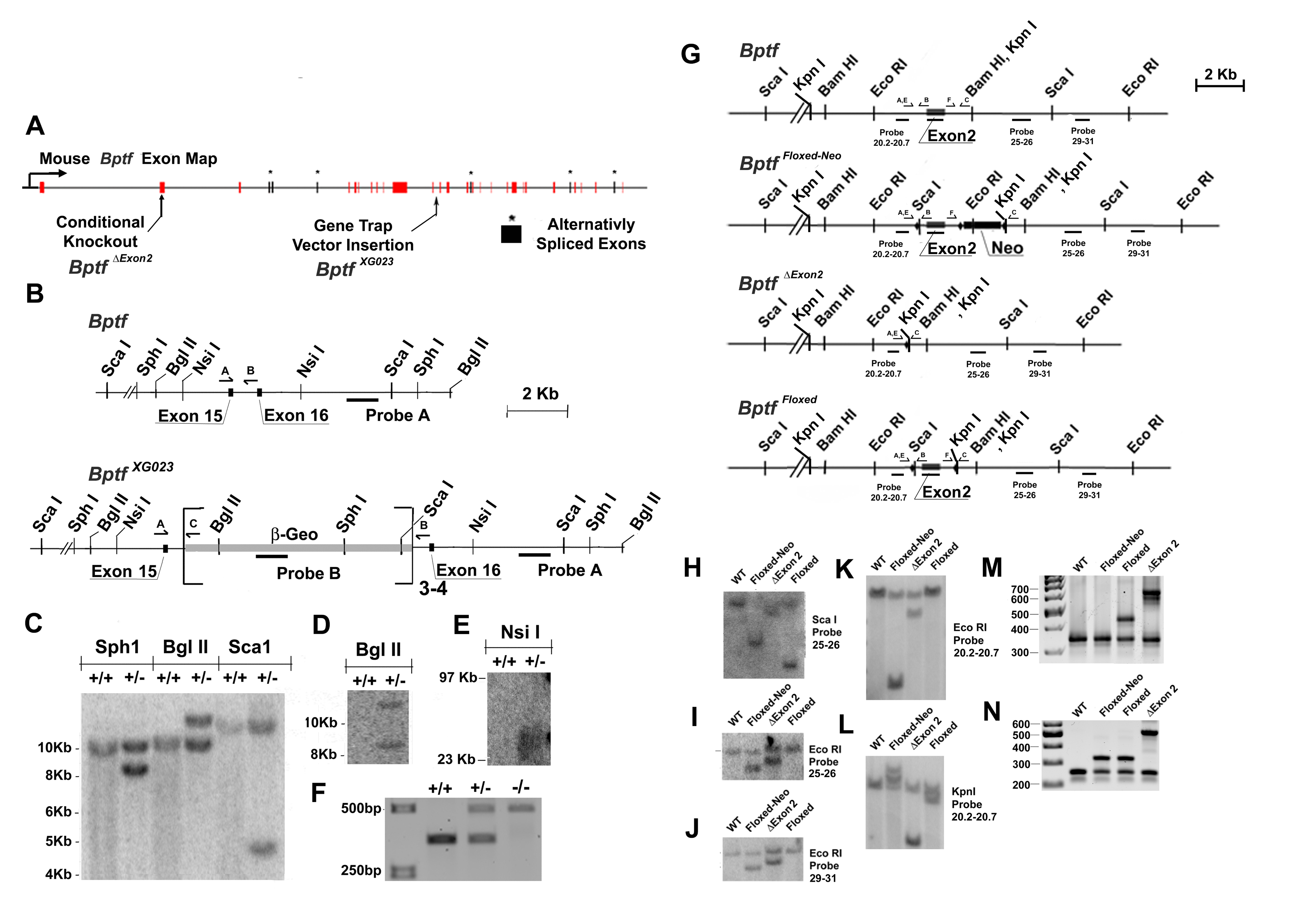

Supplement: Figure S2 — Generation of conditional and gene trap alleles of Bptf. (A) Intron-exon map of mouse Bptf showing alternatively spliced exons and the position of the BptfXG023 trapping vector and BptfΔExon2 floxed exon. (B) Diagram of Bptf and BptfXG023 alleles showing tandem trapping vector insertion site between exons 15 and 16. Restriction enzyme sites and positions of Southern blotting probes used for the characterization of BptfXG023 are shown as external probe A and internal probe B. Positions of primers used in multiplex PCR genotyping are designated A, B, and C with A and B complementing genomic sequence spanning the trapping vector insertion site and C the trapping vector. (C) A Southern blot using probe A showing diagnostic restriction enzyme sites confirming the insertion of the trapping vector into Bptf. (D) A Southern blot using Bgl II and probe B showing two bands characteristic of multiple integration events by the trapping vector. Upper band contains sequences external and lower band internal to the trapping vector. (E) A Southern blot of genomic DNA digested with Nsi I, resolved by FIGE and blotted with probe B. A single band was identified in heterozygous mice with an approximate size of 30 Kb, suggesting 3 to 4 trapping vector integration events. (F) Results from multiplex PCR analysis of wild type (+/+), heterozygous (+/-), and homozygous (−/−) BptfXG023 E7.5 embryos using primers A, B, and C. The wild type and mutant alleles are identified by a 325 bp and 500 bp product, respectively. (G) Diagram of Bptf, BptfFloxed-Neo, BptfFloxed, and BptfΔExon2 alleles showing the configuration of the integrated targeting vector and its Cre and Flp reaction products. Restriction enzyme sites and positions of Southern blotting probes used for the characterization of BptfΔExon2 are shown as external probe 29–31 and internal probes 20.2–20.7, exon2 and 25–26. Positions of primers used in multiplex PCR genotyping are designated A, B, C, E, and F. (H) A Southern blot of heterozy [file pgen.1000241.s002.tif]

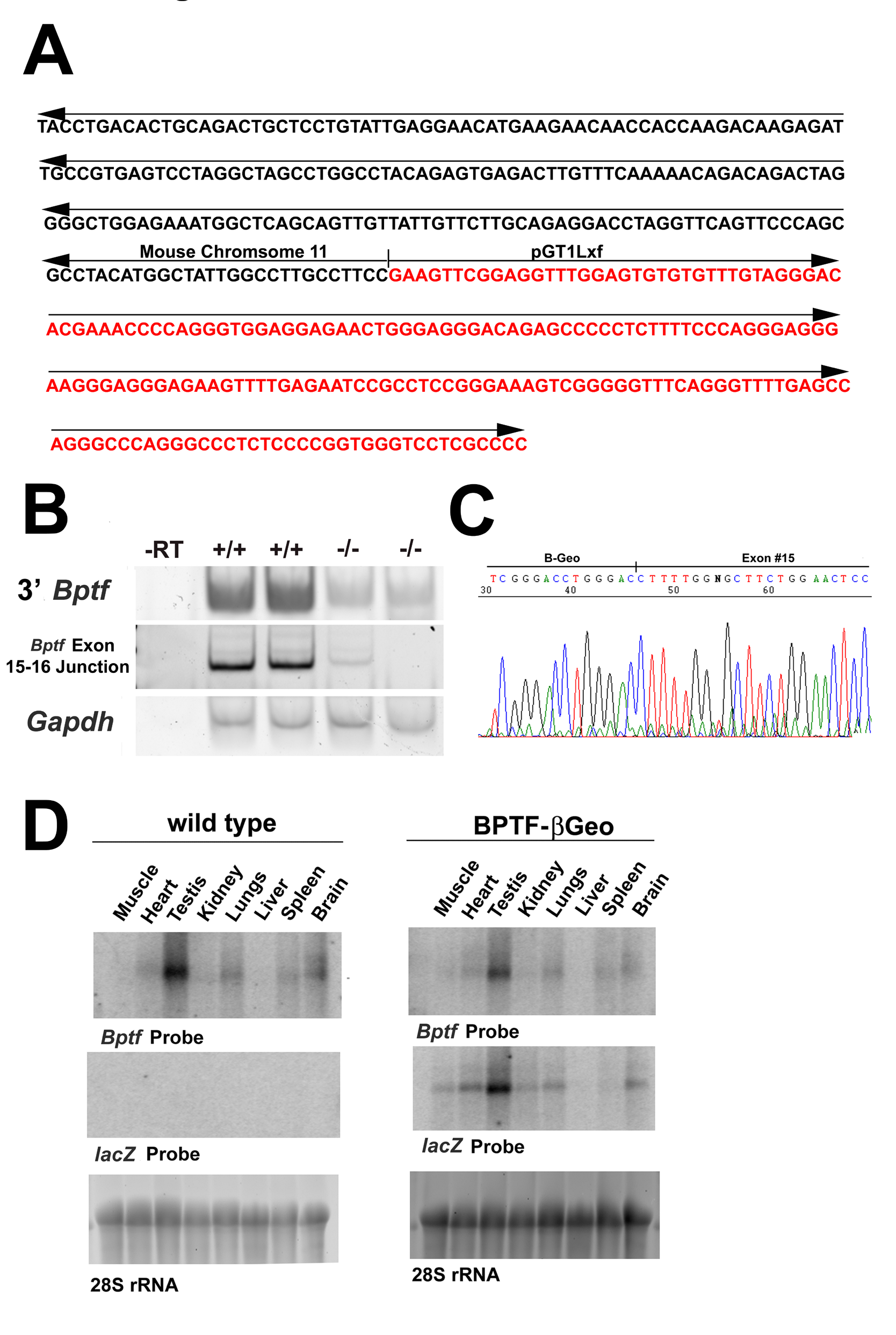

Supplement: Figure S3 — Molecular characterization of BptfXG023 allele. (A) PCR amplification and sequencing of the upstream junction between the trapping vector and the mouse genome confirm the exact position of the trapping vector to be between exon 15 and 16 of Bptf. (B) RT-PCR results from E7.5 embryos show reduced expression of 3′-RNA sequences and severely reduced exon 15–16 splicing, confirming efficient trapping of Bptf by the trapping vector insertion. (C) DNA sequence of a 5′-RACE amplicon of the BptfXG023 allele from testis shows an in-frame fusion between exon 15 of Bptf and β-Geo from the trapping vector. (D) Northern analysis of RNA from wild type and heterozygous BptfXG023 disruption mice show predominant transcripts in testis, lungs, spleen, and brain using a probe specific for Bptf mRNA. The Bptf-β-Geo fusion RNA has the same expression patterns as the wild type allele, suggesting that the trapping vector does not interfere with Bptf regulation at the level of transcription. (1.34 MB TIF) [file pgen.1000241.s003.tif]

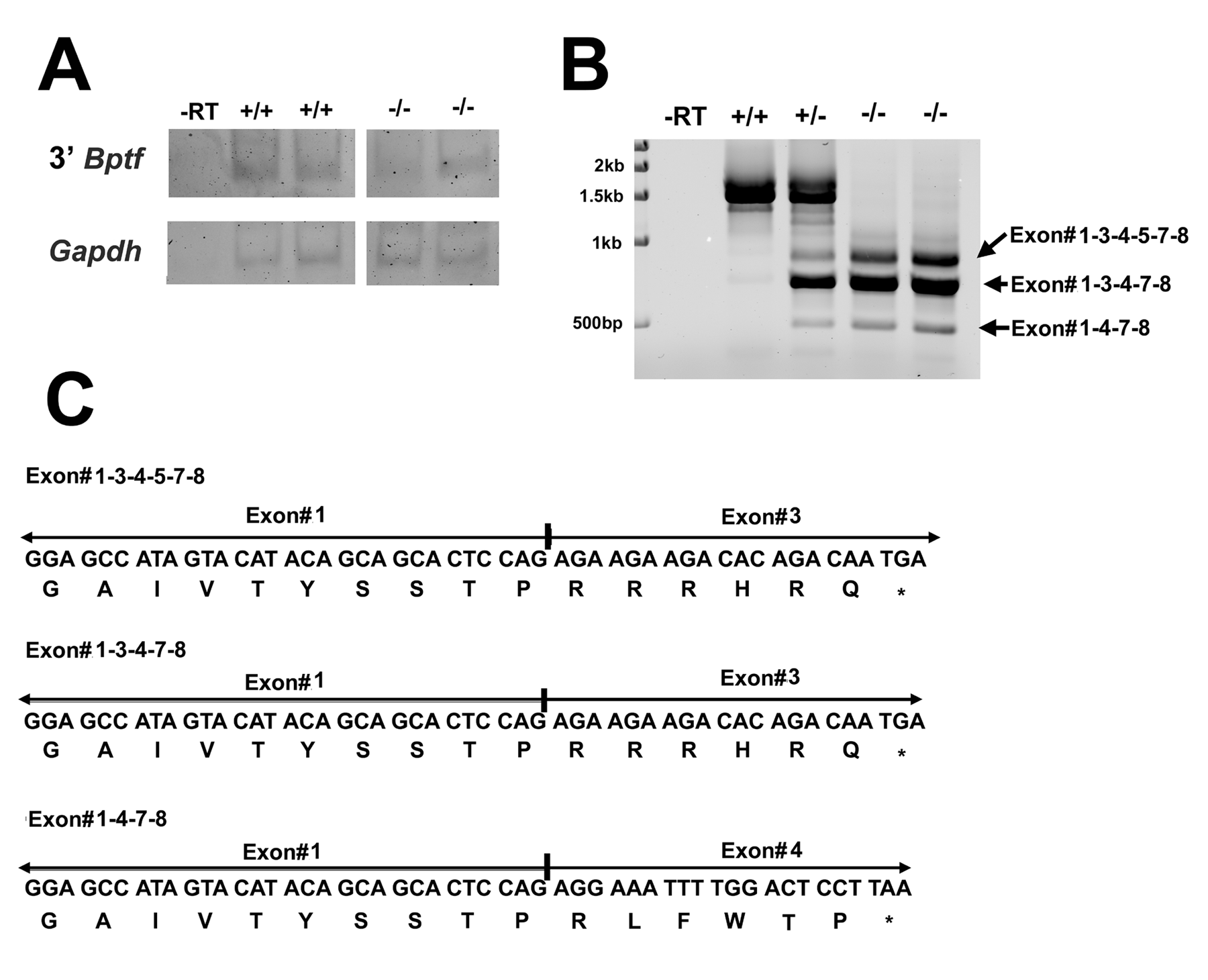

Supplement: Figure S4 — Molecular characterization of BptfΔExon2 allele. (A) RT-PCR results from E7.5 embryos show slightly reduced expression of 3′-RNA sequences of BptfΔExon2 at the RNA level. (B) RT-PCR results from E7.5 embryos show deletion of exon 2 RNA sequences from BptfΔExon2 transcripts. Primers used were complementary to exons 1 and 8. (C) RT-PCR products from BptfΔExon2 transcripts in (B) were sequenced to identify out of frame splicing events, strongly suggesting that the exon 2 deletion results in efficient knockout of the Bptf protein. (0.64 MB TIF) [file pgen.1000241.s004.tif]

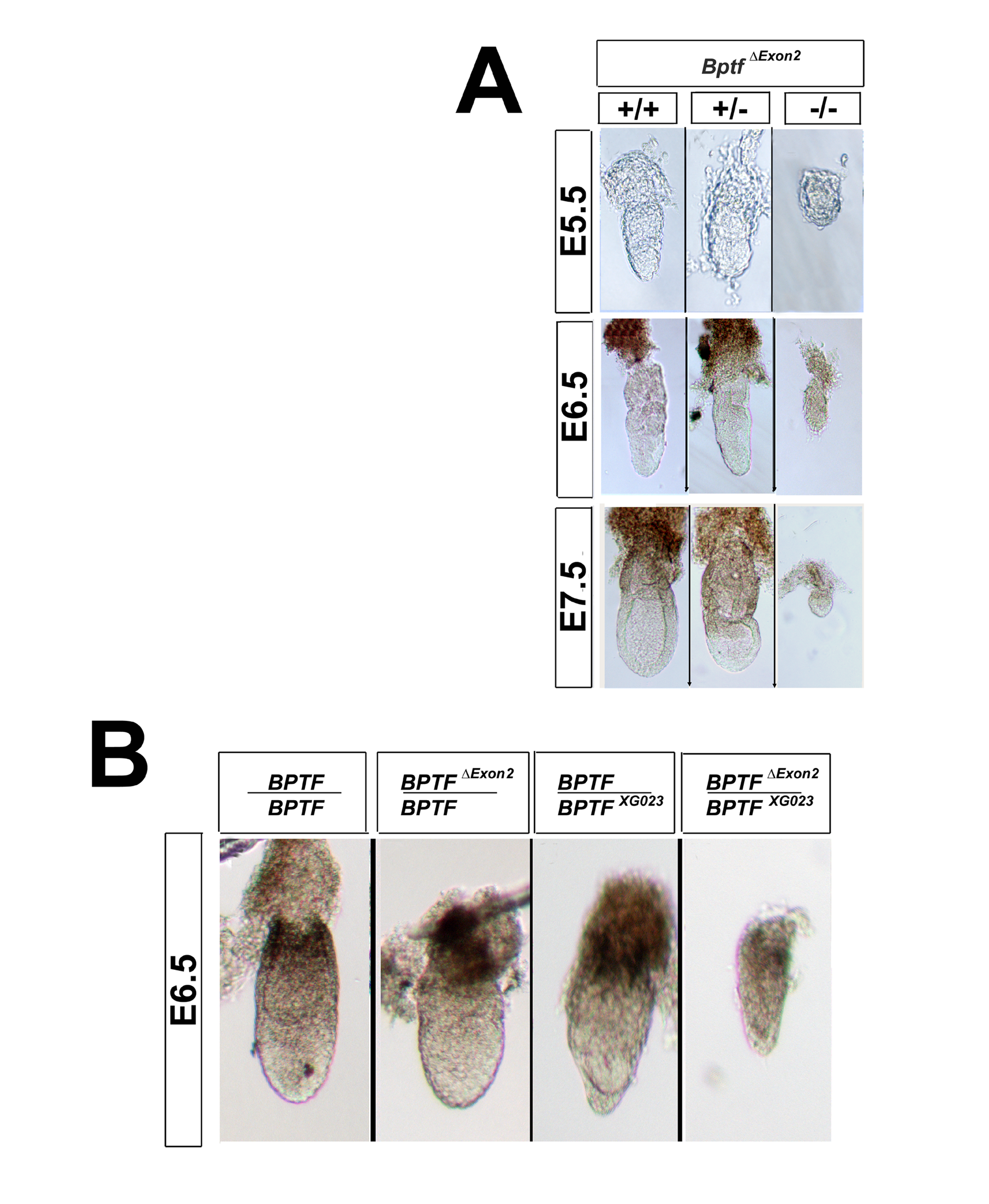

Supplement: Figure S5 — BptfΔExon2 and BptfXG023 alleles are genetically equivalent. (A) Wild type (+/+), heterozygous (+/−), and homozygous (−/−) BptfΔExon2 embryos at E5.5, E6.5, and E7.5 were removed from their decedua and genotyped using a PCR-based method. Identical to the BptfXG023 allele, reduced growth rates are evident in the homozygous BptfΔExon2 embryos as early as E5.5. Mutant embryos from both lines maintained the gross morphology of E5.5 embryos at E6.5 and E7.5 with an overall increase in size. (B) Heterozygous BptfXG023 and BptfΔExon2 mice were intercrossed, and wild type, BptfΔExon2 heterozygous, BptfXG023 heterozygous, and BptfXG023 / BptfΔExon2 trans-heterozygous embryos were dissected from their decidua at E6.5, and genotypes were confirmed by PCR. Trans-heterozygous embryos phenocopy homozygous BptfXG023 or BptfΔExon2 embryos, suggesting that the two alleles have an equivalent phenotype. (3.04 MB TIF) [file pgen.1000241.s005.tif]

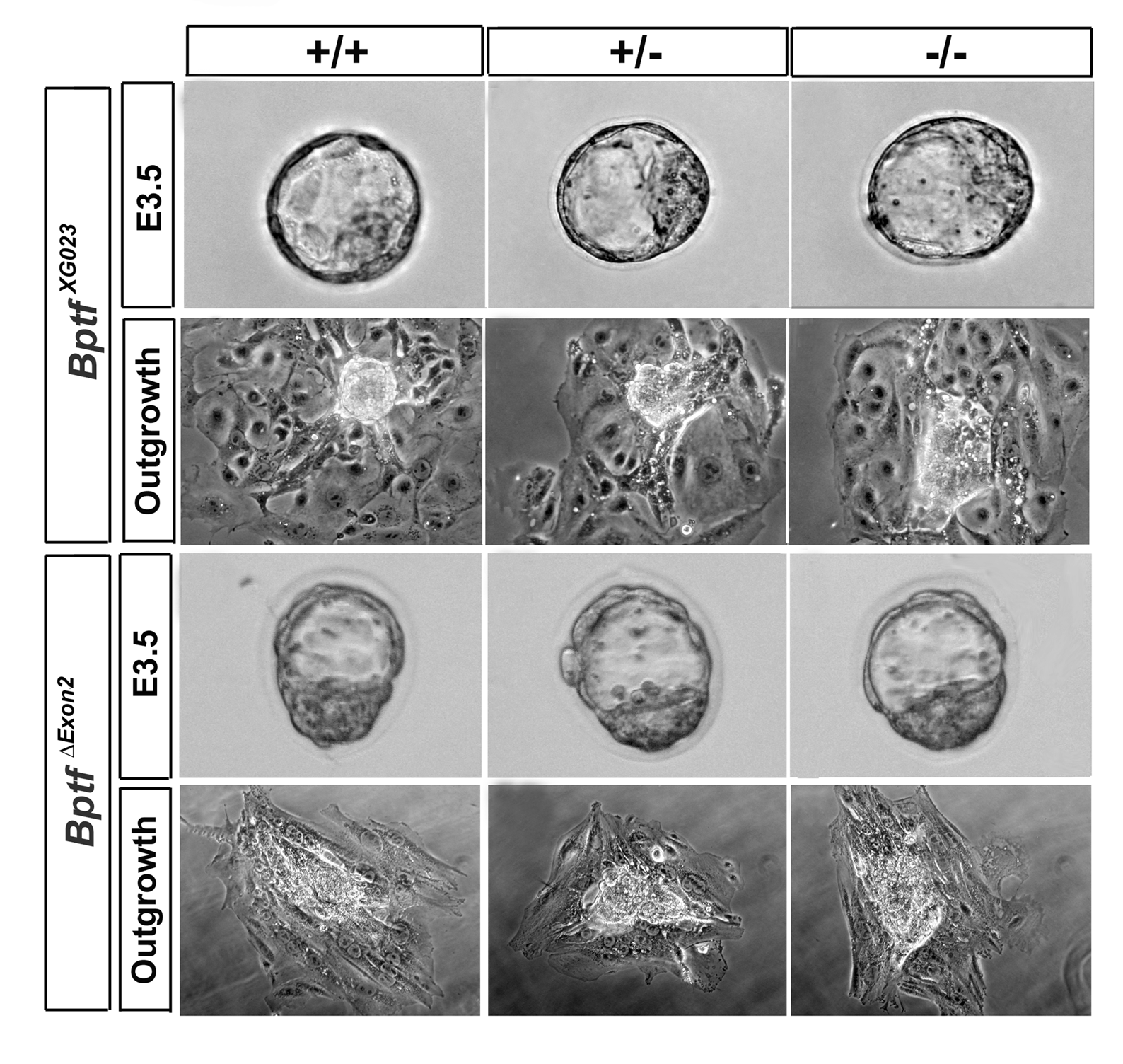

Supplement: Figure S6 — Bptf mutants do not have significant growth defects prior to implantation. Wild type (+/+), heterozygous (+/−), and homozygous (−/−) BptfXG023 or BptfΔExon2 E3.5 blastocysts and 5-day blastocyst outgrowths did not reveal any significant morphological or growth differences in trophoblast or the inner cell mass. (1.36 MB TIF) [file pgen.1000241.s006.tif]

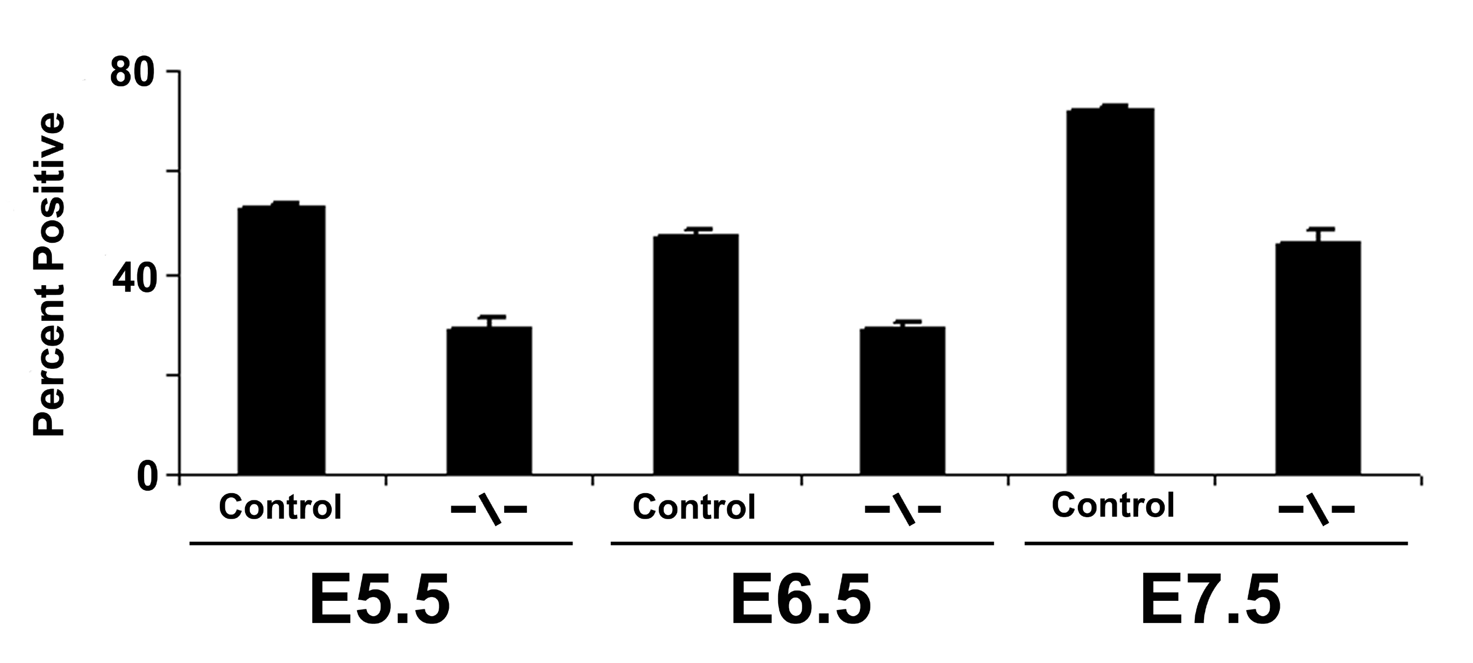

Supplement: Figure S7 — Bptf mutants have reduced cellular proliferation. Percent phosphorylated histone H3 positive cells from total cells of the control and mutant embryos were quantified to show reduced cellular proliferation of Bptf mutant embryos at E5.5, E6.5, and E7.5. (0.15 MB TIF) [file pgen.1000241.s007.tif]

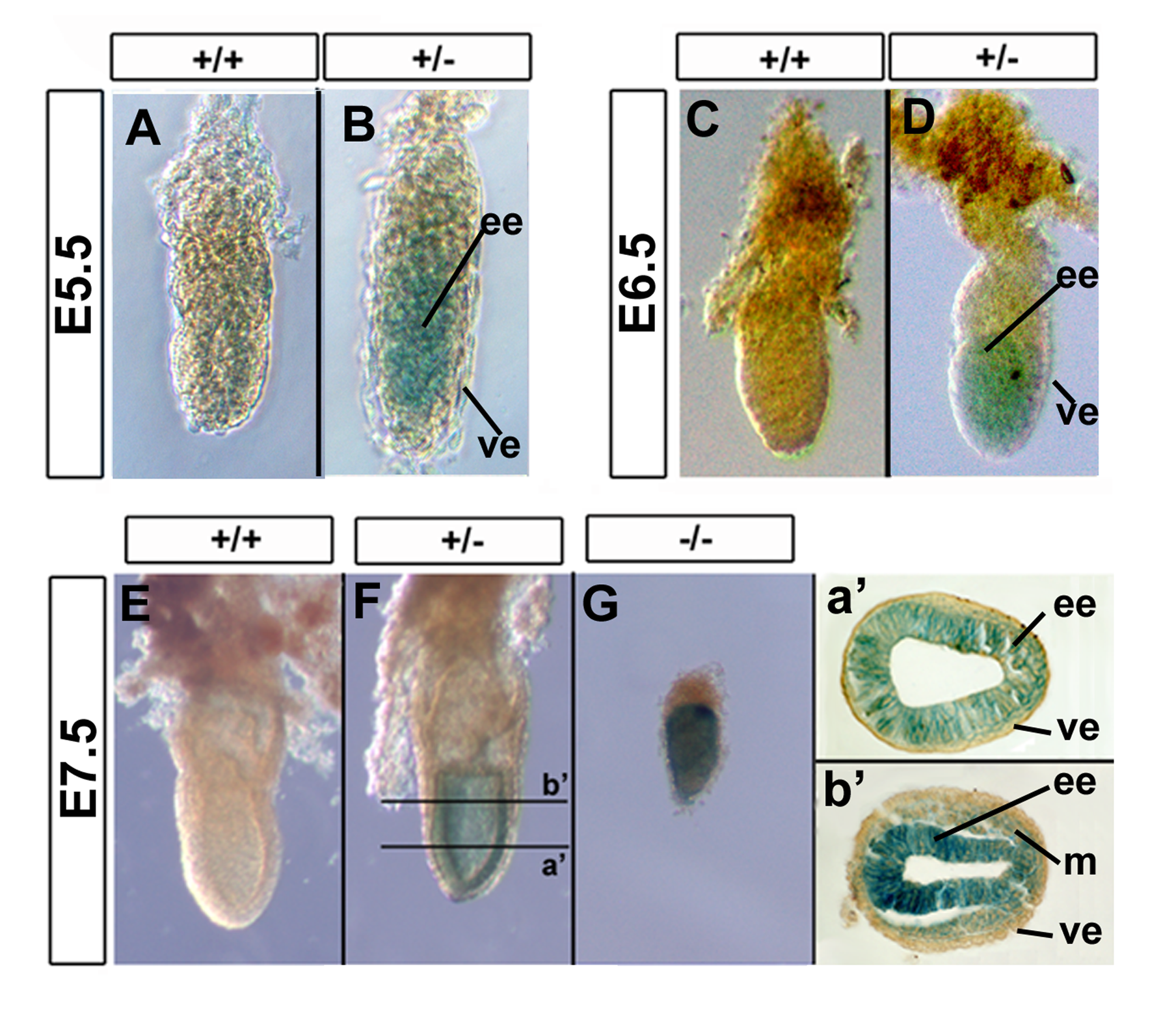

Supplement: Figure S8 — BptfXG023 is widely expressed in the embryo proper. (A–D) Wild type and heterozygous embryos were stained in whole mount for β-galactosidase activity at E5.5 and E6.5. BptfXG023 expression is present in E5.5 and E6.5 embryos in embryonic but not extra-embryonic tissues. (E–G) Wild type and heterozygous E7.5 embryos were stained in whole mount for β-galactosidase activity. BptfXG023 expression is present at E7.5 embryos in embryonic but not extra-embryonic tissues. (a′) Cross section of the E7.5 heterozygous embryo shows expression exclusive to the embryonic ectoderm and little to no expression in the visceral endoderm. (b′) Cross-section of the E7.5 heterozygous embryo shows expression primarily in the embryonic ectoderm, reduced expression in the mesoderm, with little to no expression in the visceral endoderm. Abbreviations: ve, visceral endoderm; ee, embryonic ectoderm; m, mesoderm. (0.34 MB TIF) [file pgen.1000241.s008.tif]

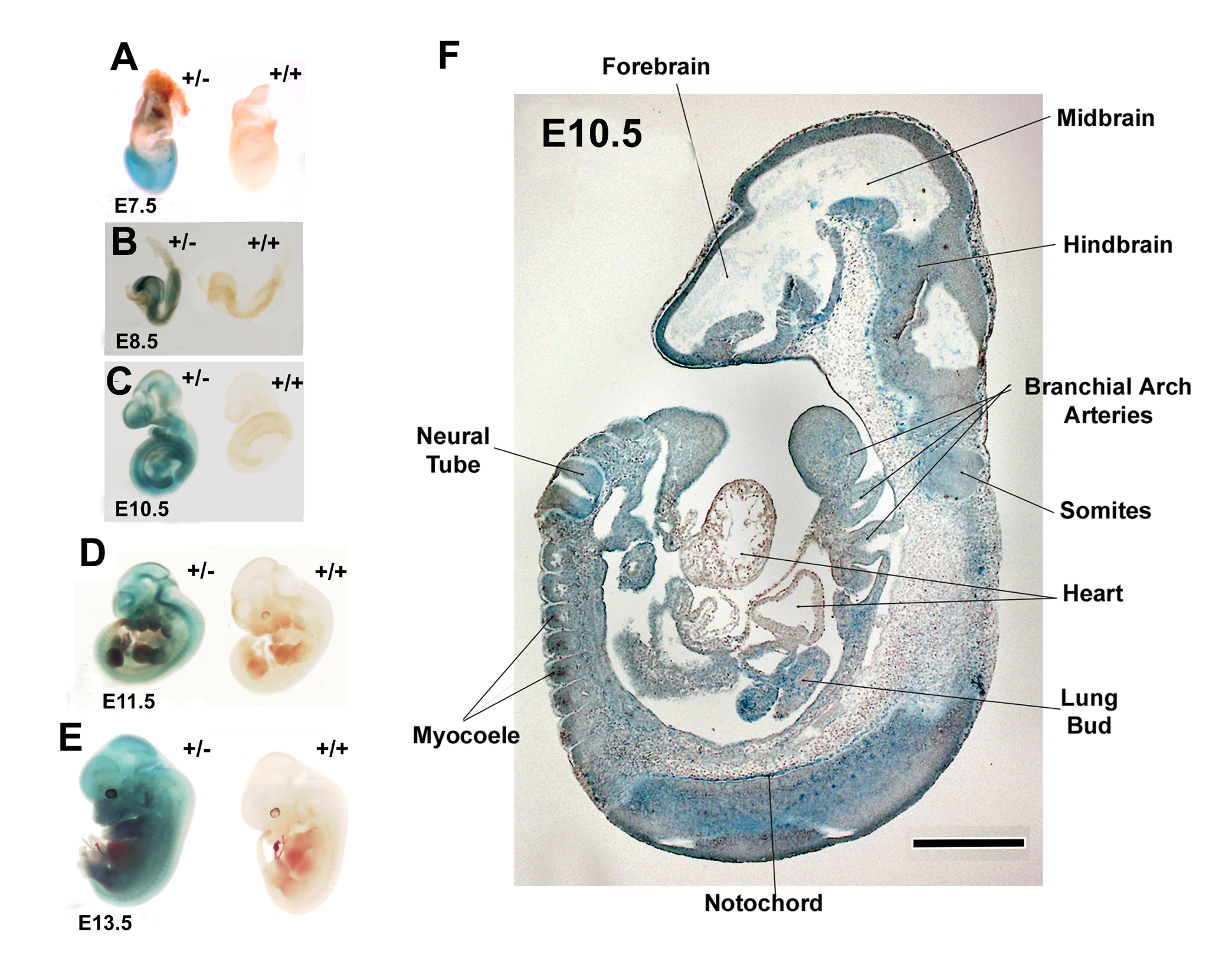

Supplement: Figure S9 — BptfXG023 expression during embryonic development. (A–E) Wild type (+/+) and heterozygous (+/−) BptfXG023 embryos were dissected from their decidua and stained in whole mount for β-galactosidase activity. Staining is seen predominantly in the embryonic tissues at E7.5 (A) and E8.5 (B). At later stages of development, expression is ubiquitous in the embryo (C–E). (F) Sagittal section of E10.5 embryo stained for β-galactosidase activity reveals the extent of BptfXG023 expression. Expression was observed in many tissues originating from mesoderm (notochord, myocoele, somites), endoderm (lung bud), and ectoderm (forebrain, midbrain, hindbrain, and neural tube). Scale Bar = 400 µm. (4.01 MB TIF) [file pgen.1000241.s009.tif]

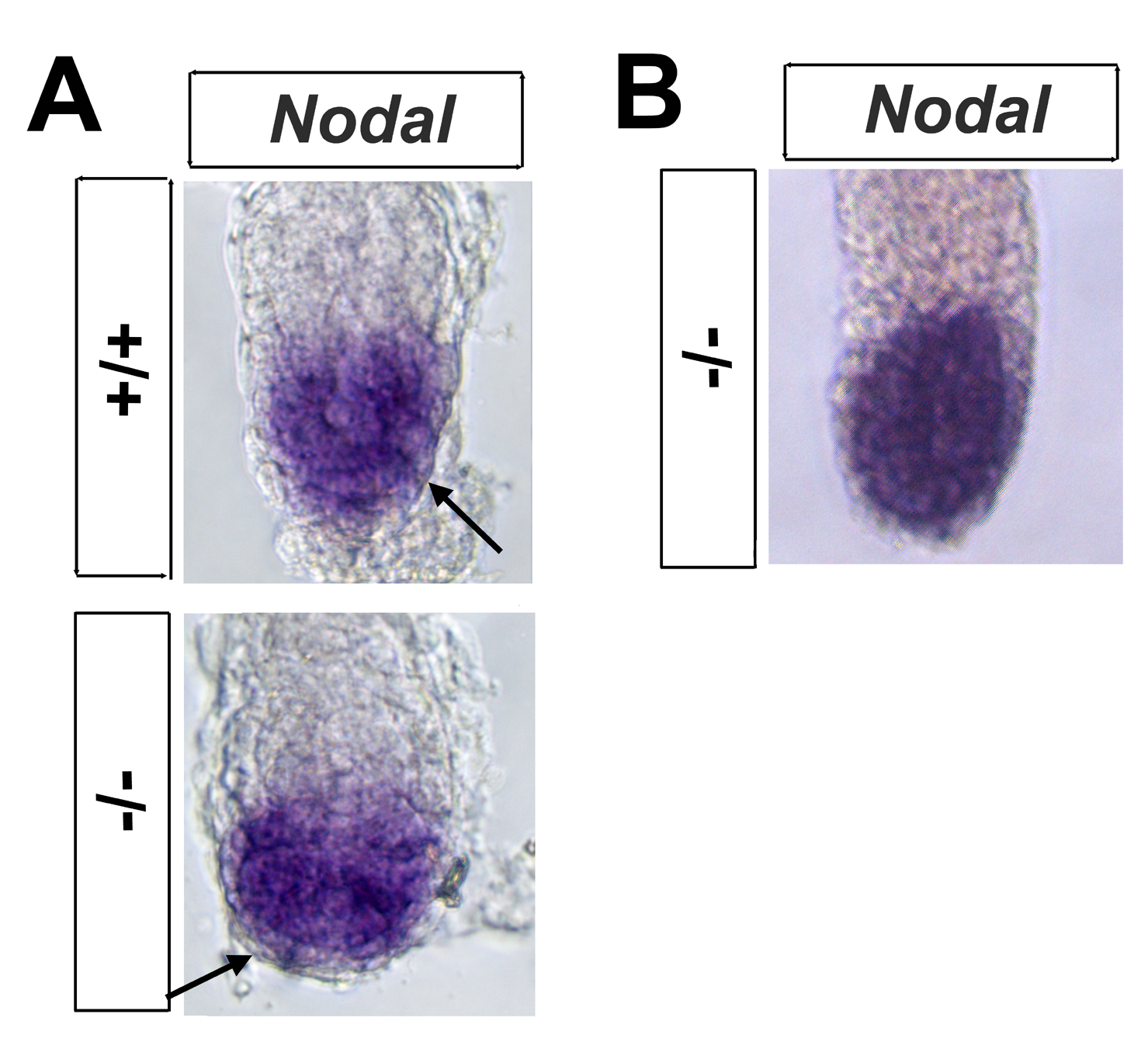

Supplement: Figure S10 — An analysis of Nodal expression at E5.5 shows Bptf mutants express Nodal at levels comparable to wild type. (A) Bptf mutants express Nodal in the visceral endoderm at E5.5 at comparable levels to the wild type control. (B) Bptf mutants express delocalized Nodal at E6.5. (1.94 MB TIF) [file pgen.1000241.s010.tif]

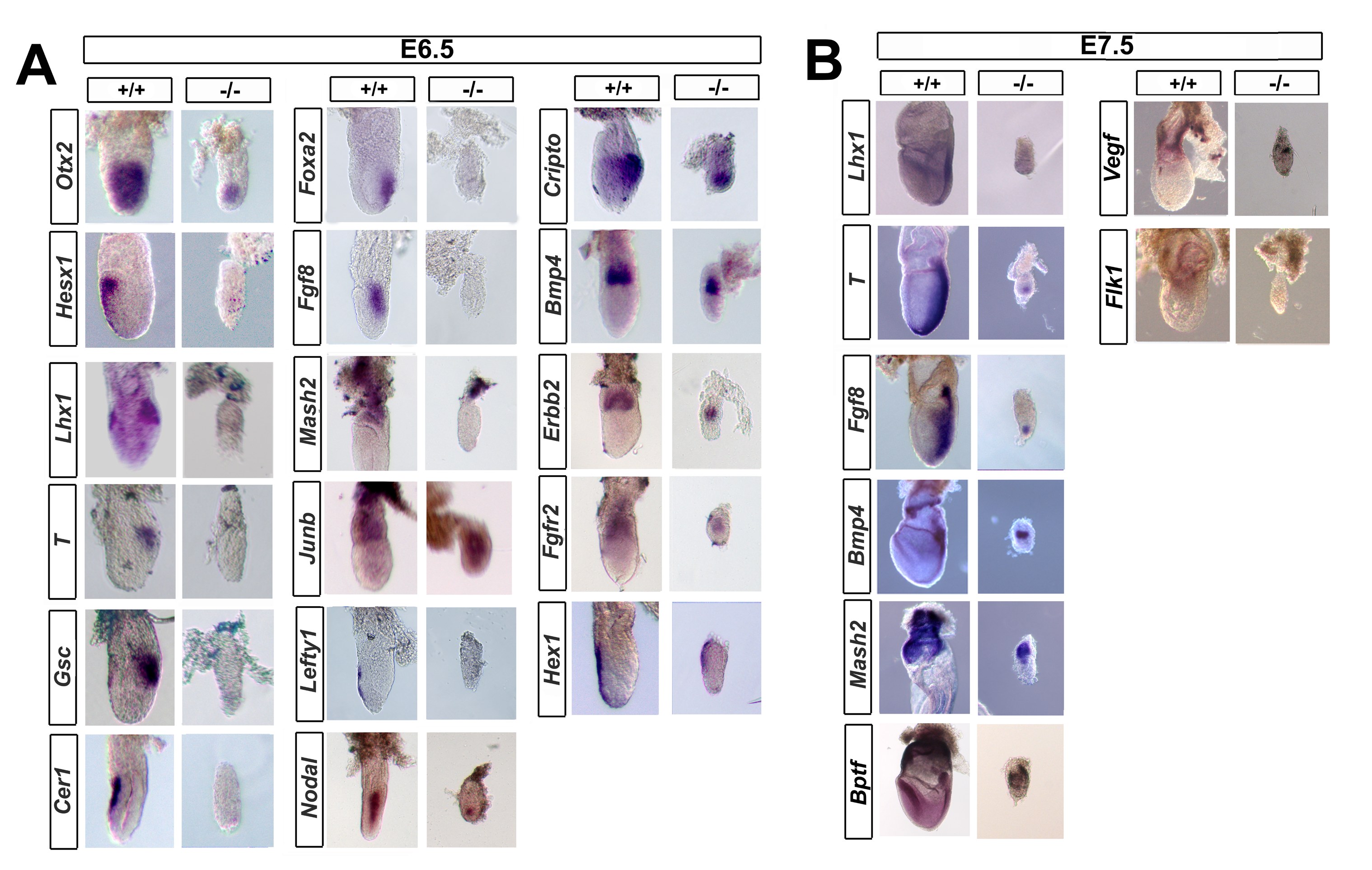

Supplement: Figure S11 — Bptf mutants cannot establish an anterior–posterior axis. (A,B) Whole mount in situ RNA hybridization analysis of wild type and mutant E6.5 and E7.5 embryos for Cer1, Nodal, Lefty1, Hex1, Otx2, Hesx1, Lhx1, T, Gsc, Fgf8, Foxa2, Cripto, Junb, Mash2, Bmp4, Erbb2, and Fgfr2 expression. An analysis on wild type and Bptf mutant E6.5 embryos for anterior visceral endoderm (AVE) markers Cer1, Lefty1, and Hex1 show that mutants do not develop an AVE. Bptf mutants show comparable but delocalized Nodal and Cripto expression at E6.5, further suggesting a defect in the development of the AVE. Embryos showed reduced expression of the embryonic ectoderm marker Otx2 and Hesx1 in mutants compared to wild type controls, suggesting defects in the embryonic ectoderm. Primitive streak markers Lhx1, T, Gsc, Fgf8, and Foxa2 also show a significant reduction in expression in mutant at E6.5 compared to wild type. The extra-embryonic markers Bmp4, Erbb2, Fgfr2, and Mash2 are not dramatically affected in mutant when compared to wild type embryos at E6.5. The cell cycle regulatory gene Junb was found to be over expressed in mutant relative to wild type controls at E6.5. Analysis of E7.5 embryos confirms that Bptf mutants do not establish an anterior–posterior axis. Lhx1 is not significantly expressed at E7.5 in mutant compared to wild type, suggesting that primitive streak does not form. Expression of mesoderm markers T and Fgf8 is diffuse in mutant embryos compared to wild type at E7.5, suggesting that the embryo proper becomes unorganized mesoderm-like tissue. The extra-embryonic markers Bmp4 and Mash2 maintain extra-embryonic expression in E7.5 mutant embryos. Angiogenesis markers Vegf and Flk1 show little expression in the extra embryonic portion of the embryo as compared to wild type controls. However, expression is not associated with an allantois, which is severely underdeveloped in Bptf mutant embryos. Bptf expression is largely confined to the embryonic tissues at E7.5. (6.17 MB TIF) [file pgen.1000241.s011.tif]

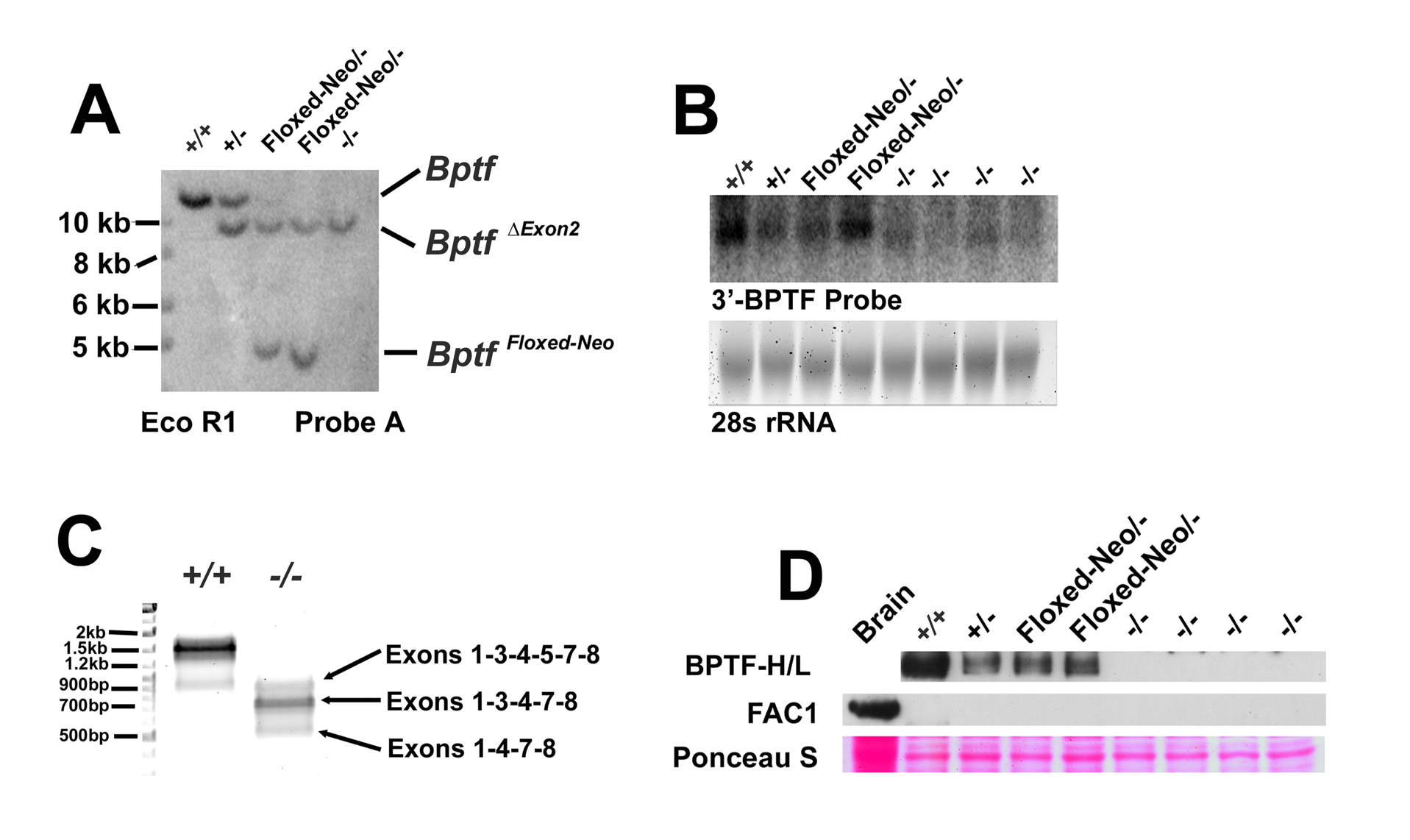

Supplement: Figure S12 — Construction of Bptf mutant embryonic stem (ES) cell lines. (A) Retargeting and subsequent transient Cre expression generated conditional heterozygous (not shown), heterozygous (+/−), conditional homozygous (Floxed-Neo/-), and homozygous (−/−) mutant Bptf mouse ES cell clones. Genotypes were confirmed by Southern analysis using an Eco R1 digest and hybridization with Probe 20.2–20.7. (B) Northern analysis of Bptf-mutant ES cell clones using a probe to the 3′-end of the gene shows reduced Bptf transcript levels. (C) RT-PCR analysis of wild type (+/+) and homozygous mutant (−/−) RNA confirms the complete deletion of exon 2 from Bptf transcripts in homozygous mutant clones. Sequence from Bptf mutant PCR products shows the Bptf transcript contains exon 1–3 and 1–4 out of frame splice events as seen in Figure S4C (data not shown). (D) Western analysis of Bptf wild type, heterozygous, conditional heterozygous, and mutant ES cells. Heterozygosity at Bptf results in reduced Bptf protein levels, raising the possibility of haploinsufficiency phenotypes. Homozygous Bptf ES cells mutants do not show Bptf protein, demonstrating that BptfΔExon2 is likely to be a null allele. FAC1 expression was not found in ES cells. (0.71 MB TIF) [file pgen.1000241.s012.tif]

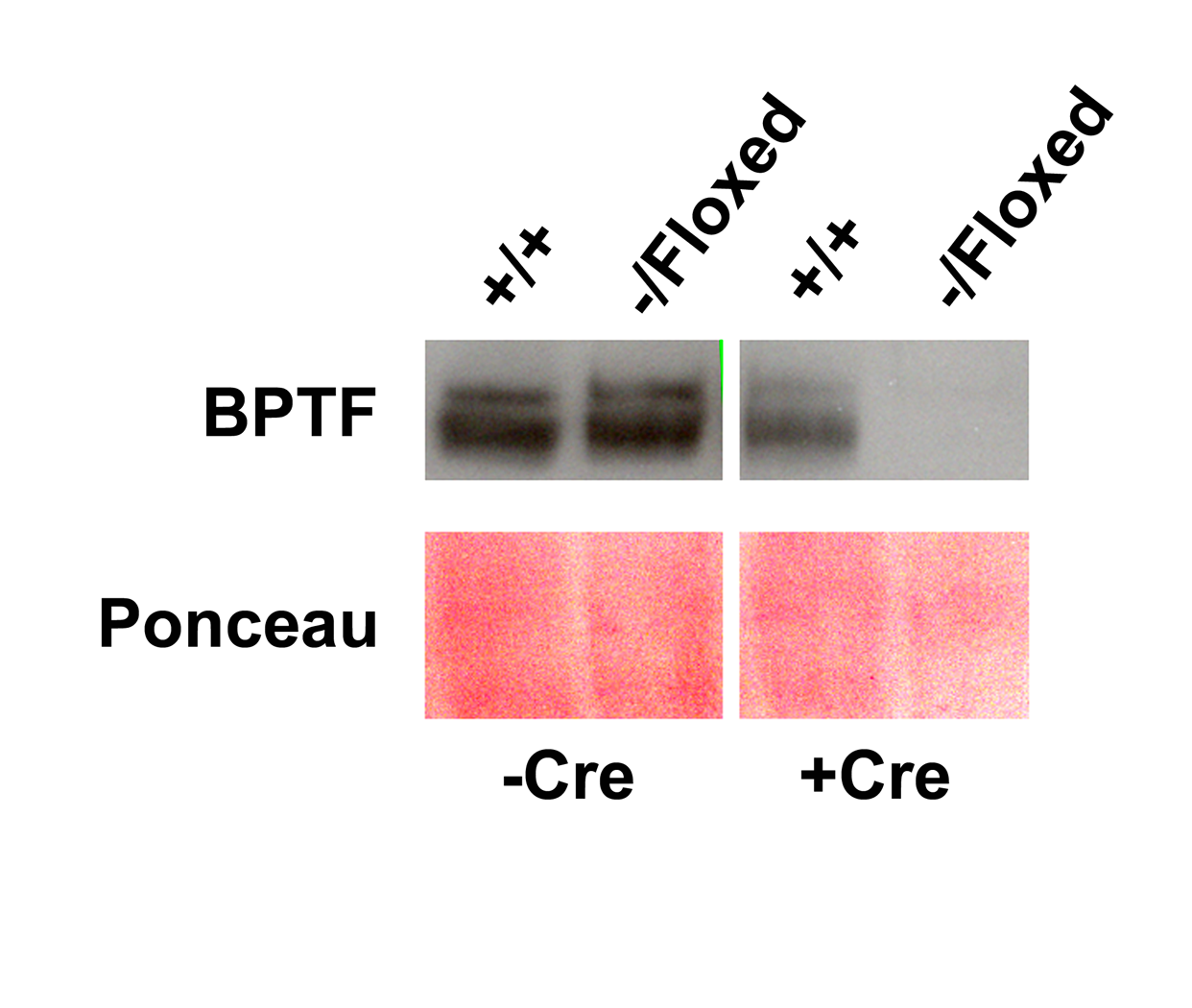

Supplement: Figure S13 — Conditional deletion of Bptf in mouse embryonic fibroblasts (MEF) with adenovirus expressing Cre recombinase. Conditional Bptf mutant (−/Floxed) and control (+/+) MEF lines were infected with an adenovirus expressing the Cre recombinase. Infected (+Cre) and mock infected (−Cre) cells were passaged once to deplete the cellular stores of the Bptf protein. Following growth for 4 days, cells were harvested for total protein. Bptf protein was detected by Western using ponceau S staining as a loading control. (0.57 MB TIF) [file pgen.1000241.s013.tif]

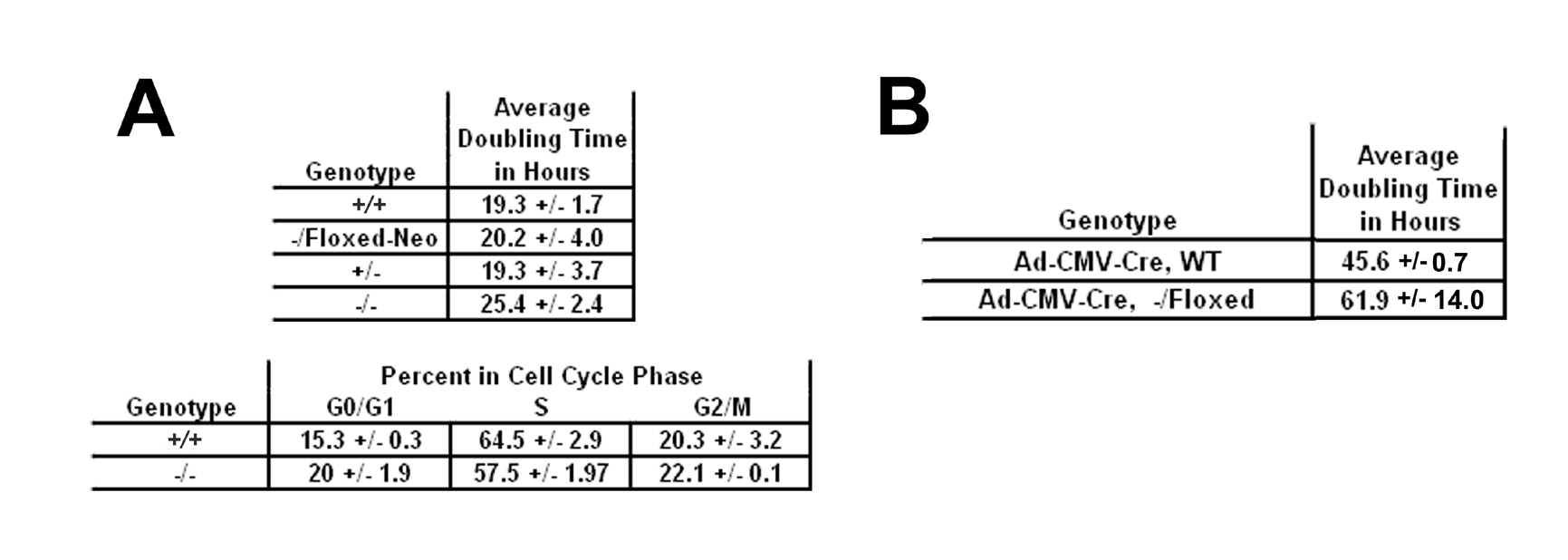

Supplement: Figure S14 — Bptf mutant embryonic stem (ES) and mouse embryonic fibroblast (MEF) cell lines show slight growth defects. (A) The doubling time of wild type and mutant ES cells was investigated. We observed that Bptf mutants but not conditional heterozygous or heterozygous ES cells showed reduced growth rates relative to wild type (+/+) cell lines. A cell cycle analysis of slower growing Bptf knockout ES cell lines shows that they have a slight G1 to S phase delay. (B) The doubling time of wild type and mutant MEF lines was investigated. We observed that Bptf-depleted MEF lines showed slightly reduced growth rates relative to control lines. (0.24 MB TIF) [file pgen.1000241.s014.tif]

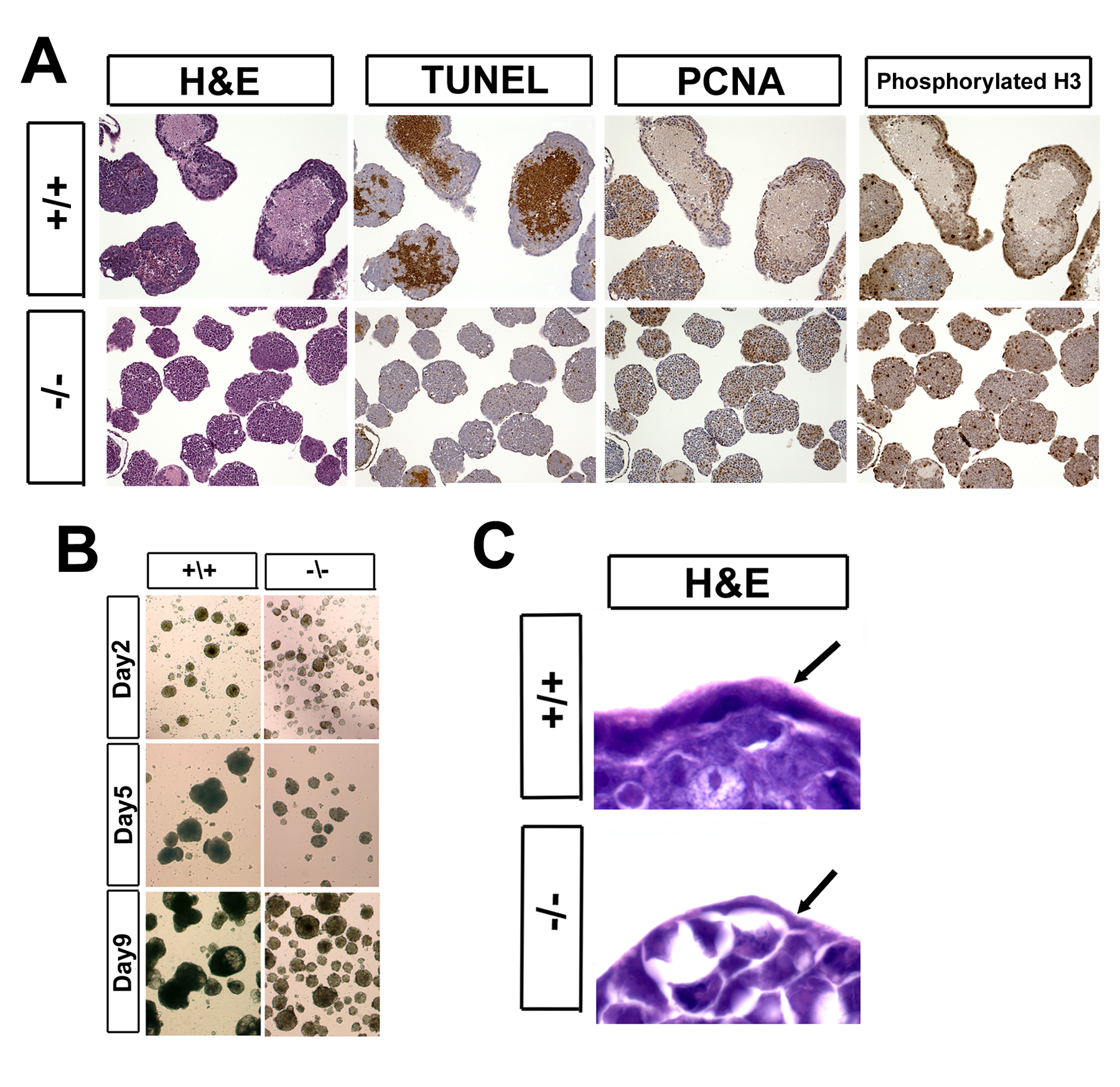

Supplement: Figure S15 — Bptf mutant mouse embryonic stem (ES) cells do not differentiate as embryoid bodies, are not apoptotic, and proliferate normally. (A) Day 9 embryoid bodies were sectioned and stained for gross analysis with hematoxylin and eosin (H&E), the proliferation markers PCNA and phosphorylated histone H3, and apoptosis by TUNEL. Bptf-mutant (−/−) embryoid bodies contained few differentiated and TUNEL positive apoptotic cells compared to wild type (+/+) controls. TUNEL negative Bptf mutant (−/−) and wild type (+/+) cells have similar staining for proliferation markers PCNA and phosphorylated histone H3. (B) Microscopy of wild type and Bptf mutant ES cells during a 9-day embryoid body differentiation time course. Bptf mutant ES cells did not develop the size and cystic structures of the wild type controls. (C) Wild type but not mutant day 9 embryoid bodies develop a prominent layer of endoderm on the exterior surface. In place of endoderm Bptf knockout, embryoid bodies develop a thin epithelial cell layer. (3.46 MB TIF) [file pgen.1000241.s015.tif]

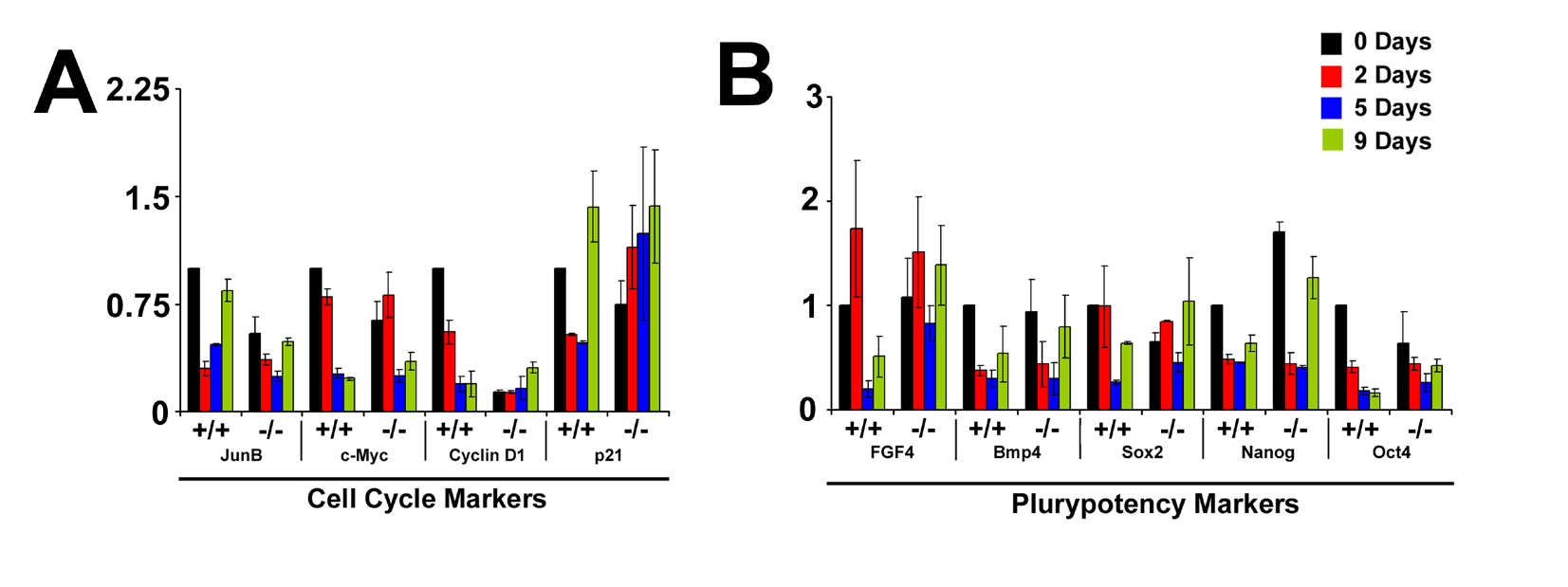

Supplement: Figure S16 — Bptf-mutant embryoid bodies show little to modest deregulation of cell cycle and pluripotency markers. Gene expression was monitored from total RNA during a 9-day embryoid body differentiation time course. RT-PCR analysis for cell cycle markers (A), pluripotency markers (B). (0.93 MB TIF) [file pgen.1000241.s016.tif]

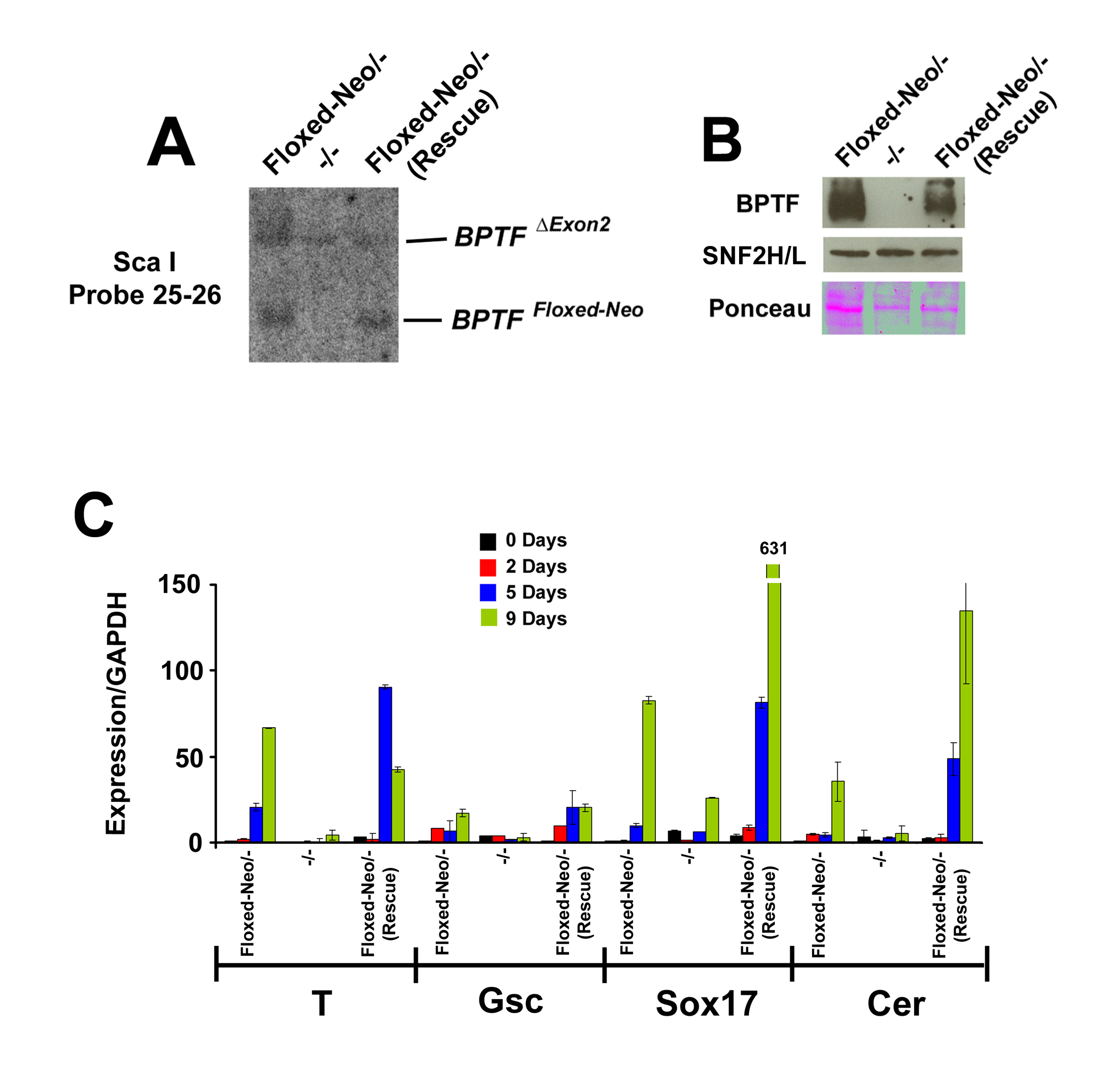

Supplement: Figure S17 — Transcription and differentiation defects in BptfΔExon2 knockout embryonic stem (ES) cells are rescued by reintroduction of BptfFloxed-Neo. (A) BptfΔExon2/BptfΔExon2 ES cell lines were retargeted with the BptfFloxed-Neo targeting vector to generate BptfΔExon2/BptfFloxed-Neo rescue lines. Recombination of the targeting vector at BptfΔExon2 was confirmed by Southern blotting Sca I digested DNA and probing with Probe 25–26. (B) A Western analysis of total protein from parental BptfΔExon2/BptfFloxed-Neo, BptfΔExon2/BptfΔExon2, and BptfΔExon2/BptfFloxed-Neo rescue lines show reintroduction of the Bptf protein. (C) Expression analysis of mesoderm markers T, Gsc, and endoderm markers Sox17, Cer in a BptfΔExon2/BptfFloxed-Neo rescue ES cell line during a 9-day embryoid body differentiation time course. Reintroduction of BptfFloxed-Neo rescued the induction of mesoderm and endoderm markers during embryoid differentiation. (0.90 MB TIF) [file pgen.1000241.s017.tif]

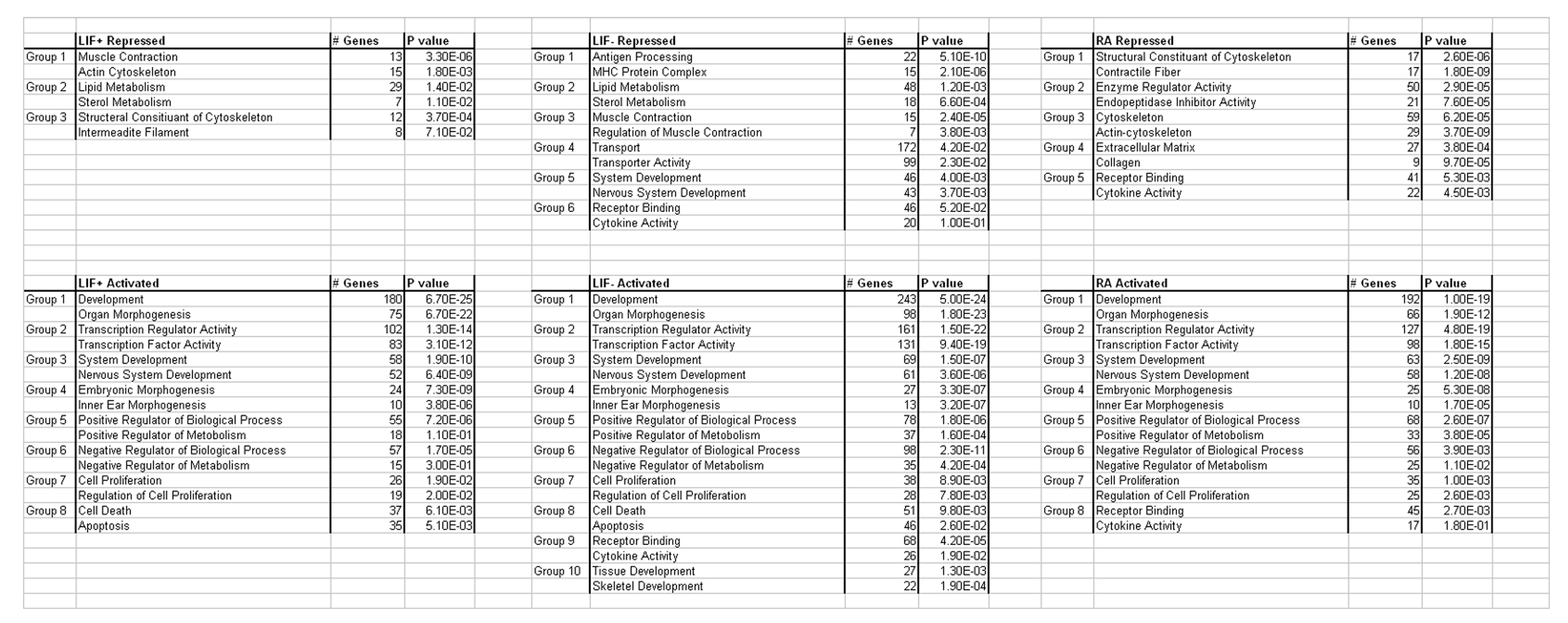

Supplement: Figure S18 — Gene ontology clustering analysis of Bptf-dependent genes by condition. The entire array dataset from each condition was analyzed for statistically significant ontology clusters using DAVID analysis software. Genes that were repressed in expression cluster to highly similar ontology groups and include those involved in “development”, “transcription factor activity”, and “morphogenesis”. Activated gene clusters are highly dependent on condition and include “antigen processing”, “peptidase inhibitors”, and “cytoskeletal components”. (0.88 MB TIF) [file pgen.1000241.s018.tif]

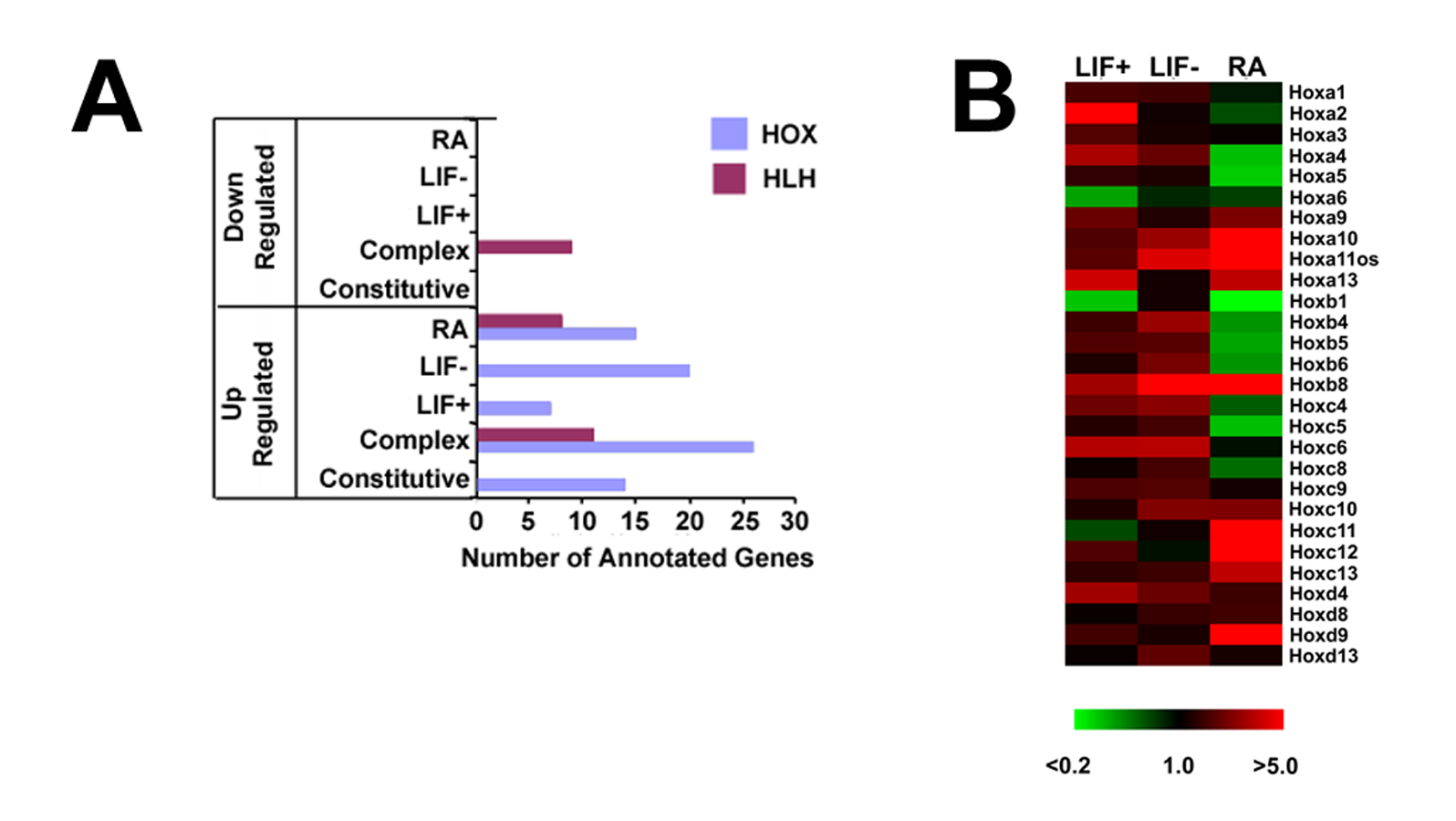

Supplement: Figure S19 — Bptf negatively regulates many homeobox transcription factors. (A) Genes containing a homeobox but not the helix loop helix domains are significantly enriched in all categories of up-regulated gene sets. (B) Expression levels of homeobox transcription factor gene clusters displayed as a heat map. Almost the entire a, b, and c clusters are deregulated, suggesting that Bptf plays an important function in regulating clusters of homeobox domain–containing transcription factors. (0.35 MB TIF) [file pgen.1000241.s019.tif]

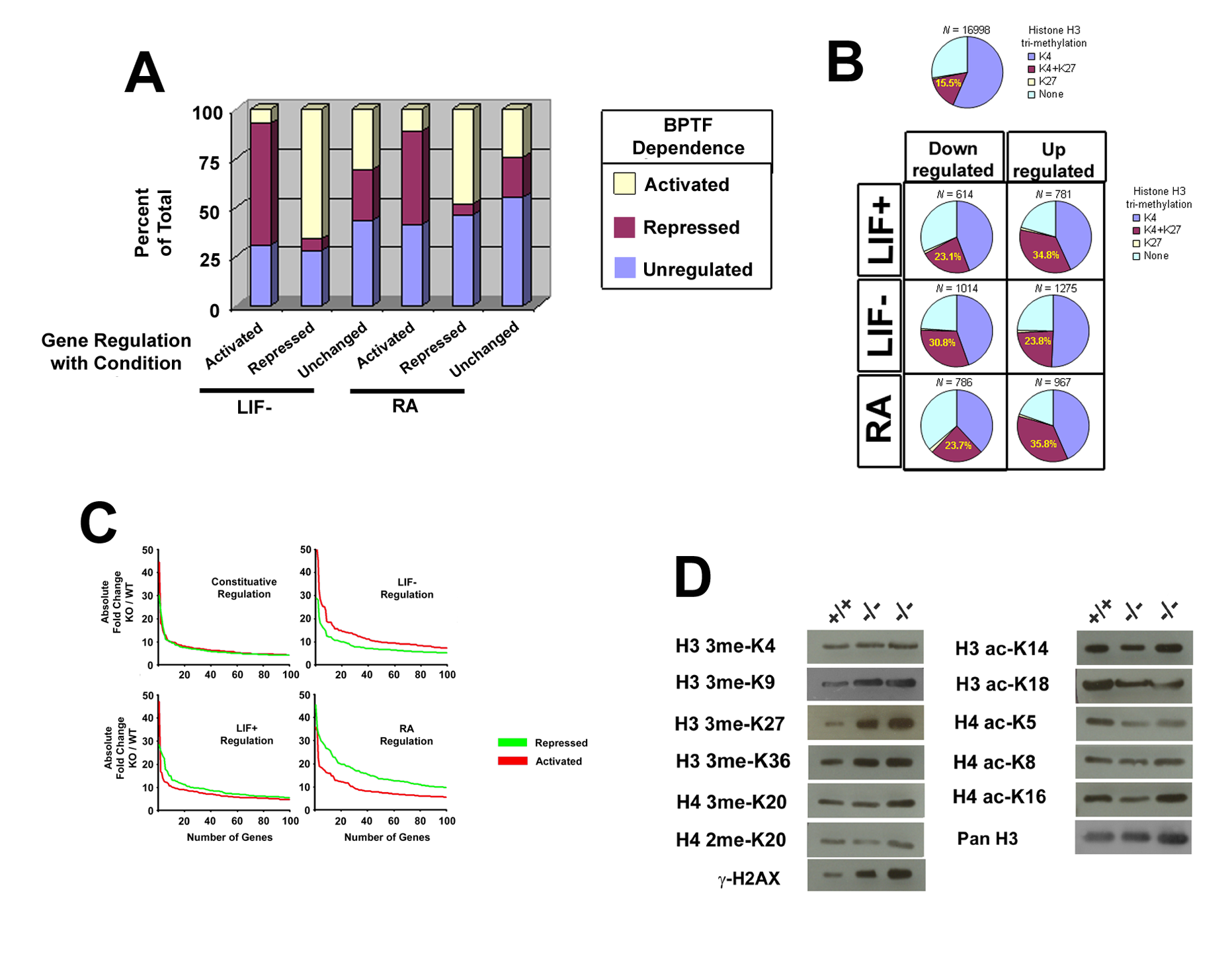

Supplement: Figure S20 — Bptf preferentially potentates dynamically regulated genes with bivalent promoters. (A) Genes whose transcription activates, represses, or is unchanged upon LIF- conditions or the addition of retinoic acid (RA) were analyzed for Bptf dependence. Knockout of Bptf results in predominantly reduced activation (repression of activated genes with Bptf knockout) or reduced repression (activation of repressed genes with Bptf knockout) of dynamically regulated genes. (B) Bptf preferentially regulates promoters with bivalent histone modifications. The methylation state expressed as a percentage of all promoters from undifferentiated mouse ES cells. Data was retrieved from Bernstein et. al. (2006) Cell 125:315–326. Bptf-dependent genes from the microarray dataset were grouped based on the promoter's methylation state. From this analysis, Bptf-dependent genes are predominantly enriched for bivalent histone H3 3me-K27, histone H3 3me-K4 over single modified or unmodified promoters. (C) The magnitude of deregulation was compared between activated and repressed genes for each condition of Bptf dependence. A breakdown of gene classes by condition shows that the transcriptome is repressed under LIF+ and RA growth conditions and activated under LIF-. (D) The modifications on bulk histones in Bptf mutant embryonic stem (ES) cells are consistent with a transcriptionally repressed chromatin state. Bulk histones were prepared from wild type (+/+) and two Bptf mutant (−/−) ES cell lines grown in LIF+ growth media. Total protein extracts were then probed for histone modifications by Western blotting. Bptf knockout ES cells have an increase of the repressive H3 3me-K9 and H3 3me-K27 and a decrease of the activating marks H3 ac-K18 and H4 ac-K5. Interestingly, knockout ES cells have an increase in levels of γ-H2AX, a mark correlated with DNA damage, and H3 3me-K36, a mark of transcription elongation. (0.97 MB TIF) [file pgen.1000241.s020.tif]

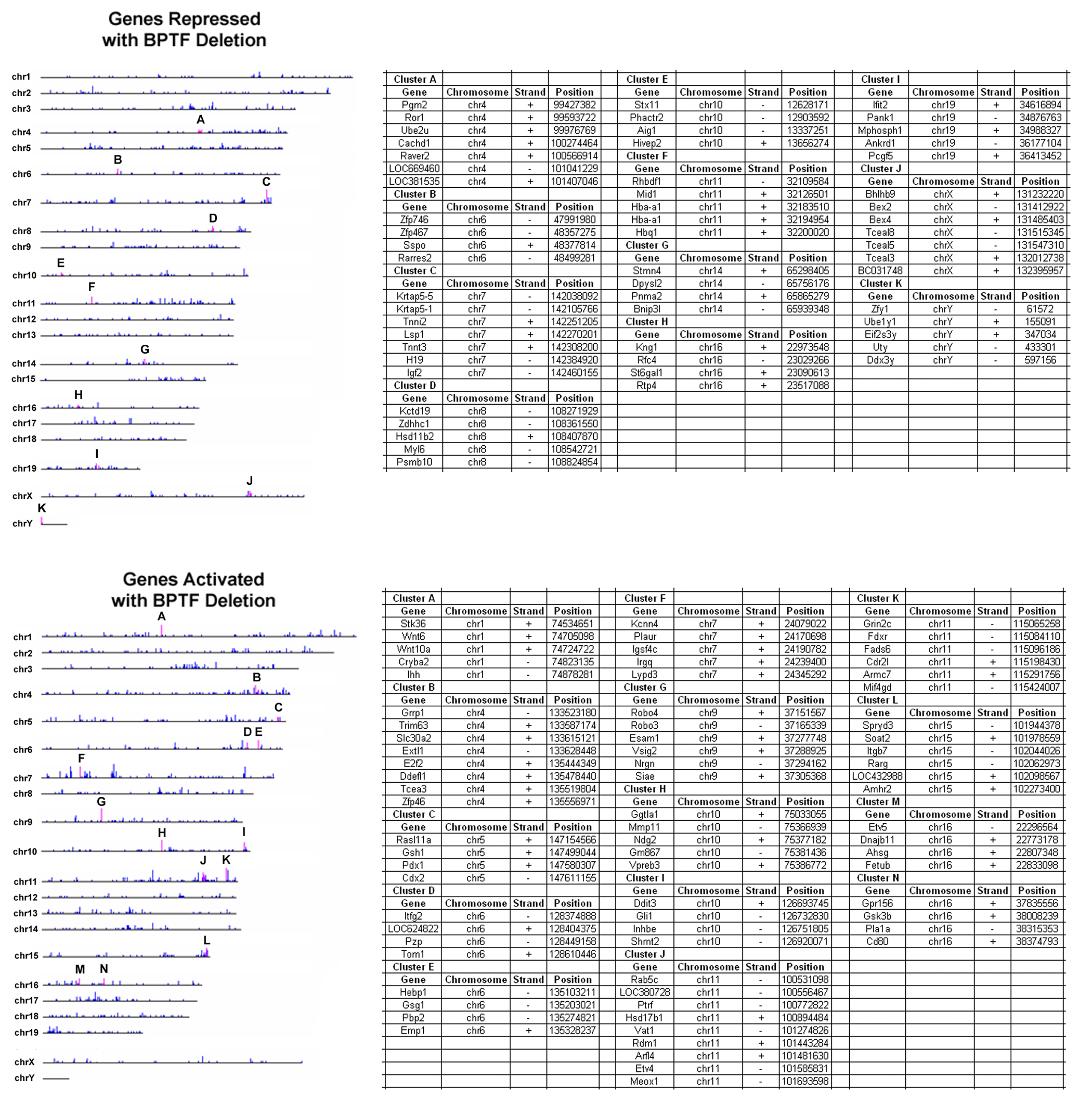

Supplement: Figure S21 — Bptf-dependent genes are frequently located in clusters. The chromosome position for each Bptf-dependent gene was identified, and statistically significant gene clusters are shown with a letter designation. Clusters include genes with similar regulation and imprinted clusters. (1.80 MB TIF) [file pgen.1000241.s021.tif]

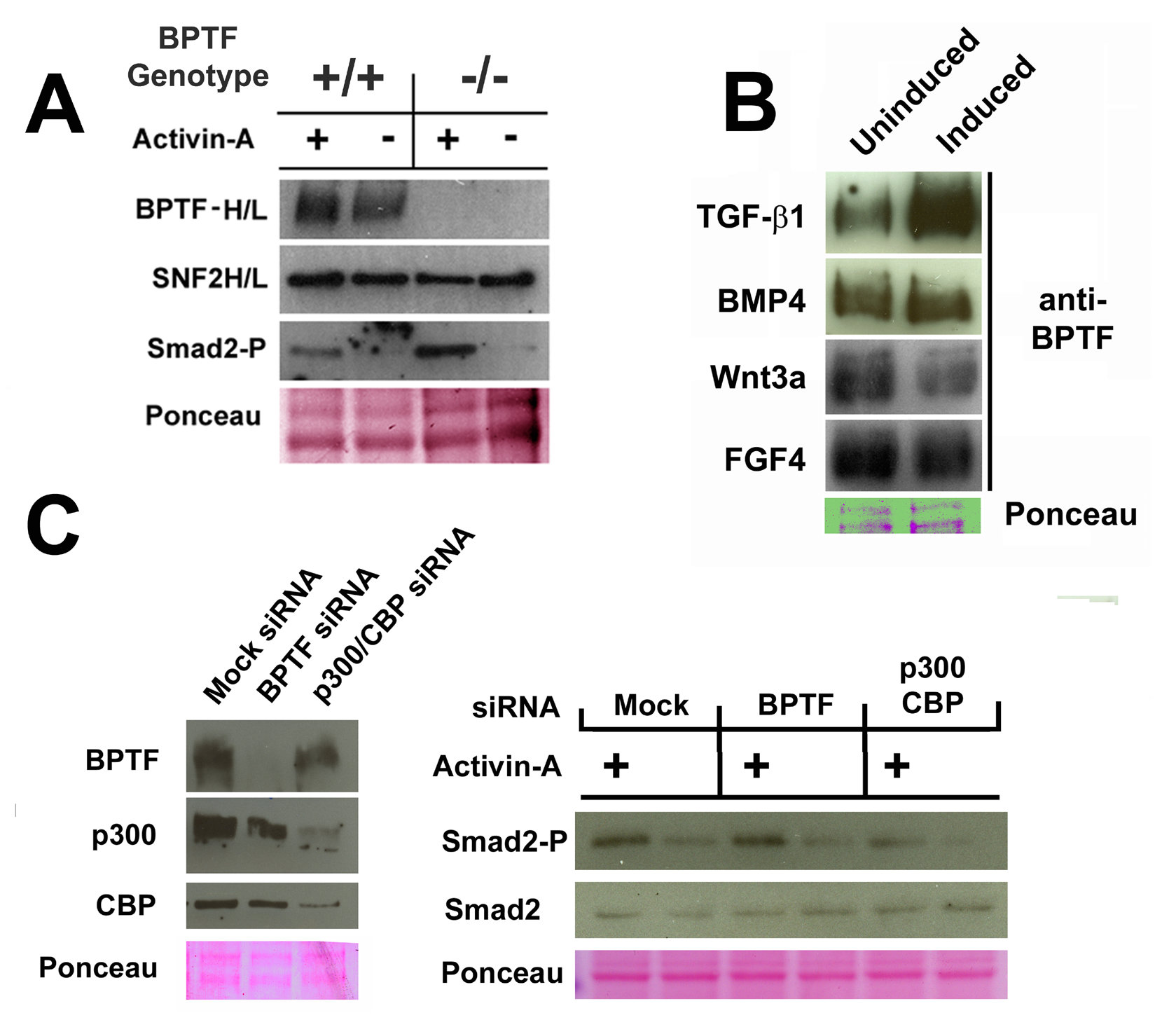

Supplement: Figure S22 — Western analysis of Bptf, CBP/p300 knockdown and Smad2 activation during TGF-β1 and activin-A stimulation. (A) Wild type and Bptf mutant embryonic stem (ES) cells were induced with activin-A for 24 hours. Western analysis shows up-regulation of Bptf protein levels and the phosphorylation of Smad2 in wild type and Bptf mutant ES cells with the addition of activin-A. (B) TGF-β1 selectively increases protein levels of Bptf in P19 cells. P19 cells were treated with TGF-β1, BMP4, Wnt3a-conditioned media, and FGF4+heparin for 24 hours. Following induction, cells were harvested and analyzed for Bptf levels by Western blotting. TGF-β1 increased and Wnt3a decreased Bptf protein levels. (C) Wild type ES cells were subjected to siRNA mediated knockdown of Bptf and CBP/p300, followed by induction with activin-A. Western analysis shows siRNA mediated knockdown of Bptf and CBP/p300 and phosphorylation of Smad2 with the addition of activin-A. (1.28 MB TIF) [file pgen.1000241.s022.tif]

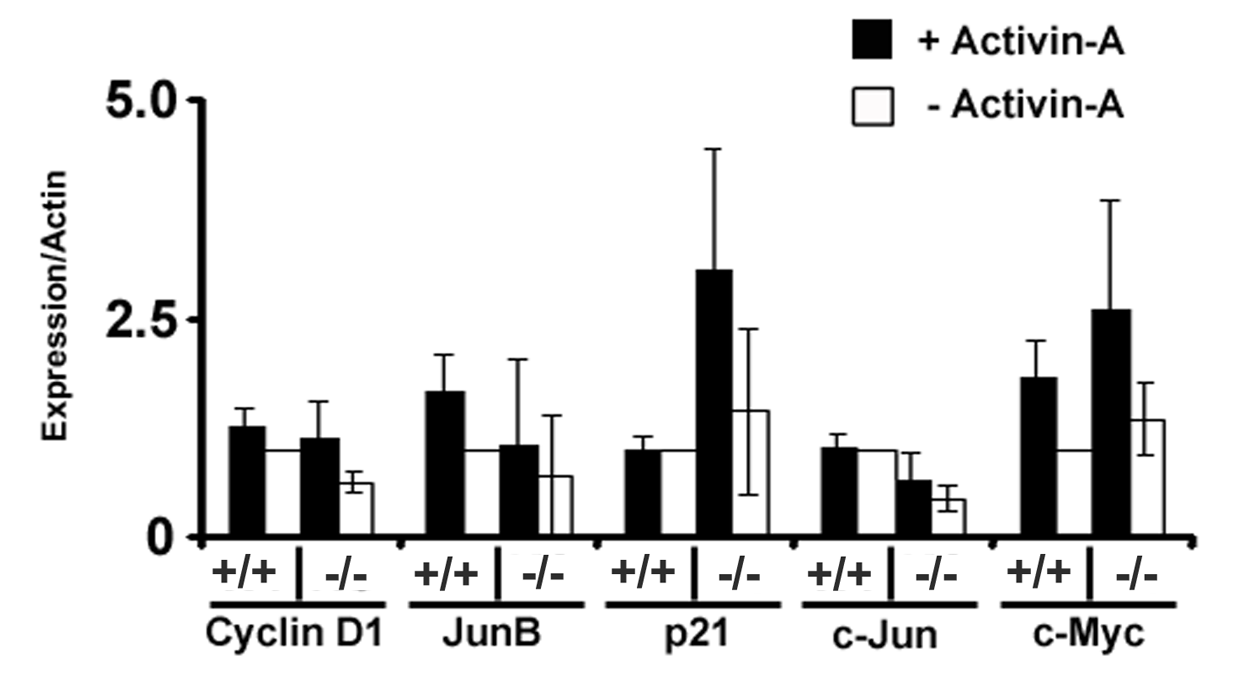

Supplement: Figure S23 — Bptf is necessary for Smad mediated gene transcription of cell cycle regulators. RT-PCR analysis of Smad target genes from activin-A–induced embryonic stem cells identifies Bptf-dependent and -independent gene targets. The repression of p21 during stimulation with activin-A is dependent on Bptf. (0.21 MB TIF) [file pgen.1000241.s023.tif]

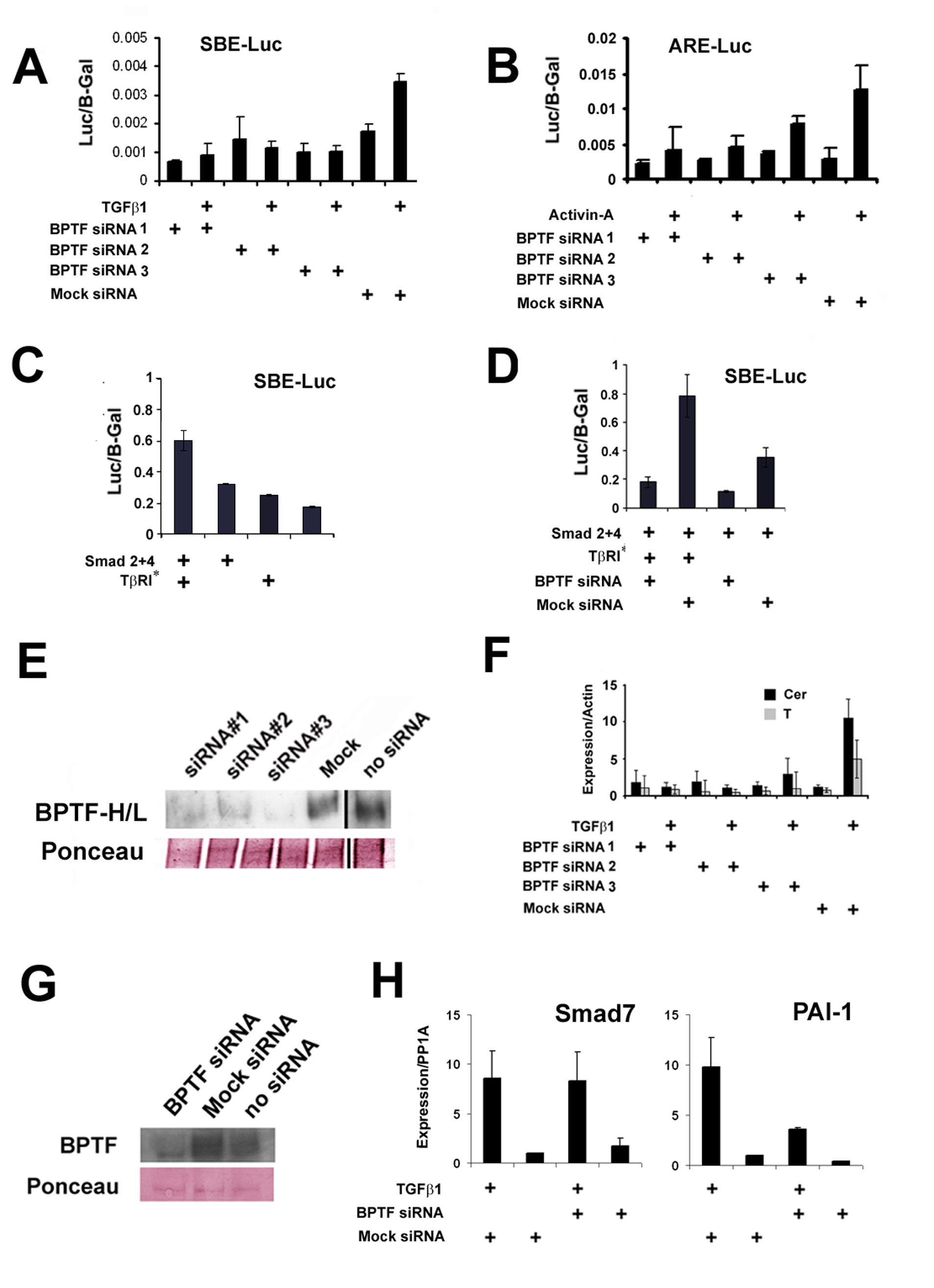

Supplement: Figure S24 — Bptf is necessary for activation of Smad-responsive promoter elements. (A) Knockdown of Bptf in P19 cells renders Smad responsive Smad binding elements (SBE) unresponsive to Smad activation via TGF-β. Luciferase activity was measured from stimulated or unstimulated P19 cells transfected with Bptf or mock siRNAs in addition to pGL3ti-(SBE)4 and LacZ transfection control plasmid. (B) Knockdown of Bptf in P19 cells renders Smad responsive ARE elements unresponsive to Smad activation via activin-A. Luciferase activity was measured from stimulated or unstimulated P19 cells transfected with Bptf or mock siRNAs in addition to pA3-Luc, CMV-Fast2-myc, and LacZ transfection control plasmid. (C) Co-transfection of HA-Smads 2,4 and constitutively active TβRI results in increased activation of luciferase reporter regulated by the SBE. (D) Knockdown of Bptf in P19 cells renders SBE Smad responsive elements unresponsive to constitutively activated Smad transcription factors. Luciferase activity was measured from P19 cells transfected with Bptf or mock siRNAs and combinations of Smad transcription factors, constitutively active type I receptor TβRI*, pGL3ti-(SBE)4, and a LacZ transfection control plasmid. (E) Western analysis showing siRNA mediated knockdown of Bptf in P19 cells during TGF-β1 induction. (F) Some TGF-β1-responsive gene targets in P19 cells are Bptf-dependent. Bptf knockdown during TGF-β1 activation significantly reduces the Smad mediated gene activation of Cer and T. (G) Western analysis showing siRNA mediated knockdown of BPTF in MCF10CA1 cells during TGF-β1 induction. (H) BPTF knockdown during TGF-β1 activation significantly reduces the Smad mediated gene activation of PAI-1 but not SMAD7. (1.16 MB TIF) [file pgen.1000241.s024.tif]

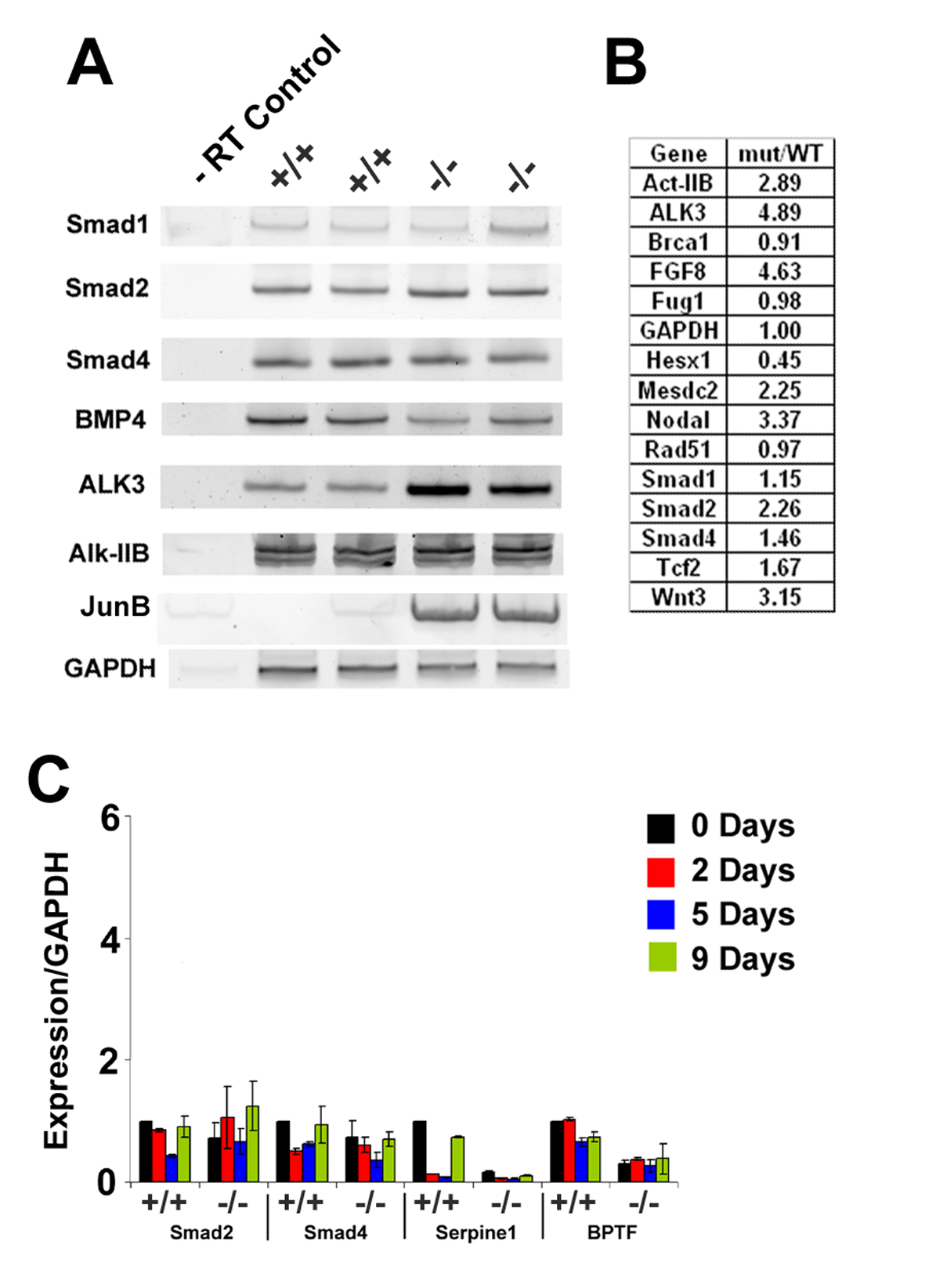

Supplement: Figure S25 — Bptf-mutant embryos likely do not down regulate the Smad signaling pathway indirectly. (A) RT-PCR analysis of E7.5 embryos shows normal expression of the BMP4 ligand, Smad 1,2 and 4 transcription factors, and receptors ALK3, Alk-IIB. Expression of the TGFβ-responsive gene JunB is significantly increased in mutant embryos. (B) Results from quantitative RT-PCR measurements of additional markers of early embryonic development in E7.5 embryos. Expression has been normalized to GAPDH levels and is expressed as the ratio mutant/WT. (C) An RT-PCR analysis of wild type and Bptf-mutant embryoid body differentiation time course for Smad transcription factors, Bptf, and the Smad responsive gene Serpine1. (0.64 MB TIF) [file pgen.1000241.s025.tif]

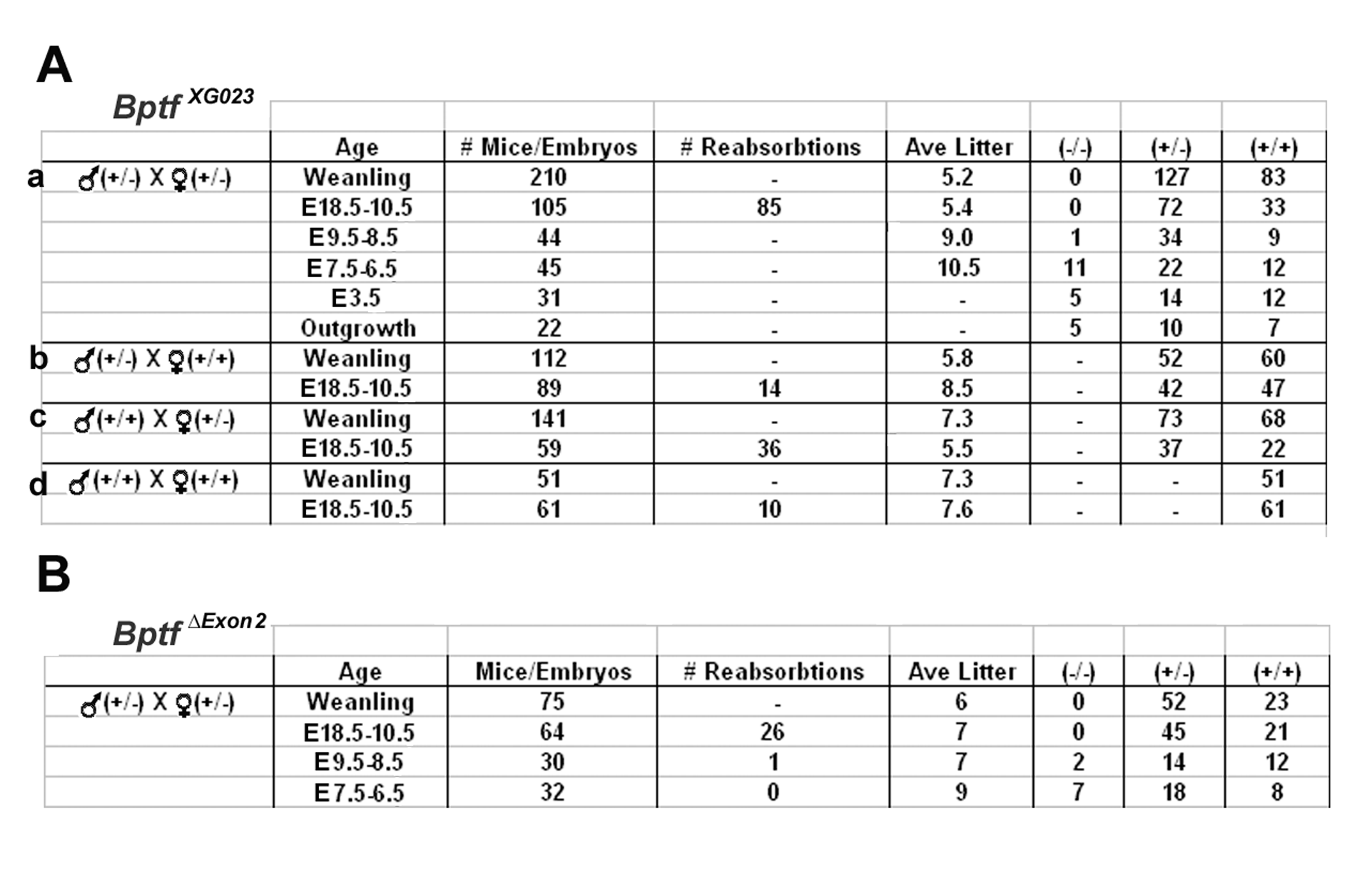

Supplement: Table S1 — Disruption of Bptf results in early embryonic lethality. (A) Wild type (+/+) and heterozygous (+/−) BptfXG023 males and females were mated in reciprocal crosses and all progeny were genotyped by Southern blotting or PCR. Homozygous (−/−) BptfXG023 embryos were not viable after E7.5 to E8.5, showing that mutations in Bptf are early embryonic lethal. Curiously, more reabsorptions were observed during the dissections than could be accounted for from the death of the homozygous BptfXG023 embryos. To investigate if the origin of the increased lethality was caused by a parental BptfXG023 heterozygous contribution, we crossed heterozygous to wild type males and females in combinations and examined all embryos from E10.5 to E18.5. From these experiments, we observed an increased number of reabsorptions when the mother was heterozygous compared to the wild type controls (Table S1A, b–d). The embryonic lethality was not biased toward heterozygous embryos, as they were found in the expected 1:1 ratio to wild type. These data suggested that BptfXG023 conferred a maternal effect on embryo survival. (B) As in (A), heterozygous (+/−) BptfΔExon2 males and females were intercrossed and all progeny were genotyped by Southern blotting or PCR. As with BptfXG023, homozygous (−/−) BptfΔExon2 embryos were not viable after E7.5 to E8.5. (0.33 MB TIF) [file pgen.1000241.s026.tif]

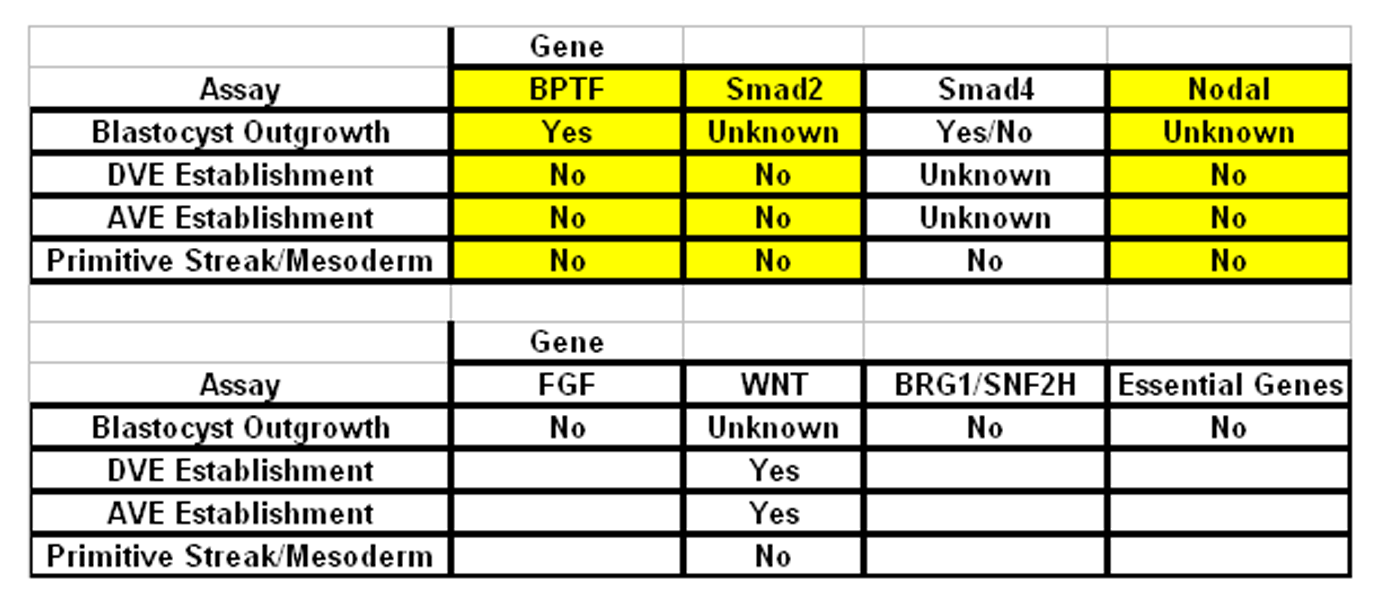

Supplement: Table S2 — Bptf mutants show highly similar phenotypes to Smad and Nodal mutants. Phenotypes of Bptf mutants during blastocyst outgrowth and the establishment of the DVE, AVE, and primitive streak were compared to phenotypes from mutations of Smad2/4, Nodal, Wnt3/β-catenin, remodelers BRG1/SNF2H, and essential genes. Bptf mutations closely resemble mutations in Smad and Nodal pathways. (0.30 MB TIF) [file pgen.1000241.s027.tif]

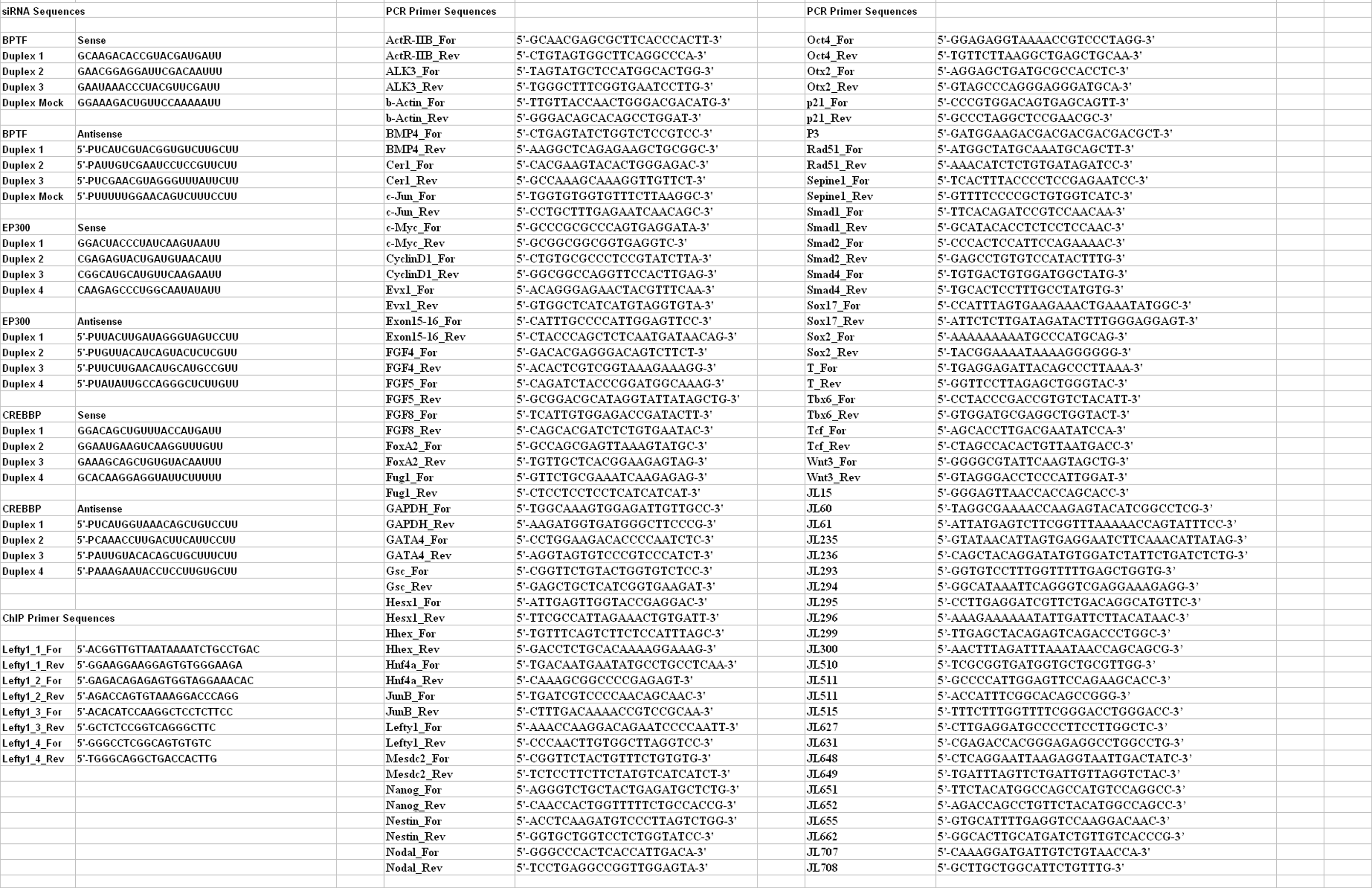

Supplement: Table S3 — Primer and siRNA sequences used in study. Sequences of all siRNA duplexes and primers used in this study are shown in table format. (1.47 MB TIF) [file pgen.1000241.s028.tif]
